# Global, regional, and national burden of liver cancer in adolescents and young adults from 1990 to 2021: an analysis of the global burden of disease study 2021 and forecast to 2040

**DOI:** 10.3389/fpubh.2025.1547106

**Published:** 2025-03-10

**Authors:** Jingyu Wen, Mingge Xia, Han Luo, Luwei Zhu, Min Li, Yifu Hou

**Affiliations:** ^1^Department of Medical Insurance, Sichuan Provincial People's Hospital, University of Electronic Science and Technology of China, Chengdu, China; ^2^State Key Laboratory of Quality Research in Chinese Medicines, Institute of Chinese Medical Sciences, University of Macau, Taipa, Macao SAR, China; ^3^Department of Hepatobiliary Surgery, Zigong Fourth People's Hospital, Zigong, China; ^4^Department of Organ Transplantation, Sichuan Provincial People's Hospital, University of Electronic Science and Technology of China, Chengdu, China; ^5^School of Medicine, University of Electronic Science and Technology of China, Chengdu, China

**Keywords:** global burden of disease study, adolescents and young adults, liver cancer, NASH, predict

## Abstract

**Background:**

The global burden of liver cancer among adolescents and young adults (AYAs) has often been underestimated, despite significant shifts in its etiology. This study analyzes the disease burden of liver cancer in AYAs from 1990 to 2021 and forecasts trends up to 2040 using data from the Global Burden of Disease Study 2021. Our goal is to provide insights that can inform resource allocation and policy planning.

**Methods:**

Incidence, mortality, and disability-adjusted life years (DALYs) data were extracted and estimated annual percentage changes calculated to assess trends. Correlation between age-standardized rates and sociodemographic index (SDI) was analyzed using Spearman correlation, and future trends were predicted using the Bayesian age-period-cohort model.

**Findings:**

Globally, there were 24,348 new liver cancer cases and 19,270 deaths among AYAs in 2021, with decreases in age-standardized rates for incidence, mortality, and DALYs from 1990 to 2021. East Asia bears the highest burden, with males experiencing significantly higher rates than females. The burden increases with age, peaking at 35–39 years. Higher SDI is associated with lower incidence, mortality, and DALYs. While HBV remains the leading cause, NASH is the fastest-growing contributor to liver cancer incidence and mortality. Projections indicate a continued decline in liver cancer burden among AYAs, though female cases are expected to rise.

**Interpretation:**

Despite a gradual decline in liver cancer burden among AYAs, NASH is emerging as a significant and rising cause of incidence and mortality. Regional and gender disparities persist, highlighting the need for tailored prevention and healthcare strategies to alleviate the liver cancer AYA's burden globally.

## Introduction

Liver cancer (LC) is the sixth most common cancer worldwide and one of the deadliest, posing a significant public health challenge (1). Traditionally, LC occurs primarily in individuals over 50, largely due to its strong association with the prolonged progression of chronic liver diseases (2). These conditions gradually cause persistent inflammation and liver damage, eventually leading to LC. However, recent data show a concerning increase in cancer incidence among adolescents and young adults (AYAs), especially in Western countries (3). This demographic often exhibits more aggressive tumor biology, resulting in more advanced disease stages at diagnosis compared to older populations (4, 5). Despite advances in early detection and treatment, prognosis remains poor for AYAs, especially when diagnosed at later stages.

In recent years, the causes of LC have changed significantly, especially in developed countries and among AYAs. Traditionally, viral hepatitis (such as HBV and HCV) was the primary cause of LC. However, with the widespread implementation of HBV vaccination and antiviral treatments, the incidence of LC due to viral hepatitis has gradually declined (6). Conversely, non-viral causes, particularly non-alcoholic fatty liver disease (NAFLD), especially non-alcoholic steatohepatitis (NASH), are becoming increasingly significant contributors to LC in AYA, especially against a backdrop of rising obesity and metabolic syndrome rates (7). However, in the 2019 Global Burden of Disease (GBD) study, the absence of an International Classification of Diseases (ICD) code specifically for NASH led to insufficient recognition of NASH as a significant non-viral etiology, particularly as its prevalence continues to rise (8). Consequently, NASH may have been underrepresented and potentially overlooked in the analysis. Given the changing causes of LC and its aggressive nature in AYAs, it is essential to develop effective prevention and treatment strategies specifically for this age group. This approach will help address the underlying factors contributing to this troubling trend and its wider public health implications.

Despite changes in the causes of LC, researchers continue to focus on the molecular mechanisms affecting AYA, although comprehensive assessments of the global disease burden in this group are very limited (9). More studies have explored the population-wide burden of LC or the global cancer burden in AYAs, but these studies often do not provide a comprehensive assessment of LC in this particular population, overlooking AYA's unique epidemiology, clinical care, and social impact, as well as important heterogeneity (10–12). At the same time, the existing research data often have a time lag, and it is difficult to accurately grasp the prevalence and disease burden of LC in AYAs (13). Since AYAs are an important part of the workforce, effective interventions can greatly enhance their health and lower the global disease burden. The Global Burden of Disease (GBD) study systematically assesses the burden of LC across 204 countries and regions. It provides valuable insights into trends observed over the past 30 years. In this study, we used the most recent 2021 GBD data to estimate the incidence, mortality, and disability-adjusted life years (DALYs) associated with LC in AYAs.

This analysis aims to clarify the scope and severity of LC, inform prevention and control strategies, highlight public health challenges that may emerge by 2040, offering a timeline to help policymakers optimize global health resource allocation and develop proactive interventions to reduce the burden of LC among AYA. Additionally, it seeks to encourage scientific research and technological innovation while raising public health awareness. Ultimately, these efforts will improve the quality of life and address socioeconomic issues among AYAs.

## Methods

### Study population and data collection

In this study, we obtained data on LC from the Global Burden of Diseases, Injuries, and Risk Factors Study (GBD) 2021. The GBD is the largest and most comprehensive global observational epidemiological project. It provided estimates of the global burden of 371 diseases and injuries in 204 countries and territories through extensive data collection, review, and analysis (14).

In GBD 2021, cancers are classified into various groups according to the ICD-10 (https://www.who.int/classifications/icd/en/). LC includes all diagnoses coded C22.0 to C22.9. Numbers and rates on the incidence, mortality and DALYs of LC were extracted from the GBD Results Tool (https://vizhub.healthdata.org/gbd-results/). The data were categorized by sex, age, region, and country. All cancer rates were reported per 100,000 person-years. The GBD world population standard was used for the calculation of age-standardized rates (ASRs). The definition of the age range for AYAs varies, especially at the upper limit (15–17). In this study, we defined this range as 15–39 years based on multidimensional considerations encompassing biomedicine, social psychology, and disease epidemiology. This range is endorsed by the US National Cancer Institute and the AYA Working Group of the European Society for Medical Oncology and the European Society for Pediatric Oncology (18), allowing for comparability with other studies on AYA cancer. However, there are significant differences in the definitions of AYAs among different studies and institutions. The World Health Organization classifies the age range as 10–24 years, based on the onset of puberty to skeletal maturity. There are also specific research cases that set the age range to under 50 years (19) or 15–29 years (20), which are driven by the research objectives. In this study, ages in the range 15–39 years were further divided into five GBD age groups at 5-year intervals.

The socio-demographic Index (SDI) in GBD 2021 represents the combined health-related social and economic conditions of each region. It's a composite measure of total fertility rate in females younger than 25 years, average educational attainment for individuals aged 15 and older, and age-distributed income per capita. The 204 countries in GBD 2021 were grouped into quintiles (low, low-middle, middle, high-middle, and high) based on country-level estimates of SDI.

### Statistical analysis

ASRs per 100,000 people were extracted from the GBD database. The ASR was calculated using the formula: ASR = Σi N = 1 αi W/Σi N = 1 Wi, where αi denotes the age-specific rate in the *i*th age group and w_*i*_ represents the number of people (or the weight) in the same age group among the GBD standard population. N is total the number of age groups. The 95% uncertainty intervals (UIs) were defined as the 25th and 975th values of the ordered 1,000 draws.

The estimated annual percentage change (EAPC) in ASR was calculated to evaluate the average changing trends over a specified time interval, and was widely used in secondary analysis based on GBD. We assumed natural logarithm of ASR fit the linear regressions model y = α + βx + ε, where y is equal to ln (ASR), and x refers to the calendar year. Then, the EAPC is equal to 100 × (eβ-1). Ninety-five percentage CIs of EAPC were estimated using the linear regression model. An ASR indicates an increasing trend if both the EAPC and its 95% confidence interval (CI) are above zero. Conversely, it indicates a decreasing trend if both are below zero. If the 95% CI includes zero, the trend is considered statistically insignificant. The proportion of LC cases secondary to each etiology was calculated for each study, and the pooled proportions were then used in five separate DisMod-MR 2.1 models (a Bayesian meta regression-type model) to determine the overall proportion of LC due to the five defined etiologies.

We used smoothing splines models/local regression smoothing models (loess) to evaluate the relationship between the burdens of LC among AYA and SDI for the 21 regions and 204 countries and territories. A *p* < 0.05 was considered statistically significant. Furthermore, given that the distribution of SDI across countries changed a lot from 1990 to 2021, we computed the EAPC of SDI of 204 countries and employed Spearman correlation analysis to examine the relationship between the EAPCs of SDI and ASRs. A *p* < 0.05 was considered statistically significant. All statistical analyses and graphical representations were performed using R software (version 3.6.0).

### BAPC model projection

The Bivariate Age-Period-Cohort (BAPC) model was applied to predict the future disease burdens of LC among AYA due to its flexibility and robustness in handling time series data. The BAPC model is especially useful for long-term disease burden forecasting. Its ability to capture temporal trends and offer comprehensive coverage has made it widely validated and applied in large-scale epidemiological studies like GBD (21). The BAPC model builds on the traditional generalized linear model (GLM) framework within a Bayesian context. It assumes that the relationships between age, period, and cohort are linear and that these effects are independent of each other. However, this assumption can be challenged by interactions between these effects. To address potential biases, the model often incorporates smoothing techniques or constraints to allow for flexibility while maintaining stability in the estimates. In this study, effects are assumed to evolve continuously over time and are smoothed using a second-order random walk, resulting in more accurate posterior probability predictions. A key strength of the BAPC model is its use of the Integrated Nested Laplace Approximation (INLA) method to approximate the marginal posterior distribution. This method effectively avoids issues like mixing and convergence problems commonly encountered with Markov Chain Monte Carlo techniques, while ensuring computational efficiency.

## Results

### Incidence, mortality, and DALYs among AYA in 2021

#### Incidence

**Figure 1:**
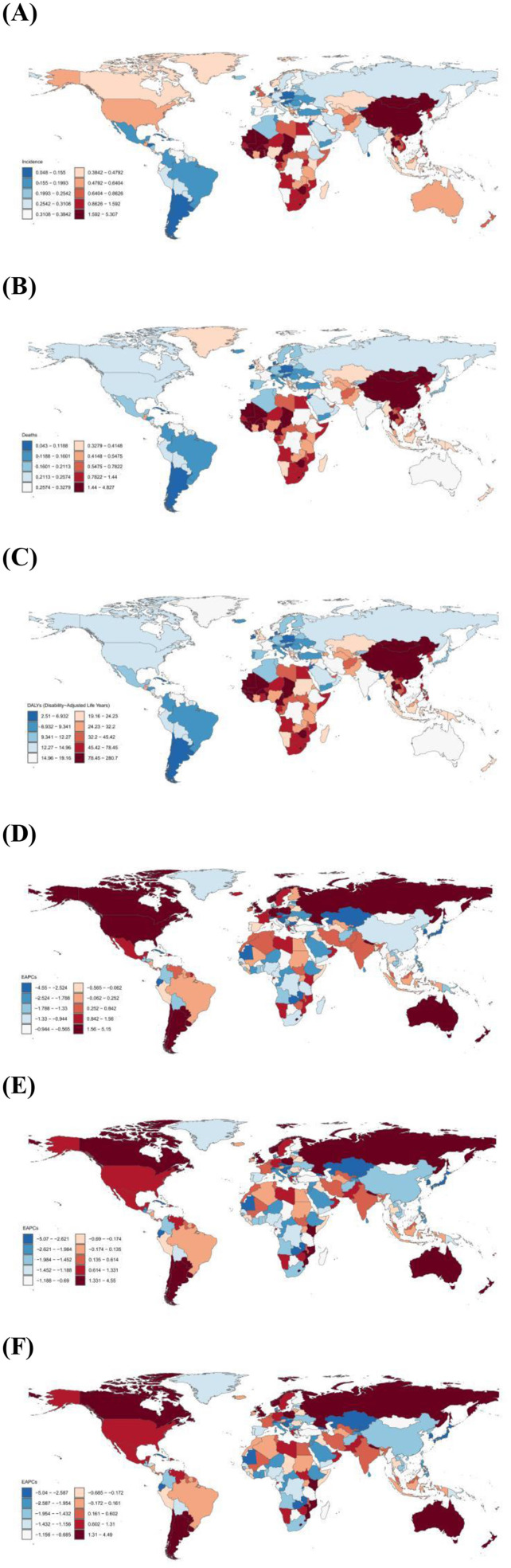
**(A)** Age-standardized incidence rate of liver cancer in AYA per 100,000 population, 2021; **(B)** Age-standardized mortality rate of liver cancer in AYA per 100,000 population, 2021; **(C)** Age-standardized DALYs rate of liver cancer in AYA per 100,000 population, 2021; **(D)** Estimated Annual percentage change in incidence rate of liver cancer in AYA, 1999–2021; **(E)** Estimated Annual percentage change in mortality rate of liver cancer in AYA, 1999–2021; **(F)** Estimated Annual percentage change in DALYs rate of liver cancer in AYA, 1999–2021.

Globally, an estimated 24,348 (95% UI 21,491 to 28,273) new LC cases among AYA aged 15–39 years were reported in 2021, with East Asia (12,148) having the highest number of new cases (Table 1). The global age-standardized incidence rate was 0.79 per 100,000 population, and the top five countries with the highest incidence included Gambia (ASR, 5.31), Mongolia (ASR, 4.62), Eswatini (ASR, 4.14), Guinea-Bissau (ASR, 3.68) and Mali (ASR, 3.48; Supplementary Table S1 and Figure 1A). At the regional level, East Asia had the highest incidence (ASR, 2.08), followed by Southern Sub-Saharan Africa (ASR, 1.50) and Western Sub-Saharan Africa (ASR, 1.34). Regarding sex differences, the age-standardized incidence rate for males (ASR, 1.19) was 3.1 times higher than that for females (ASR, 0.39), highlighting a significant disparity in LC incidence between genders. Furthermore, considering different sex subgroups, the highest age-standardized incidence rate was found in males of East Asia (ASR, 3.47). For female subgroups, the highest age-standardized incidence rate was recorded in Southern Sub-Saharan Africa (ASR, 1.03; Supplementary Table S2).

**Table 1:** Incidence, mortality and DALYs of liver cancers among AYA in 1990 and 2021, and their estimated annual percentage changes from 1990 to 2021.

**Characteristics**	**Incidence**					**Mortality**					**DALYs**				
	**Number of cases, 1990 **	**Age-standardized rate per 100,000 population, 1990 **	**Number of cases, 2021 **	**Age-standardized rate per 100,000 population, 2021 **	**Estimated annual percentage change, 1990–2021 **	**Number of cases, 1990 **	**Age-standardized rate per 100,000 population, 1990 **	**Number of cases, 2021 **	**Age-standardized rate per 100,000 population, 2021 **	**Estimated annual percentage change, 1990–2021 **	**Number of cases, 1990 **	**Age-standardized rate per 100,000 population, 1990 **	**Number of cases, 2021 **	**Age-standardized rate per 100,000 population, 2021 **	**Estimated annual percentage change, 1990–2021 **
Global	19863.66 (17389.28–22885.63)	0.98 (1.14 to 0.85)	24348.06 (21491.13–28273.11)	0.79 (0.93 to 0.69)	−1.25 (−1.48 to −1.01)	17748.11 (15531.71–20436.12)	0.87 (1.01 to 0.76)	19270.22 (17036.81–22393.10)	0.63 (0.74 to 0.55)	−1.60 (−1.86 to −1.34)	1027745.96 (900150.49–1183734.76)	49.98 (58.29 to 43.49)	1108669.92 (980707.98–1285863.91)	36.28 (42.45 to 31.71)	−1.60 (−1.86 to −1.34)
**Sex**															
Female	4981.46 (4282.47–5756.78)	0.49 (0.57 to 0.41)	5872.29 (5288.76–6567.48)	0.39 (0.45 to 0.34)	−1.15 (−1.32 to −0.99)	4479.47 (3843.55–5185.38)	0.44 (0.51 to 0.37)	4810.98 (4313.35–5380.01)	0.32 (0.37 to 0.28)	−1.43 (−1.62 to −1.24)	265623.52 (228012.08–307368.84)	25.70 (30.21 to 21.72)	283954.63 (254321.55–317365.19)	18.99 (21.74 to 16.69)	−1.41 (−1.60 to −1.22)
Male	14882.21 (12790.91–17478.66)	1.45 (1.72 to 1.23)	18475.76 (15857.68–22363.29)	1.19 (1.45 to 1.01)	−1.26 (−1.52 to −1.00)	13268.64 (11403.00–15595.02)	1.29 (1.54 to 1.10)	14459.24 (12391.99–17496.62)	0.93 (1.13 to 0.79)	−1.65 (−1.94 to −1.36)	762122.44 (656387.79–895846.91)	73.63 (87.93 to 62.57)	824715.29 (707716.86–996500.36)	53.21 (64.53 to 45.10)	−1.65 (−1.94 to −1.36)
**GBD regions**															
East Asia	12515.90 (10403.11–14950.78)	2.40 (2.91 to 1.98)	12148.88 (9606.79–15553.45)	2.08 (2.68 to 1.62)	−1.12 (−1.47 to −0.77)	11235.41 (9357.86–13417.81)	2.15 (2.60 to 1.78)	8883.85 (7029.84–11422.96)	1.53 (1.96 to 1.18)	−1.76 (−2.13 to −1.38)	645367.01 (537342.96–770519.79)	122.77 (148.93 to 101.53)	501526.04 (397414.92–644874.01)	87.00 (111.96 to 67.39)	−1.78 (−2.17 to −1.40)
Southeast Asia	1624.22 (1408.47–1899.89)	0.93 (1.14 to 0.77)	2189.89 (1782.72–2922.32)	0.77 (1.05 to 0.59)	−1.02 (−1.15 to −0.90)	1489.61 (1290.16–1746.27)	0.85 (1.03 to 0.70)	1905.02 (1547.15–2545.24)	0.67 (0.92 to 0.52)	−1.13 (−1.28 to −0.98)	86658.96 (75017.63–101344.46)	48.86 (59.47 to 40.39)	109074.95 (88261.20–145364.07)	38.29 (52.66 to 29.60)	−1.13 (−1.28 to −0.98)
Central Asia	203.18 (180.32–230.73)	0.76 (0.88 to 0.67)	226.71 (187.40–268.43)	0.57 (0.69 to 0.46)	−1.36 (−1.58 to −1.14)	188.27 (166.98–213.48)	0.71 (0.81 to 0.61)	207.10 (171.19–245.73)	0.52 (0.63 to 0.42)	−1.42 (−1.63 to −1.20)	11158.84 (9925.99–12675.57)	41.36 (47.66 to 36.04)	12035.35 (9962.15–14294.99)	30.50 (37.07 to 24.78)	−1.45 (−1.66 to −1.23)
South Asia	986.27 (873.35–1161.16)	0.25 (0.30 to 0.22)	2503.08 (2161.53–2999.50)	0.33 (0.40 to 0.27)	0.67 (0.46–0.88)	914.51 (809.93–1072.66)	0.23 (0.28 to 0.20)	2265.34 (1957.59–2712.57)	0.29 (0.36 to 0.25)	0.65 (0.44–0.86)	54180.98 (47985.12–63140.63)	13.58 (16.31 to 11.69)	132492.66 (114372.92–158786.19)	17.14 (21.07 to 14.37)	0.62 (0.41–0.84)
Oceania	13.57 (7.70–27.02)	0.59 (1.21 to 0.32)	24.36 (15.35–43.98)	0.46 (0.87 to 0.27)	−0.91 (−1.06 to −0.77)	12.49 (7.12–24.93)	0.54 (1.12 to 0.29)	22.09 (13.82–40.01)	0.42 (0.79 to 0.24)	−0.99 (−1.13 to −0.85)	739.16 (418.36–1467.75)	31.13 (64.94 to 16.72)	1297.71 (812.81–2340.59)	24.24 (45.85 to 14.22)	−0.98 (−1.12 to −0.85)
Australasia	19.54 (17.54–21.58)	0.23 (0.28 to 0.20)	61.31 (53.49–70.68)	0.53 (0.66 to 0.43)	2.62 (2.50–2.75)	15.10 (13.65–16.68)	0.18 (0.21 to 0.15)	37.64 (33.25–43.05)	0.33 (0.40 to 0.27)	1.74 (1.51–1.96)	884.27 (802.03–973.17)	10.59 (12.60 to 8.94)	2177.52 (1928.78–2474.93)	19.18 (23.34 to 15.81)	1.71 (1.49–1.94)
Eastern Europe	182.35 (173.16–191.78)	0.20 (0.22 to 0.19)	195.28 (180.58–209.91)	0.24 (0.26 to 0.22)	0.60 (0.06–1.14)	165.76 (157.78–174.36)	0.18 (0.20 to 0.17)	170.15 (157.29–182.78)	0.21 (0.23 to 0.20)	0.56 (−0.05–1.17)	9784.62 (9321.41–10276.65)	10.92 (11.79 to 10.16)	9748.14 (9038.55–10461.60)	12.57 (13.64 to 11.57)	0.45 (−0.13–1.04)
Central Europe	129.86 (118.53–142.56)	0.26 (0.29 to 0.23)	67.10 (59.89–75.04)	0.17 (0.19 to 0.15)	−1.72 (−2.07 to −1.36)	117.96 (107.77–129.46)	0.24 (0.27 to 0.21)	58.49 (51.96–65.28)	0.15 (0.17 to 0.13)	−1.81 (−2.20 to −1.41)	6875.06 (6287.28–7541.26)	13.97 (15.68 to 12.53)	3376.34 (3003.02–3757.54)	8.61 (9.84 to 7.53)	−1.79 (−2.19 to −1.39)
Western Europe	356.85 (343.01–371.03)	0.24 (0.26 to 0.23)	526.01 (499.53–555.59)	0.37 (0.40 to 0.34)	1.39 (1.27–1.51)	273.67 (263.38–284.63)	0.19 (0.20 to 0.17)	306.86 (292.48–320.86)	0.22 (0.23 to 0.20)	0.43 (0.35–0.51)	16122.62 (15515.70–16736.97)	10.97 (11.69 to 10.31)	17827.13 (17008.88–18635.19)	12.73 (13.66 to 11.90)	0.41 (0.33–0.50)
High-income Asia Pacific	887.08 (698.69–1127.76)	1.29 (1.79 to 0.95)	399.11 (326.51–493.50)	0.67 (0.87 to 0.52)	−2.88 (−3.17 to −2.58)	704.45 (540.77–910.35)	1.03 (1.45 to 0.73)	205.76 (169.91–258.74)	0.35 (0.45 to 0.27)	−4.37 (−4.67 to −4.06)	40043.71 (30818.95–51562.94)	58.52 (82.71 to 41.53)	11550.58 (9596.36–14421.28)	19.64 (25.69 to 15.41)	−4.34 (−4.64 to −4.05)
High-income North America	325.55 (316.88–334.33)	0.27 (0.28 to 0.26)	618.05 (589.23–648.16)	0.48 (0.50 to 0.45)	1.91 (1.82–1.99)	207.65 (203.51–211.82)	0.17 (0.18 to 0.17)	322.40 (309.51–336.12)	0.25 (0.26 to 0.24)	1.25 (1.07–1.43)	12044.94 (11827.71–12263.41)	10.03 (10.33 to 9.75)	18916.12 (18164.72–19723.57)	14.69 (15.45 to 13.95)	1.28 (1.11–1.46)
Southern Latin America	10.28 (8.69–12.07)	0.06 (0.07 to 0.05)	29.60 (25.88–33.37)	0.11 (0.13 to 0.09)	2.68 (2.47–2.89)	9.13 (7.71–10.71)	0.05 (0.06 to 0.04)	24.38 (21.32–27.51)	0.09 (0.11 to 0.08)	2.48 (2.25–2.71)	528.68 (447.54–619.66)	2.86 (3.52 to 2.31)	1421.72 (1243.90–1608.38)	5.35 (6.42 to 4.44)	2.52 (2.29–2.75)
Andean Latin America	52.47 (44.16–64.37)	0.37 (0.49 to 0.29)	77.48 (59.29–101.22)	0.29 (0.41 to 0.20)	−1.21 (−1.58 to −0.82)	68.70 (52.65–89.05)	0.35 (0.45 to 0.27)	48.95 (41.41–59.97)	0.25 (0.36 to 0.18)	−1.44 (−1.82 to −1.05)	2971.34 (2524.14–3615.47)	20.70 (26.94 to 16.11)	4130.71 (3179.67–5320.29)	15.28 (21.57 to 10.78)	−1.41 (−1.79 to −1.03)
Tropical Latin America	111.37 (106.72–116.20)	0.19 (0.20 to 0.17)	151.47 (143.32–159.77)	0.16 (0.17 to 0.15)	0.00 (−0.24–0.24)	102.22 (98.09–106.41)	0.17 (0.18 to 0.16)	134.06 (126.97–141.40)	0.14 (0.15 to 0.13)	−0.01 (−0.24–0.23)	6051.52 (5809.91–6302.58)	10.04 (10.79 to 9.36)	7868.07 (7467.28–8280.99)	8.51 (9.16 to 7.90)	0.03 (−0.20–0.26)
Central Latin America	133.49 (129.75–138.05)	0.22 (0.23 to 0.21)	227.74 (204.73–251.33)	0.23 (0.25 to 0.20)	0.13 (−0.18–0.44)	123.02 (119.68–127.15)	0.20 (0.21 to 0.19)	201.70 (180.72–222.92)	0.20 (0.22 to 0.18)	−0.03 (−0.41–0.35)	7365.74 (7163.44–7598.87)	11.91 (12.60 to 11.27)	11999.28 (10770.79–13268.98)	11.88 (13.23 to 10.56)	0.01 (−0.36–0.39)
Caribbean	27.70 (24.27–32.00)	0.21 (0.25 to 0.17)	34.51 (27.45–42.37)	0.19 (0.24 to 0.14)	−0.70 (−1.04 to −0.36)	25.07 (21.96–29.00)	0.19 (0.23 to 0.16)	30.36 (23.83–37.50)	0.16 (0.22 to 0.13)	−0.72 (−1.07 to −0.36)	1482.57 (1303.17–1711.45)	10.89 (13.13 to 9.14)	1772.06 (1396.08–2182.12)	9.63 (12.60 to 7.37)	−0.72 (−1.07 to −0.37)
North Africa and Middle East	466.48 (381.63–600.43)	0.40 (0.53 to 0.31)	1041.47 (885.34–1212.00)	0.39 (0.48 to 0.32)	−0.07 (−0.26–0.11)	429.25 (351.47–553.87)	0.36 (0.49 to 0.28)	907.53 (769.55–1055.12)	0.34 (0.42 to 0.28)	−0.23 (−0.42 to −0.03)	25535.86 (20995.27–33151.23)	21.32 (28.59 to 16.53)	53268.30 (45089.77–61982.60)	20.33 (24.91 to 16.55)	−0.18 (−0.37–0.02)
Southern Sub-Saharan Africa	255.17 (164.58–385.93)	1.39 (2.11 to 0.87)	522.57 (416.86–649.06)	1.50 (1.93 to 1.16)	−0.46 (−1.51–0.61)	233.23 (150.49–354.12)	1.26 (1.92 to 0.79)	471.32 (377.29–587.72)	1.35 (1.75 to 1.04)	−0.54 (−1.64–0.58)	13587.67 (8804.29–20687.66)	72.59 (110.27 to 45.83)	27142.18 (21720.77–33787.56)	78.06 (100.72 to 60.20)	−0.53 (−1.64–0.58)
Western Sub-Saharan Africa	1054.04 (671.43–1584.92)	1.72 (2.65 to 1.07)	1054.04 (671.43–1584.92)	1.34 (1.74 to 1.00)	−1.00 (−1.12 to −0.89)	977.81 (623.31–1469.47)	1.58 (2.44 to 0.98)	2014.27 (1543.64–2534.55)	1.23 (1.59 to 0.92)	−1.01 (−1.14 to −0.89)	57944.91 (36916.98–87041.82)	92.23 (142.32 to 57.35)	119383.36 (91557.87–150479.50)	71.51 (92.94 to 53.41)	−1.00 (−1.13 to −0.88)
Central Sub-Saharan Africa	132.17 (58.00–307.06)	0.75 (1.77 to 0.31)	244.55 (106.48–580.31)	0.52 (1.24 to 0.21)	−1.38 (−1.49 to −1.28)	123.08 (53.82–284.53)	0.69 (1.61 to 0.29)	226.73 (97.83–560.60)	0.48 (1.16 to 0.20)	−1.37 (−1.47 to −1.27)	7304.81 (3204.28–17006.01)	40.12 (93.81 to 16.64)	13428.07 (5825.85–33126.97)	27.94 (67.55 to 11.46)	−1.37 (−1.47 to −1.27)
Eastern Sub-Saharan Africa	376.13 (301.62–487.88)	0.62 (0.83 to 0.48)	870.94 (630.92–1218.90)	0.58 (0.82 to 0.41)	−0.62 (−0.84 to −0.39)	351.48 (282.10–452.94)	0.58 (0.77 to 0.45)	806.47 (581.95–1141.00)	0.53 (0.76 to 0.37)	−0.61 (−0.84 to −0.38)	21112.70 (16980.45–27186.23)	33.92 (44.97 to 26.31)	48233.65 (34939.46–67793.32)	31.04 (44.30 to 21.93)	−0.62 (−0.85 to −0.39)
**SDI regions**															
High SDI	1911.08 (1723.54–2146.93)	0.52 (0.62 to 0.46)	1911.47 (1805.61–2035.15)	0.48 (0.52 to 0.45)	−0.57 (−0.72 to −0.42)	1499.03 (1343.24–1694.78)	0.41 (0.49 to 0.35)	1122.97 (1060.27–1191.16)	0.28 (0.31 to 0.26)	−1.56 (−1.71 to −1.42)	85914.87 (77148.36–96717.96)	23.58 (28.22 to 20.34)	64530.06 (61004.47–68416.54)	16.45 (17.78 to 15.30)	−1.52 (−1.66 to −1.38)
Low SDI	1250.29 (885.05–1780.80)	0.78 (1.11 to 0.54)	2719.06 (2136.52–3635.80)	0.69 (0.95 to 0.54)	−0.63 (−0.75 to −0.50)	1162.82 (823.09–1648.82)	0.72 (1.03 to 0.51)	2512.13 (1963.58–3374.09)	0.64 (0.87 to 0.49)	−0.62 (−0.75 to −0.49)	69157.12 (49044.62–98003.32)	42.06 (60.10 to 29.56)	149570.64 (116850.33–201321.11)	37.34 (50.97 to 28.92)	−0.62 (−0.75 to −0.49)
Middle SDI	9266.33 (8084.58–10766.85)	1.39 (1.66 to 1.17)	10484.52 (8891.99–12866.82)	1.05 (1.31 to 0.87)	−1.42 (−1.66 to −1.18)	8381.64 (7309.34–9702.61)	1.25 (1.49 to 1.06)	8184.62 (6945.89–9954.80)	0.82 (1.01 to 0.68)	−1.83 (−2.09 to −1.57)	484952.77 (423777.80–561705.36)	71.84 (85.40 to 60.63)	466234.38 (396473.58–565462.25)	46.94 (57.86 to 39.13)	−1.85 (−2.11 to −1.58)
High-middle SDI	5532.12 (4657.17–6689.46)	1.24 (1.51 to 1.02)	5386.91 (4295.19–6784.88)	1.02 (1.30 to 0.80)	−1.63 (−2.04 to −1.22)	4941.57 (4151.36–6003.76)	1.10 (1.35 to 0.91)	3960.25 (3172.53–4987.75)	0.75 (0.96 to 0.59)	−2.26 (−2.72 to −1.81)	283391.12 (238651.23–344123.32)	63.28 (77.52 to 52.20)	223568.44 (179417.14–280346.12)	42.94 (54.44 to 33.65)	−2.28 (−2.73 to −1.83)
Low-middle SDI	1896.75 (1614.40–2417.00)	0.47 (0.61 to 0.39)	3837.57 (3290.94–4538.98)	0.50 (0.60 to 0.42)	−0.03 (−0.18–0.11)	1756.61 (1495.88–2232.82)	0.43 (0.56 to 0.36)	3482.90 (2979.80–4135.12)	0.45 (0.54 to 0.38)	−0.07 (−0.22–0.08)	103952.90 (88595.20–131590.53)	25.32 (32.45 to 21.03)	204337.76 (174406.30–243046.21)	26.35 (31.82 to 22.00)	−0.07 (−0.22–0.08)
**Etiology**															
Hepatitis B	15203.85 (12864.88–18114.02)	0.75 (0.63 to 0.89)	17639.05 (14667.87–21498.93)	0.57 (0.48 to 0.70)	−1.49 (−1.75–−1.23)		0.67 (0.57 to 0.80)	13881.11 (11545.80–16901.87)	0.45 (0.38 to 0.55)	−1.88 (−2.17–−1.59)	788132.47 (667008.23–941611.32)	38.38 (32.49 to 45.85)	795978.04 (662677.09–968538.11)	26.01 (21.65 to 31.63)	−1.88 (−2.17–−1.59)
Hepatitis C	1013.39 (677.39–1442.94)	0.05 (0.03 to 0.07)	1405.44 (923.2–2070.88)	0.05 (0.03 to 0.07)	−0.59 (−0.73–−0.46)		0.04 (0.03 to 0.06)	1114.65 (719.83–1652.59)	0.04 (0.02 to 0.05)	−0.85 (−0.99–−0.70)	49162.17 (32522.26–70878.35)	2.44 (1.62 to 3.51)	62626.86 (40326.18–93173.50)	2.04 (1.31 to 3.03)	−0.84 (−0.98–−0.69)
Alcohol use	1199.84 (769.87–1772.54)	0.06 (0.04 to 0.09)	1906.93 (1241.41–2814.69)	0.06 (0.04 to 0.09)	−0.28 (−0.42 to −0.14)		0.05 (0.03 to 0.08)	1496.58 (967.73–2233.87)	0.05 (0.03 to 0.07)	−0.56 (−0.70–−0.41)	57900.31 (36891.84–86294.10)	2.88 (1.84 to 4.30)	83774.62 (54163.78–125243.45)	2.72 (1.76 to 4.07)	−0.55 (−0.70–−0.40)
NASH	926.18 (641.96–1290.45)	0.04 (0.03 to 0.06)	1592.51 (1097.35–2231.68)	0.05 (0.04 to 0.07)	0.17 (0.03–0.32)		0.04 (0.03 to 0.06)	1336.40 (915.31–1888.93)	0.04 (0.03 to 0.06)	−0.04 (−0.21–0.12)	50397.60 (34963.88–70295.36)	2.39 (1.65 to 3.33)	79946.53 (54842.36–112959.87)	2.65 (1.82 to 3.75)	−0.01 (−0.18–0.15)
Other causes	1520.40 (1067.17–2022.44)	0.07 (0.05 to 0.10)	1804.13 (1244.86–2453.70)	0.06 (0.04 to 0.08)	−1.14 (−1.34 to −0.94)		0.07 (0.05 to 0.09)	1441.48 (984.82–1980.04)	0.05 (0.03 to 0.07)	−1.49 (−1.72–−1.26)	82153.40 (57802.00–109523.78)	3.89 (2.73 to 5.18)	86343.87 (59073.38–118553.44)	2.86 (1.96 to 3.92)	−1.46 (−1.69–−1.23)

#### Mortality

Based on the GBD 2021 estimates, there were 19,270 (95% UI 17036.81 to 22393.10) AYA individuals globally who died due to LC, with East Asia (8,884) reporting the largest number of fatalities. In terms of mortality, the global ASR was 0.63 per 100,000 population (Table 1). Throughout 204 countries, the top five age-standardized mortality rate were recorded in Gambia (ASR, 4.83), Mongolia (ASR, 4.24), Eswatini (ASR, 3.77), Guinea-Bissau (ASR, 3.38) and Mali (ASR, 3.18; Supplementary Table S3 and Figure 1B). From the perspective of GBD regions, East Asia experienced the highest age-standardized mortality rate (ASR, 1.53), followed by Southern Sub-Saharan Africa (ASR, 1.35) and Western Sub-Saharan Africa (ASR, 1.23). In terms of sex, males (ASR, 0.93) had about 2.9 times the worldwide mortality as females (ASR, 0.32; Supplementary Table S4).

#### DALYs

An estimated 1,108,669 (95% UI 980,707.98 to 1,285,863.91) years of disability-adjusted life years (DALYs) were lost globally due to LC among AYA, with East Asia accounting for the highest number of lost years (501,526; Table 1). The global average ASR was 36.28 per 100,000 population, and the top five countries with the highest age-standardized DALY rate were Gambia (ASR, 280.72), Mongolia (ASR, 247.34), Eswatini (ASR, 215.46), Guinea-Bissau (ASR, 197.11) and Mali (ASR, 184.99; Supplementary Table S5 and Figure 1C). At the regional level, East Asia had the highest age-standardized DALY rate (ASR, 87.00), followed by Southern Sub-Saharan Africa (ASR, 78.06) and Western Sub-Saharan Africa (ASR, 71.51; Table 1). The DALYs of males (ASR, 53.21) were 2.6 times those of females (ASR, 18.99), and similar to incidence and mortality rates, males in East Asia had the highest DALYs among all subgroups of different sex (Supplementary Table S6).

### Trends in incidence, mortality, and DALYs

#### Incidence

From 1990 to 2021, the global age-standardized incidence rate of LC among AYA exhibited a decreasing trend, with an average change of −1.25 (95% CI −1.48 to −1.01) per year, as shown in Table 1. At the national level, nearly 63.05% of the 203 countries reported a declining incidence, but significant increases were still observed in some countries like United Republic of Tanzania (EAPC, 5.15), Liberia (EAPC, 4.50) and Guatemala (EAPC, 4.10; Supplementary Table S1 and Figure 1D). Across the 21 GBD regions, 13 regions had a decreasing incidence, among these High-income Asia Pacific (EAPC, −2.88), Central Europe (EAPC, −1.72), and Central Asia (EAPC, −1.06) showed the fastest-decreasing trend. In contrast, the incidence of the remaining regions experienced an increase with varying degrees, reaching the highest increase in Southern Latin America (EAPC, 2.68). Additionally, Australasia (EAPC, 2.62), High-income North America (EAPC, 2.62) and Western Europe (EAPC, 1.39) also exhibited a high level of increasing trend in incidence (Table 1).

#### Mortality

Between 1990 and 2021, the global mortality trend for LC among AYA decreased (EAPC, −1.60). The most significant decreases were observed in Kyrgyzstan (EAPC, −5.07), Republic of Moldova (EAPC, −4.66) and Iceland (EAPC, −4.29). However, high level of upward trends were also recorded in some countries, including Liberia (EAPC, 4.55) and United Republic of Tanzania (EAPC, 4.12; Supplementary Table S3 and Figure 1E). At the regional level, High-income Asia Pacific had the greatest decreasing trend (EAPC, −4.37), followed by Central Europe (EAPC, −1.81) and East Asia (EAPC, −1.76). Increasing trends of mortality were observed in six regions, reaching the highest increases in Southern Latin America (EAPC, 2.48) and Australasia (EAPC, 1.74; Table 1).

#### DALYs

From 1990 to 2021, the global age-standardized DALY rate decreased with an EAPC of −1.60, with similar trends in females (EAPC, −1.41) and males (EAPC, −1.65; Table 1). The top five nations with the highest trend of decreasing DALYs included Kyrgyzstan (EAPC, −5.04), Slovakia (EAPC, −4.80), Republic of Moldova (EAPC, −4.67), Iceland (EAPC, −4.20) and Lao People's Democratic Republic (EAPC, −4.02), whereas the top five countries with the highest upward trend Liberia (EAPC, 4.49), United Republic of Tanzania (EAPC, 4.09), Guatemala (EAPC, 3.90), Eswatini (EAPC, 3.83), and Uzbekistan (EAPC, 3.40; Supplementary Table S5 and Figure 1F). At the regional level, the fastest-increasing trend of DALYs due to LC among AYA was found in the Southern Latin America (EAPC, 2.52), whereas High-income Asia Pacific (EAPC, −4.34) showed the fastest-declining trend.

### Age-group disparities in the burden of LC among AYA

**Figure 2:**
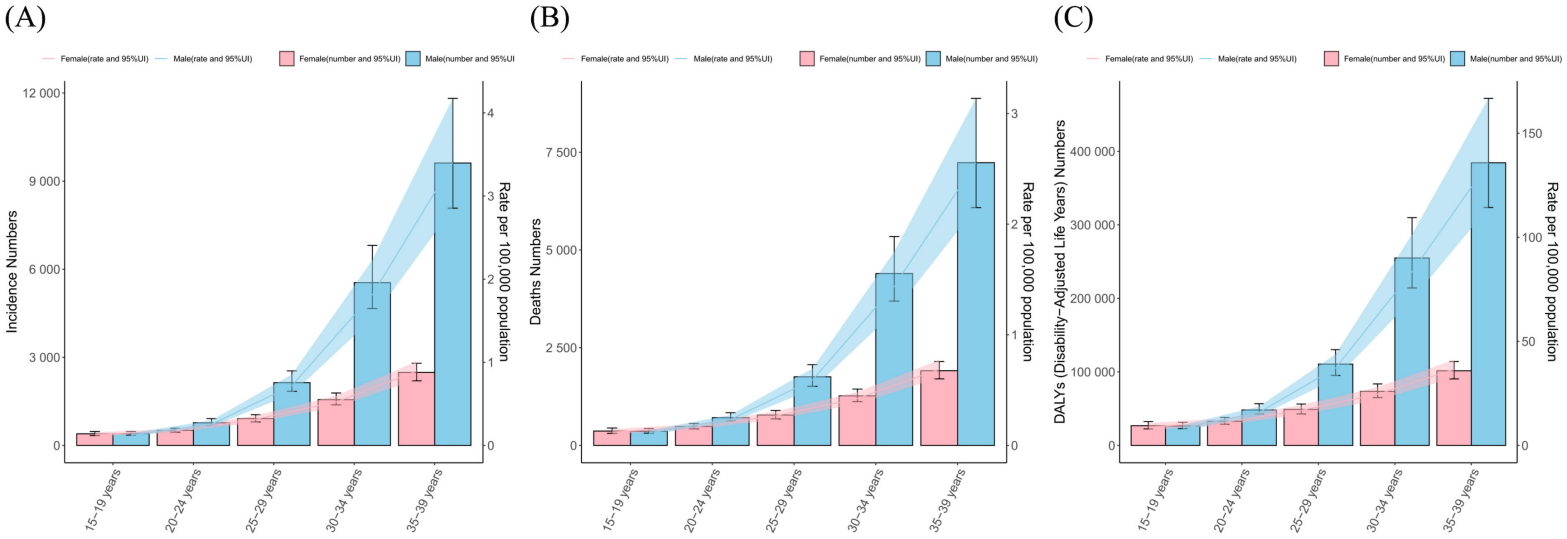
The numbers and age-standalized rates of **(A)** incidence, **(B)** mortality and **(C)** DALYs of liver cancer among AYA, by sex and age groups.

Among AYA, the incidence, mortality and DALYs number and rates of LC increased with age and reached the highest at age 35–39 (Figure 2). In the 15–19 age group, the incidence, mortality and DALYs rates of females were slightly higher than those of males. However, the rate of males exceeded that of females, and this disparity progressively widens with increasing age. In the 35–39 age group, males exhibit significantly higher rates of incidence, mortality, and DALYs, standing at 3.8, 3.7, and 3.7 times higher than their female counterparts, respectively.

### The association between ASR, EAPC, and SDI

**Figure 3:**
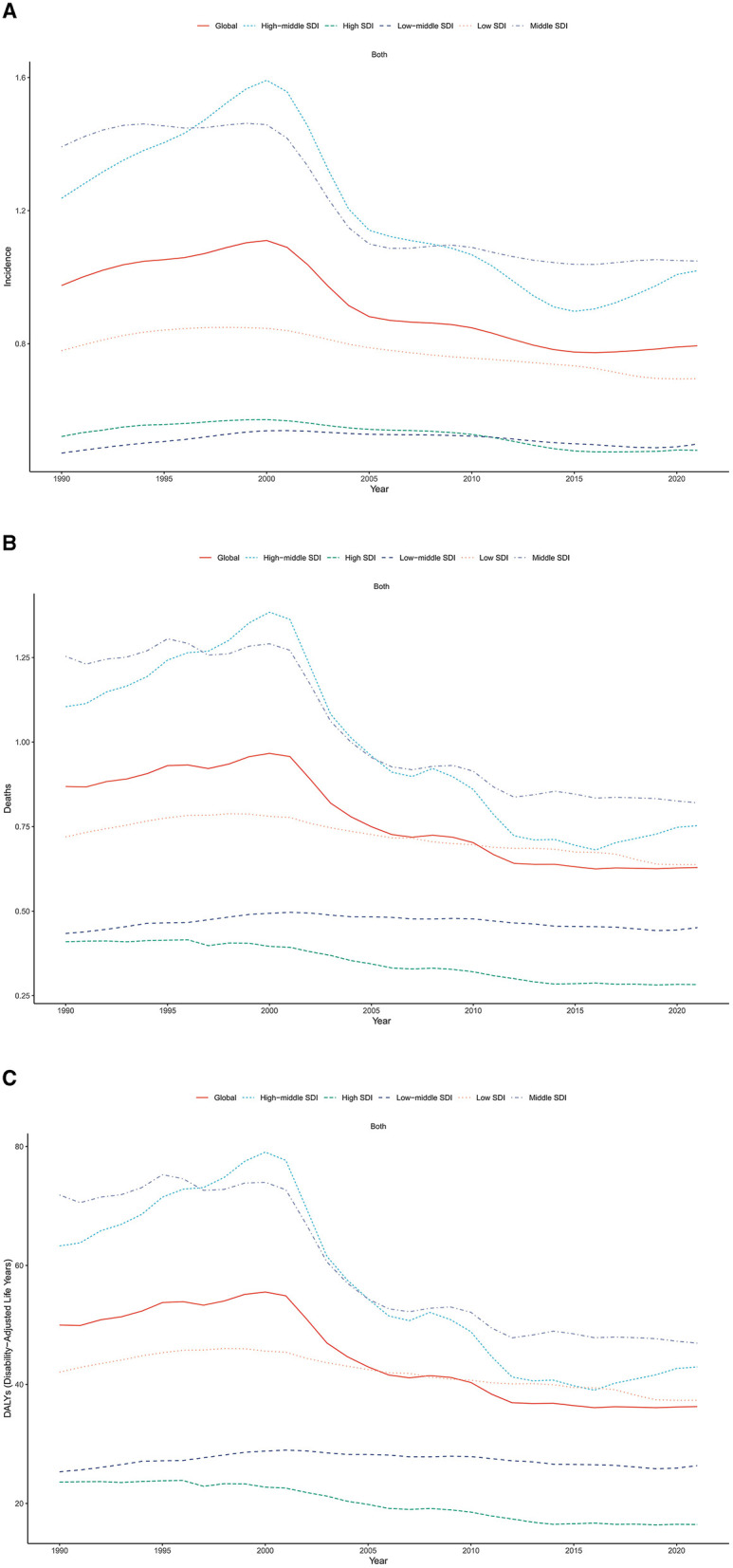
Trends of age-standardized **(A)** incidence, **(B)** mortality and **(C)** DALY rates of liver cancer among AYA, by SDI, 1990–2021.

In 2021, Middle SDI countries had the largest frequency of incident cases, mortality and DALYs due to LC in AYA, accounting for 43.1, 42.5, and 44.7% of the global numbers. Regarding ASR, the highest rates of incidence, mortality and DALYs were observed in Middle SDI countries (ASR, 1.05, 0.82, 46.94) and High-middle SDI countries (ASR, 1.02, 0.75, 42.94), while High SDI countries had the lowest rates (ASR, 0.48, 0.28, 16.45; Table 1). From 1990 to 2021, the trend of incidence, mortality and DALYs were observed to be declining in all SDI regions, in which High-middle SDI countries had the greatest decreasing trend (Figure 3).

**Figure 4:**
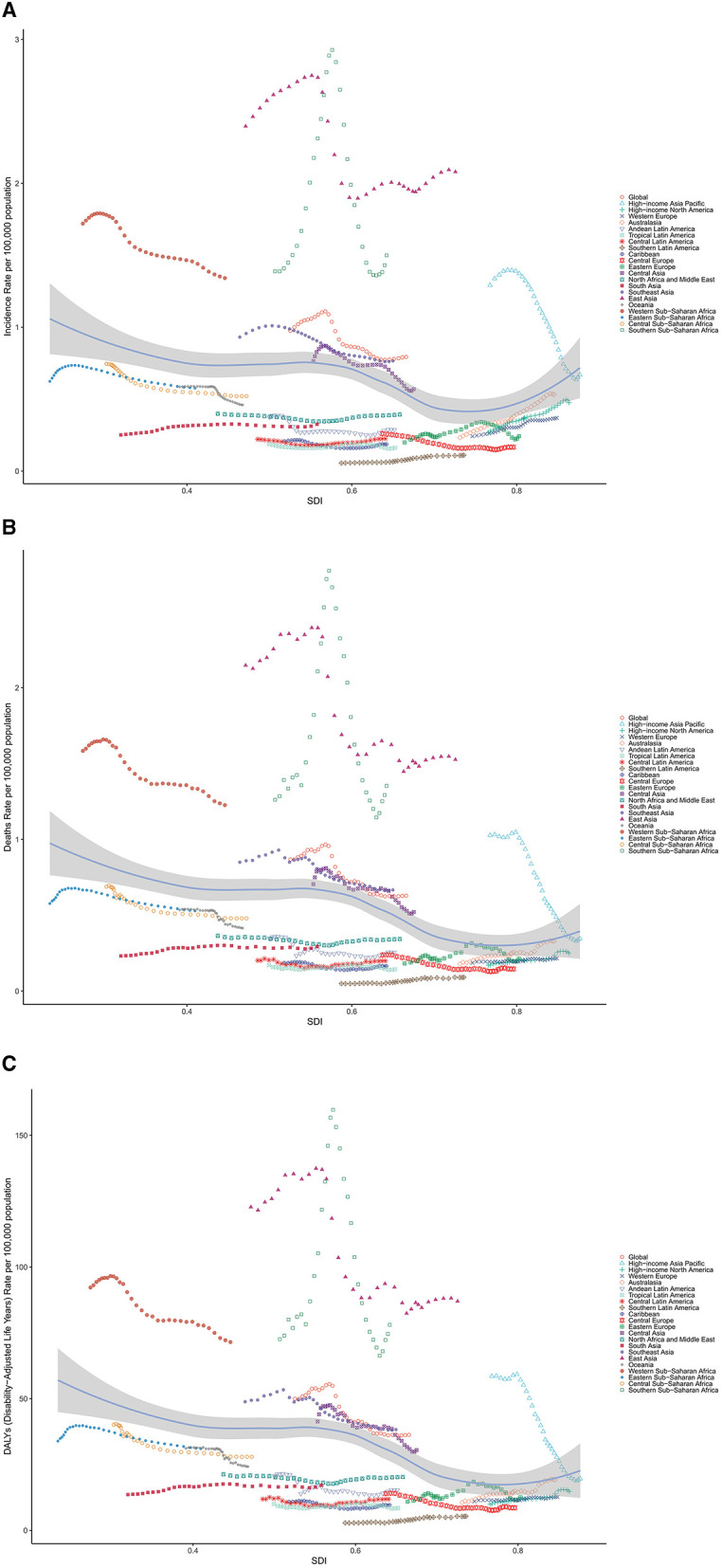
Age-standardized **(A)** incidence, **(B)** mortality and **(C)** DALY rates of liver cancer among AYA, globally and for 21 GBD regions, by SDI, 1990–2021. (Expected values, based on SDI and disease rates in all locations, are shown as a solid line; expected values based on a calculation accounting for the SDI and disease rates across all locations. Thirty-two points are plotted for each region and show the observed age-standardized rates for each year from 1990 to 2021 for that region. The shaded area indicates the 95% CI of the expected values. Points above the solid line represent a higher-than-expected burden, and those below the line show a lower-than-expected burden).

In the analysis between ASRs and SDI, we compared the ASRs of GBD regions across different SDIs. In 2021, the incidence ASR of LC among AYA decreased with rising SDI. East Asia, Southern Sub-Saharan Africa, Western Sub-Saharan Africa, Central Sub-Saharan Africa, High-income Asia Pacific, Southeast Asia and Central Asia had higher-than expected age-standardized incidence rates based on their SDI, between 1990 and 2021. Similar patterns were found for mortality and DALYs in relation to SDI, as shown in Figure 4.

### The etiology

**Figure 5:**
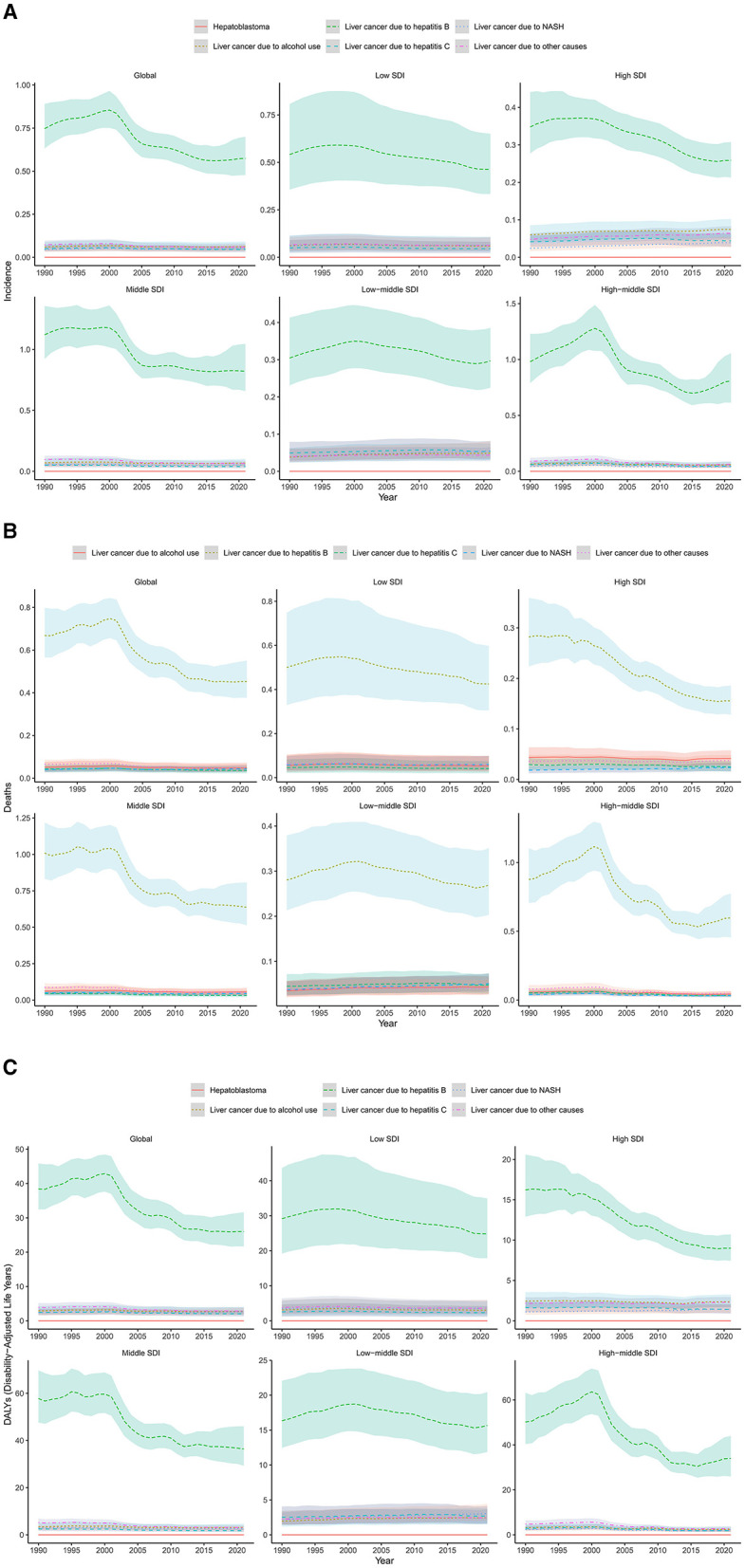
Trends of liver cancer in AYA in different age groups over time from 1990 to 2021 **(A)** incidence; **(B)** mortality; **(C)** DALYs.

In 2021, HBV accounted for the highest incidences (*n* = 17,639), mortality (*n* = 13,881), and DALYs (*n* = 795,978) related to LC. Similarly, LC from HBV had the highest ASIR (0.57, 95% UI 0.48 to 0.79), ASDR (0.45, 95% UI 0.38 to 0.55) and ASDALYs (26.01, 95% UI 21.65 to 31.63) per 100,000 population among LC in AYA. Among five etiologies of LC listed in the 2021 GBD study, HBV (72.4%) accounted for the largest proportion of LC incidences, followed by alcohol use (7.8%), other causes (7.4%), NASH (6.5%) and HCV (5.8%). From 1990 to 2021, age-standardized incidence rate attributable to LC among AYA decreased in HBV (EAPC, −1.49), other cause (EAPC, −1.14) and HCV (EAPC, −0.59), remain stable in alcohol use, and increased in NASH (EAPC, 0.17). NASH was the fastest-growing etiology of incident LC cases globally, and reached the highest EAPC in High SDI regions (EAPC, 1.15; Table 1 and Figure 5A).

When considering mortality, in 2021, ~72% of LC related mortality in AYA were attributed to HBV, 7.8% were attributed to alcohol use, 7.4% were attributed to other causes, 6.5% were attributed to NASH, and 5.8% were attributed to hepatitis C. Although remaining the leading cause of LC related mortality, the age-standardized mortality rate of LC due to HBV show a significant decreasing trend by 32.3% from 0.67 (95% UI 0.57–0.80) in 1990 to 0.45 (95% UI 0.38–0.55) in 2021. In High SDI regions, the decrease even reached 44.8%, while in low-middle and low SDI regions, the age-standardized mortality rate of LC due to HBV decreased more slightly (Table 1 and Figure 5B).

Similarly, from 1990 to 2021, age-standardized DALY rate from HBV, HCV and other cause remained stable in alcohol use and exhibited an uptrend only for NHSA. Variations in etiologies according to the SDI classification were detailed in Table 1 and Figure 5C.

### Prediction of future disease burden trends

We used the BAPC model to predict the disease burden of LC among AYA from 2021 to 2040. The global LC disease burden among AYA will continue to decline, with the number of all-age cases dropping to 22,274.45 (95% CI: 14,819.36–29,729.54) by 2040, and ASDR dropping to 0.68 (95% CI: 0.46–0.92; Supplementary Table S7). Compared to 2021, the number of AYA cases will decrease by 5.32%, and the ASDR will decrease by 12.94%. Specifically, by sex, the number of AYA cases in males will decrease by 17.58%, and ASIR will decrease by 25.26%. However, the number of cases in females will increase by 11.32%, and ASDR by 3.9%. The proportion of female cases will increase from 24.15 to 30.07%. The predictions suggest that while the global LC burden in AYA will continue to decline, the number of cases in females is showing an increasing trend.

## Discussion

This study spans a period of 30 years and predicts the disease burden up to the year 2040. It comprehensively estimates the incidence, mortality, and DALYs among AYAs across five SDI regions, 21 GBD regions, and 204 countries and territories from 1990 to 2021. The primary findings are as follows: First, the global ASRs of incidence, mortality, and DALYs for LC among AYA have decreased from 1990 to 2021. Second, among GBD regions, East Asia has the highest ASRs of incidence, mortality, and DALYs for LC. Third, male AYA show significantly higher rates of incidence, mortality, and DALYs, at 3.1, 2.9, and 2.6 times those of their female counterparts, respectively. Fourth, among AYA, the incidence, mortality and DALYs number and rates of LC increased with age and reached the highest at age 35–39. These results are in line with previous studies (22). Fifth, in 2021, the age-standardized incidence rate of LC among AYA decreased with rising SDI, and similar patterns were found for mortality and DALYs in relation to SDI. Sixth, although HBV remains dominant in LC related incidences, mortality and DALYs, NASH was the fastest growing etiology of incident LC cases.

Our research shows that in 2021, there were 24,348 new cases of LC and 19,270 mortality among AYA, with LC causing in 1,108,669 DALYs globally. This finding aligns with previous studies (22), indicating the broader social and economic impact of the disease on AYAs. While LC is usually seen as a disease affecting middle-aged and older individuals, it also significantly burdens the AYA group. The global rise of obesity among AYA is likely contributing to this rise. The core mechanism of obesity-driven NASH progressing to LC involves metabolic imbalance, inflammatory fibrosis, and genetic abnormality (23, 24). Excess fatty acids from visceral fat overload the liver, causing lipid accumulation and oxidative stress (25). Inflammatory factors from adipose tissue (TNF-α, IL-6) disrupt insulin signaling, exacerbating lipid synthesis and insulin resistance (26). In addition, the stress associated with obesity also fuels genomic instability and carcinogenesis through telomere depletion and epigenetic changes (27). This multilevel mechanism reveals a continuous pathological process from metabolic disorders to LC. Dietary habits and physical activity vary across regions, affecting NASH prevalence. In North and South Latin America, consume the most sugar-sweetened beverages, exacerbating NASH through fructose and microbiota disruptions (28). The Middle East and North Africa have a high-fructose, refined-carb diet and fast food, creating metabolic burdens (29). In addition to a high-carbohydrate diet in areas with high AFB1 exposure, hepatocyte proliferation and carcinogenesis can be promoted by activating the mTOR pathway (30). In East Asian, AYAs face prolonged sitting and sarcopenia, worsening insulin resistance (31). In the Nordic countries, policy-driven “national campaigns” significantly reduced liver fat content (32, 33). Therefore, prevention strategies should take into account regional differences in diet, activity levels, and policies, including measures like taxing high-sugar diets and promoting exercise among sedentary populations.

Our analysis of the regional aspects of LC in AYA further indicates that East Asia is the most severely affected region in the world. The incidence, mortality and DALYs rates of LC among AYA in East Asia are the highest, with the ASR more than double that of the global ASR, posing a significant threat to the AYA in this region. Notably the most striking countries are Gambia, Mongolia, Eswatini, Guinea-Bissau, and Mali. The high burden of LC in low- and middle-income countries (LMICs) is closely related to socioeconomic factors and is also jointly influenced by the SDI, life expectancy, and healthcare systems (34). The increase in alcohol consumption driven by economic transition has significantly exacerbated the disease burden (35). In LMICs, disparities in healthcare systems, encompassing preventive strategies, screening coverage, and access to treatment, can significantly impact the prognosis for low-income patients (36, 37). For instance, 80% of patients with LC in sub-Saharan Africa are diagnosed with advanced stage (stage III/IV), and the 5-year survival rate is < 5%. In LMICs where inadequate healthcare systems contribute to poor prognoses for LC, priority should be given to addressing three major bottlenecks: the lack of early screening, barriers to treatment costs, and shortages of health human resources.

It is also crucial to comprehend the trends in LC among AYAs over the past three decades and its foreseeable future burden. From 1990 to 2021, and with projections extending to 2040, a declining trend is observed in the age-standardized incidence rate, mortality rate, and DALYs due to LC among AYAs. This suggests that global public health efforts to reduce the burden of LC have been somewhat successful, especially in high-income Asia Pacific regions. This trend may be attributed to the widespread adoption of early screening, vaccination (especially against HBV), antiviral treatments, and effective health education. In contrast, Southern Latin America has the fastest rising trend in LC. South Latin America has seen rapid obesity growth, leading to rapid progression from NAFLD to LC (38, 39). This stems from viral hepatitis, alcohol abuse, healthcare disparities, toxins, and poorer surveillance (40). Comprehensive strategies are needed, such as HBV vaccines, alcohol taxation tools, strengthening LC diagnosis and data systems at the grassroots level, and promoting multi-sectoral policy synergies to improve the built environment. These will tackle non-communicable diseases, but will also promote universal health coverage and help meet the UN Sustainable Development Goal of reducing premature mortality from non-communicable diseases by one third by 2030 (41).

This regional imbalance reflects differences in the effectiveness of global LC management, influenced by factors such as accessibility to health services, policy support, and economic conditions. Our trends are consistent with the 2019 GBD data study (42). This result also indicates that the global COVID-19 pandemic has not caused an increase in the burden of LC in AYAs (43). COVID-19 mainly affects middle-aged and older adult who have weaker immunity, while its impact on AYAs with LC is less evident. The pandemic poses unprecedented challenges to global healthcare, severely impacting LC diagnosis, treatment, and management in resource-limited areas, with 50% of centers halting LC treatments (44). Global LC screening coverage has declined, resulting in early cases being missed. LC development from hepatitis or fatty liver takes 10–30 years, so current monitoring may miss outbreak risks, and the impacts of an HCV surge won't be clear until after 2030 (45).

However, in high-income regions like Australasia, North America, and Western Europe, incidence rates have increased, potentially due to several contributing factors. High-income countries may face the transmission of HBV and HCV associated with drug use, sexual transmission, and untested blood products, with hepatitis viruses being one of the main triggers for LC (46, 47). Moreover, young people in these regions may have experienced significant lifestyle changes, including altered dietary habits, lack of exercise, and rising obesity rates (48–50). Immigration may bring health risks from different regions, and the lifestyle of young people may be influenced, especially if they come from countries or regions with a high burden of LC (51–53). As the population ages and liver diseases increase, such as alcoholic liver disease and metabolic liver disease (like non-alcoholic fatty liver disease), these conditions may lead to an increased risk of LC among AYAs. Although healthcare systems in developed countries are relatively strong, issues such as uneven distribution of medical resources and insufficient focus on specific health problems may still exist. This may lead to some young people not receiving timely liver disease screening and treatment, thus affecting their health status.

In 2021, countries with a middle SDI accounted for over 40% of global cases, mortality, and DALYs among AYAs, highlighting significant challenges in managing this disease. In contrast, high-income countries have a relatively low ASR of LC, suggesting that their healthcare systems and health policies for screening, diagnosis, and treatment are more advanced. Countries such as Australia have successfully incorporated HBV into their childhood immunization schedules, resulting in a reduction in HBV infection rates and subsequent LC incidence (54). Monitoring of high-risk groups in Italy has improved survival rates (55). Key factors that make these regions more successful in implementing these initiatives include access to health care, public awareness, and a robust health care infrastructure.

At the same time, it has been observed that in middle and high SDI countries, the incidence, mortality, and DALYs of LC have declined from 1990 to 2021, showing positive progress in public health interventions and medical technology development (56). Despite the overall positive trend, it is crucial to focus on the persistently high incidence of LC in certain high-risk areas. The research results also indicate that the incidence of LC in East Asia, sub-Saharan Africa, and certain high-income Asia Pacific regions is higher than expected. These regions may face specific health risks, including high transmission rates of hepatitis viruses and insufficient vaccination coverage and screening measures. Enhancing the capacity for LC management in these regions will be an important public health challenge (57). Additionally, there must be a strengthened focus on the prevention and management of liver disease in LMICs countries to narrow the regional gap.

The issue of gender disparities in LC among AYA is receiving increasing attention. Different genders may encounter varied risk factors for LC. Prior studies have established that the burden of LC in males significantly surpasses that in females globally (58). This study corroborates such findings, suggesting that AYA's males are not only more susceptible to LC but also endure a greater disease progression burden. A cohort study of 5.24 million adults in UK has further confirmed the significant modulation of LC incidence by gender (59). Higher testosterone levels in male may increase the risk of persistent HBV/HCV infection by activating hepatic stellate cells, promoting fibrosis and suppressing the immune response (60, 61). In addition, differences in exposure to risk behaviors in males are closely related to the development of LC, the global alcohol consumption rate of males is more than twice that of female, and heavy drinking and smoking synergistically increase the risk of LC (62). At the same time, males are more likely to be exposed to aflatoxin (agriculture, storage industry) and chemical carcinogens (such as vinyl chloride), which superposes the basis of chronic liver disease and accelerates cancer (63). It is noteworthy that although there is a trend of increasing cases in females from 1990 to 2021 and for the foreseeable future. This is primarily attributed to females specific metabolic and hormonal factors, as well as sociocultural and medical inequalities. Females tend to rely more on high-sugar snacks to alleviate stress, and insufficient exercise time due to family care responsibilities exacerbates their metabolic burden. Furthermore, female in low-income countries have lower access to LC screening and treatment compared to males. In response to this trend, it is recommended to pay special attention to the risk of LC in female AYAs in order to prevent the continued widening of health inequalities.

Interestingly, in the 15–19 age group, females show a slightly higher disease burden than males. However, this trend changes in older age groups, especially in the 35–39 range, where males surpass females significantly in all measures. This reflects the age-specific gender distribution characteristics of LC. In adolescence, females may face certain risks of LC due to physiological and hormonal changes (64). The rapid rise in obesity rates among adolescent females has led to an accelerated transformation of NAFLD to NASH (65). An increased prevalence of PCOS in young females is also associated with insulin resistance and abnormal androgen levels, which indirectly increases the risk of LC (66). However, in some LMICs, where the coverage of mother-to-child blocking measures is insufficient, females who were infected with HBV in childhood may enter the stage of active hepatitis in adolescence due to broken immune tolerance (67). Due to socioeconomic pressures and behavioral changes, some adolescent girls from low-income families face the coexistence of “undernutrition and obesity” and the consuming cheap, high-sugar/high-fat foods, which increases and exacerbates liver damage (68). As people age, males gradually catch up to and eventually exceed females in risk levels due to lifestyle changes and greater exposure to health risks. This suggests the need to strengthen long-term monitoring and prevention across the entire AYAs, especially for high-risk gender groups, to enable timely detection and intervention.

The importance of NASH in the shifting etiology of LC is profound. NASH, linked to metabolic syndrome, is a key driver of LC. Global rises in obesity, diabetes, and metabolic syndrome have increased NASH-related LC. Changes in lifestyle, particularly due to high-calorie diets, lack of exercise, and sedentary behavior, have significantly increased the rates of obesity and type 2 diabetes (69). NASH progresses to cirrhosis and LC independently of viral or alcoholic liver diseases. NASH has become the most common type of chronic liver disease in developed countries, especially in regions with high obesity rates such as North America, Europe, and Asia (70, 71). Our research aligns with this observation that NASH has become one of the fastest-growing causes of cancer, particularly in high SDI regions. With the global economic growth and the changes in dietary habits and lifestyles, the burden of NASH is expected to increase further.

However, until 2021, HBV remained a major pathogenic factor for LC incidence and DALYs in the AYAs. This reaffirms that HBV is a significant global risk factor for LC. Despite 20–30 years of universal vaccination efforts, chronic HBV infection remains prevalent, particularly in LMICs (72, 73). South Africa reduced the prevalence of HBV infection in children to < 1% (74) through a combination of universal neonatal vaccination and mother-to-child blocking strategies, and East Asia (e.g., Taiwan Province) implemented hospital-based and community-based hepatocellular carcinoma screening and mass vaccination programs, resulting in lower HBV-associated liver cancer rates (75, 76). However, there have been some failures, such as uneven vaccine coverage and low coverage of antiviral drugs in some countries in Eastern Europe (77). And most LMICs lack long-term surveillance data to assess the dose-response relationship between treatment coverage and liver cancer incidence (78). As of 2021, HBV was responsible for up to 72% of liver cancer-related deaths, highlighting its ongoing significant impact on LC mortality. This result emphasizes the importance of prevention and control measures against HBV in reducing the burden of LC among AYAs. Although the HBV-related age-standardized mortality rate declines each year, this trend varies significantly across different SDI regions. In high SDI regions, the HBV-related mortality rate decreased by 44.8%, indicating more successful interventions. In contrast, the reduction in middle- and low-SDI regions is relatively small, suggesting that these areas still require more refined strategies for HBV management and LC prevention and control. This suggests a need to enhance awareness of metabolic liver diseases and implement public health measures. In summary, understanding the main pathogenic factors of LC and the trends over time is crucial for formulating public health policies. Policymakers should prioritize large-scale screening and vaccination programs for HBV while also focusing on the management of emerging LC causes such as NASH. Strengthening health education and improving basic medical facilities could further reduce the burden of LC among AYAs in the future.

Despite the advantages of this study, some limitations must be acknowledged. First, reliance on the 2021 GBD data may introduce biases in data collection and reporting. Although the GBD offers a systematic approach to estimating disease burden, variations in data quality and completeness across regions still exist, since not all regions have the same level of data availability or accuracy. Many LMICs may lack comprehensive health data systems or high-quality cause-of-death registries, leading to underreporting or misclassification of certain diseases, particularly in rural or marginalized areas, which may impact the accuracy of our findings. Second, this study focused solely on AYA, excluding other age groups that may also be significantly affected by LC. Future research should aim to include a broader age range to provide a more comprehensive perspective on LC epidemiology. Finally, the study did not delve into the specific causes leading to regional and gender differences in LC burden. Understanding the factors contributing to regional and gender differences in LC burden is essential for developing effective prevention and treatment strategies. Future research should aim to explore these aspects in more detail, possibly integrating genetic, environmental, and lifestyle factors for a more comprehensive understanding of AYA LC epidemiology.

In conclusion, LC in AYA poses a significant challenge to global public health. From 1990 to 2021, the global burden of LC in AYA has decreased, particularly for cases caused by hepatitis viruses. However, NASH is now the fastest-growing cause of LC cases worldwide, and regional differences persist. Healthcare providers should understand the significance of the changing causes of LC in the AYAs. Additionally, healthcare strategies for primary and secondary prevention should be customized to address the specific needs of AYAs, considering factors such as age, gender, and region.

## Data Availability

The raw data supporting the conclusions of this article will be made available by the authors, without undue reservation.

## References

[1] Bray F Laversanne M Sung H Ferlay J Siegel RL Soerjomataram I. Global cancer statistics 2022: GLOBOCAN estimates of incidence and mortality worldwide for 36 cancers in 185 countries. CA Cancer J Clin. (2024) 74:229–63. 10.3322/caac.21834 38572751

[2] Llovet JM Burroughs A Bruix J. Hepatocellular carcinoma. Lancet. (2003) 362:1907–17. 10.1016/S0140-6736(03)14964-1 14667750

[3] van Gaal JC Bastiaannet E Schaapveld M Otter R Kluin-Nelemans JC de Bont ES. Cancer in adolescents and young adults in north Netherlands (1989–2003): increased incidence, stable survival and high incidence of second primary tumours. Ann Oncol. (2009) 20:365–73. 10.1093/annonc/mdn588 18725392

[4] Chang PE Ong WC Lui HF Tan CK. Is the prognosis of young patients with hepatocellular carcinoma poorer than the prognosis of older patients? A comparative analysis of clinical characteristics, prognostic features, and survival outcome. J Gastroenterol. (2008) 43:881–8. 10.1007/s00535-008-2238-x 19012042

[5] Ren J Tong YM Cui RX Wang Z Li QL Liu W. Comparison of survival between adolescent and young adult vs older patients with hepatocellular carcinoma. World J Gastrointest Oncol. (2020) 12:1394–406. 10.4251/wjgo.v12.i12.1394 33362910 PMC7739151

[6] Global regional and and national burden of hepatitis B 1990–2019: 1990–2019: a systematic analysis for the global burden of disease study 2019. Lancet Gastroenterol Hepatol. (2022) 7:796–829. 10.1016/S2215-0366(21)00395-3 35738290 PMC9349325

[7] Singal AG Kanwal F Llovet JM. Global trends in hepatocellular carcinoma epidemiology: implications for screening, prevention and therapy. Nat Rev Clin Oncol. (2023) 20:864–84. 10.1038/s41571-023-00825-3 37884736

[8] Tan DJH Setiawan VW Ng CH Lim WH Muthiah MD Tan EX. Global burden of liver cancer in males and females: changing etiological basis and the growing contribution of NASH. Hepatology. (2023) 77:1150–63. 10.1002/hep.32758 36037274

[9] Gritti I Wan J Weeresekara V Vaz JM Tarantino G Bryde TH. DNAJB1-PRKACA fusion drives fibrolamellar liver cancer through impaired SIK signaling and CRTC2/p300-mediated transcriptional reprogramming. Cancer Discov. (2025) 15:382–400. 10.1158/2159-8290.c.7662959 39326063 PMC11803398

[10] The global burden of adolescent and young adult cancer in 2019: a systematic analysis for the Global Burden of Disease Study 2019. Lancet Oncol. (2022) 23:27–52. 10.1016/S1470-2045(21)00581-7 34871551 PMC8716339

[11] Wen YF Chen MX Yin G Lin R Zhong YJ Dong QQ. The global, regional, and national burden of cancer among adolescents and young adults in 204 countries and territories, 1990-2019: a population-based study. J Hematol Oncol. (2021) 14:89. 10.1186/s13045-021-01093-3 34108026 PMC8191013

[12] Burden Burden of disease scenarios for 204 countries and territories 2022–2050: 2022–2050: a forecasting analysis for the global burden of disease study 2021. Lancet. (2024) 403:2204–56. 10.1016/S0140-6736(24)00932-2 38762325 PMC11121021

[13] Liu Z Mao X Jin L Zhang T Chen X. Global burden of liver cancer and cirrhosis among children, adolescents, and young adults. Dig Liver Dis. (2020) 52:240–3. 10.1016/j.dld.2019.11.001 31791699

[14] Global incidence prevalence years lived with disability (YLDs) disability-adjusted life-years (DALYs) and healthy life expectancy (HALE) for 371 diseases and injuries in 204 countries and territories and 811 subnational locations 1990–2021: 1990–2021: a systematic analysis for the Global Burden of Disease Study 2021. Lancet. (2024) 403:2133–61. 10.1016/S0140-6736(24)00757-8 38642570 PMC11122111

[15] What should the age range be for AYA oncology? J Adolesc Young Adult Oncol. (2011) 1:3–10. 10.1089/jayao.2011.1505 26812562

[16] Li CK Dalvi R Yonemori K Ariffin H Lyu CJ Farid M. Care of adolescents and young adults with cancer in Asia: results of an ESMO/SIOPE/SIOP Asia survey. ESMO Open. (2019) 4:e000467. 10.1136/esmoopen-2018-000467 31231565 PMC6555609

[17] Saloustros E Stark DP Michailidou K Mountzios G Brugieres L Peccatori FA. The care of adolescents and young adults with cancer: results of the ESMO/SIOPE survey. ESMO Open. (2017) 2:e000252. 10.1136/esmoopen-2017-000252 29018578 PMC5604713

[18] Ferrari A Stark D Peccatori FA Fern L Laurence V Gaspar N. Adolescents and young adults (AYA) with cancer: a position paper from the AYA working group of the European society for medical oncology (ESMO) and the European society for paediatric oncology (SIOPE). ESMO Open. (2021) 6:100096. 10.1016/j.esmoop.2021.100096 33926710 PMC8103533

[19] Guo C Liu Z Lin C Fan H Zhang X Wang H. Global epidemiology of early-onset liver cancer attributable to specific aetiologies and risk factors from 2010 to 2019. J Glob Health. (2023) 13:04167. 10.7189/jogh.13.04167 38085217 PMC10715628

[20] Li C He WQ. The impact of universal hepatitis B vaccine on the trend of liver cancer from the global burden of disease study 2017. Liver Int. (2021) 41:1762–74. 10.1111/liv.14821 33590659

[21] Bai Z Han J An J Wang H Du X Yang Z. The global, regional, and national patterns of change in the burden of congenital birth defects, 1990–2021: an analysis of the global burden of disease study 2021 and forecast to 2040. EClinicalMedicine. (2024) 77:102873. 10.1016/j.eclinm.2024.102873 39416384 PMC11474384

[22] Danpanichkul P Aboona MB Sukphutanan B Kongarin S Duangsonk K Ng CH. Incidence of liver cancer in young adults according to the global burden of disease database 2019. Hepatology. (2024) 80:828–43. 10.1097/HEP.0000000000000872 38598364

[23] Yahoo N Dudek M Knolle P Heikenwälder M. Role of immune responses in the development of NAFLD-associated liver cancer and prospects for therapeutic modulation. J Hepatol. (2023) 79:538–51. 10.1016/j.jhep.2023.02.033 36893854

[24] Asakawa M Itoh M Suganami T Sakai T Kanai S Shirakawa I. Upregulation of cancer-associated gene expression in activated fibroblasts in a mouse model of non-alcoholic steatohepatitis. Sci Rep. (2019) 9:19601. 10.1038/s41598-019-56039-0 31862949 PMC6925281

[25] Sun X Li X Jia H Wang H Shui G Qin Y. Nuclear factor E2-related factor 2 mediates oxidative stress-induced lipid accumulation in adipocytes by increasing adipogenesis and decreasing lipolysis. Antioxid Redox Signal. (2020) 32:173–92. 10.1089/ars.2019.7769 31691574

[26] Moro C Klimcakova E Lolmède K Berlan M Lafontan M Stich V. Atrial natriuretic peptide inhibits the production of adipokines and cytokines linked to inflammation and insulin resistance in human subcutaneous adipose tissue. Diabetologia. (2007) 50:1038–47. 10.1007/s00125-007-0614-3 17318625

[27] Grun LK Teixeira NDR Jr Mengden LV de Bastiani MA Parisi MM Bortolin R. TRF1 as a major contributor for telomeres' shortening in the context of obesity. Free Radic Biol Med. (2018) 129:286–95. 10.1016/j.freeradbiomed.2018.09.039 30268887

[28] Popkin BM Hawkes C. Sweetening of the global diet, particularly beverages: patterns, trends, and policy responses. Lancet Diabetes Endocrinol. (2016) 4:174–86. 10.1016/S2213-8587(15)00419-2 26654575 PMC4733620

[29] Ryerson AB Eheman CR Altekruse SF Ward JW Jemal A Sherman RL. Annual report to the nation on the status of cancer, 1975–2012, featuring the increasing incidence of liver cancer. Cancer. (2016) 122:1312–37. 10.1002/cncr.29936 26959385 PMC4840031

[30] Francois-Vaughan H Adebayo AO Brilliant KE Parry NMA Gruppuso PA Sanders JA. Persistent effect of mTOR inhibition on preneoplastic foci progression and gene expression in a rat model of hepatocellular carcinoma. Carcinogenesis. (2016) 37:408–19. 10.1093/carcin/bgw016 26905589 PMC5006212

[31] Liu ZJ Zhu CF. Causal relationship between insulin resistance and sarcopenia. Diabetol Metab Syndr. (2023) 15:46. 10.1186/s13098-023-01022-z 36918975 PMC10015682

[32] Chan DC Watts GF Gan S Wong AT Ooi EM Barrett PH. Nonalcoholic fatty liver disease as the transducer of hepatic oversecretion of very-low-density lipoprotein-apolipoprotein B-100 in obesity. Arterioscler Thromb Vasc Biol. (2010) 30:1043–50. 10.1161/ATVBAHA.109.202275 20150556

[33] Rossi AP Fantin F Zamboni GA Mazzali G Zoico E Bambace C. Effect of moderate weight loss on hepatic, pancreatic and visceral lipids in obese subjects. Nutr Diabetes. (2012) 2:e32. 10.1038/nutd.2012.5 23449531 PMC3341708

[34] Global Global burden of 288 causes of death and life expectancy decomposition in 204 countries and territories and 811 subnational locations 1990–2021: 1990–2021: a systematic analysis for the Global Burden of Disease Study 2021. Lancet. (2024) 403:2100–32. 10.1016/S0140-6736(24)00367-2 38582094 PMC11126520

[35] Chimed T Sandagdorj T Znaor A Laversanne M Tseveen B Genden P. Cancer incidence and cancer control in Mongolia: results from the national cancer registry 2008–12. Int J Cancer. (2017) 140:302–9. 10.1002/ijc.30463 27716912

[36] Wang Z Liang Z Dong X Gao L Zhou S Yin H. Health policy competencies in regional organizations: a retrospective analysis for 76 regional organizations from 1945 to 2015. Global Health. (2024) 20:17. 10.1186/s12992-024-01023-1 38409001 PMC10895825

[37] Mills A. Health care systems in low- and middle-income countries. N Engl J Med. (2014) 370:552–7. 10.1056/NEJMra1110897 24499213

[38] Hennekens CH Sherling DH Caceres A Benson K Rubenstein A Ferris AH. Navigating the global pandemic in pediatric overweight and obesity: emerging challenges and proposed solutions. Matern Child Health J. (2024) 28:2001–5. 10.1007/s10995-024-04001-6 39316253

[39] Perng W Cantoral A Soria-Contreras DC Betanzos-Robledo L Kordas K Liu Y. Exposure to obesogenic endocrine disrupting chemicals and obesity among youth of latino or hispanic origin in the United States and Latin America: a lifecourse perspective. Obes Rev. (2021) 22:e13245. 10.1111/obr.13245 33951277 PMC8217151

[40] Bundschuh J Schneider J Alam MA Niazi NK Herath I Parvez F. Seven potential sources of arsenic pollution in Latin America and their environmental and health impacts. Sci Total Environ. (2021) 780:146274. 10.1016/j.scitotenv.2021.146274 34030289

[41] Kruk ME Gage AD Arsenault C Jordan K Leslie HH Roder-DeWan S. High-quality health systems in the sustainable development goals era: time for a revolution. Lancet Glob Health. (2018) 6:e1196–252. 10.1016/S2214-109X(18)30386-3 30196093 PMC7734391

[42] Kocarnik JM Compton K Dean FE Fu W Gaw BL Harvey JD. Cancer incidence, mortality, years of life lost, years lived with disability, and disability-adjusted life years for 29 cancer groups from 2010 to 2019: a systematic analysis for the global burden of disease study 2019. JAMA Oncol. (2022) 8:420–44. 10.1001/jamaoncol.2021.6987 34967848 PMC8719276

[43] Kim D Manikat R Cholankeril G Ahmed A. Trends in mortality of liver cancer before and during the COVID-19 pandemic, 2017–2021. Liver Int. (2023) 43:1865–70. 10.1111/liv.15668 37387517

[44] Muñoz-Martínez S Sapena V Forner A Nault JC Sapisochin G Rimassa L. Assessing the impact of COVID-19 on liver cancer management (CERO-19). JHEP Rep. (2021) 3:100260. 10.1016/j.jhepr.2021.100260 33644725 PMC7901294

[45] Vilar-Gomez E Calzadilla-Bertot L Wai-Sun Wong V Castellanos M Aller-de la Fuente R Metwally M. Fibrosis severity as a determinant of cause-specific mortality in patients with advanced nonalcoholic fatty liver disease: a multi-national cohort study. Gastroenterology. (2018) 155:443–57.e417. 10.1053/j.gastro.2018.04.034 29733831

[46] Degenhardt L Charlson F Stanaway J Larney S Alexander LT Hickman M. Estimating the burden of disease attributable to injecting drug use as a risk factor for HIV, hepatitis C, and hepatitis B: findings from the global burden of disease study 2013. Lancet Infect Dis. (2016) 16:1385–98. 10.1016/S1473-3099(16)30325-5 27665254

[47] Nelson PK Mathers BM Cowie B Hagan H Des Jarlais D Horyniak D. Global epidemiology of hepatitis B and hepatitis C in people who inject drugs: results of systematic reviews. Lancet. (2011) 378:571–83. 10.1016/S0140-6736(11)61097-0 21802134 PMC3285467

[48] Kulhánová I Znaor A Shield KD Arnold M Vignat J Charafeddine M. Proportion of cancers attributable to major lifestyle and environmental risk factors in the Eastern Mediterranean region. Int J Cancer. (2020) 146:646–56. 10.1002/ijc.32284 30882889

[49] Poirier AE Ruan Y Volesky KD King WD O'Sullivan DE Gogna P. The current and future burden of cancer attributable to modifiable risk factors in Canada: summary of results. Prev Med. (2019) 122:140–7. 10.1016/j.ypmed.2019.04.007 31078167

[50] Whiteman DC Webb PM Green AC Neale RE Fritschi L Bain CJ. Cancers in Australia in 2010 attributable to modifiable factors: summary and conclusions. Aust N Z J Public Health. (2015) 39:477–84. 10.1111/1753-6405.12471 26437735 PMC4606779

[51] Chen Y Yi Q Mao Y. Cluster of liver cancer and immigration: a geographic analysis of incidence data for Ontario 1998–2002. Int J Health Geogr. (2008) 7:28. 10.1186/1476-072X-7-28 18518988 PMC2426679

[52] Malagón T Morais S Tope P El-Zein M Franco EL. Site-specific cancer incidence by race and immigration status in Canada 2006–2015: a population-based data linkage study. Cancer Epidemiol Biomarkers Prev. (2023) 32:906–18. 10.1158/1055-9965.EPI-22-1191 36788437

[53] Baumeister SE Schlesinger S Aleksandrova K Jochem C Jenab M Gunter MJ. Association between physical activity and risk of hepatobiliary cancers: a multinational cohort study. J Hepatol. (2019) 70:885–92. 10.1016/j.jhep.2018.12.014 30582978

[54] Thomas S Durrheim D Islam F Higgins H Cashman P. Improved childhood immunization coverage using the world health organization's tailoring immunization programmes guide (TIP) in a regional centre in Australia. Vaccine. (2022) 40:18–20. 10.1016/j.vaccine.2021.11.067 34863617

[55] Trevisani F De Notariis S Rapaccini G Farinati F Benvegnù L Zoli M. Semiannual and annual surveillance of cirrhotic patients for hepatocellular carcinoma: effects on cancer stage and patient survival (Italian experience). Am J Gastroenterol. (2002) 97:734–44. 10.1111/j.1572-0241.2002.05557.x 11922571

[56] Papanicolas I Woskie LR Jha AK. Health care spending in the United States and other high-income countries. JAMA. (2018) 319:1024–39. 10.1001/jama.2018.1150 29536101

[57] Bitton A Ratcliffe HL Veillard JH Kress DH Barkley S Kimball M. Primary health care as a foundation for strengthening health systems in low- and middle-income countries. J Gen Intern Med. (2017) 32:566–71. 10.1007/s11606-016-3898-5 27943038 PMC5400754

[58] Ferlay J Soerjomataram I Dikshit R Eser S Mathers C Rebelo M. Cancer incidence and mortality worldwide: sources, methods and major patterns in GLOBOCAN 2012. Int J Cancer. (2015) 136:E359–386. 10.1002/ijc.29210 25220842

[59] Bhaskaran K Douglas I Forbes H dos-Santos-Silva I Leon DA Smeeth L. Body-mass index and risk of 22 specific cancers: a population-based cohort study of 5·24 million UK adults. Lancet. (2014) 384:755–65. 10.1016/S0140-6736(14)60892-8 25129328 PMC4151483

[60] Nataraj K Schonfeld M Rodriguez A Sharma M Weinman S Tikhanovich I. Androgen effects on alcohol-induced liver fibrosis are controlled by a notch-dependent epigenetic switch. Cell Mol Gastroenterol Hepatol. (2025) 19:101414. 10.1016/j.jcmgh.2024.101414 39349250 PMC11609386

[61] Buendía-González FO Legorreta-Herrera M. The similarities and differences between the effects of testosterone and DHEA on the innate and adaptive immune response. Biomolecules. (2022) 12:1768. 10.3390/biom12121768 36551196 PMC9775255

[62] Bui TT Park E Kang HY Oh JK. Combined effects of smoking and alcohol consumption on the risk of liver cancer according to metabolic syndrome: a nested case-control study in South Korea. Int J Cancer. (2024) 155:654–65. 10.1002/ijc.34935 38533737

[63] Prieto J. Inflammation, HCC and sex: IL-6 in the centre of the triangle. J Hepatol. (2008) 48:380–1. 10.1016/j.jhep.2007.11.007 18093689

[64] Tuo JY Li HL Wang J Fang J Tan YT Xiang YB. Menstrual factors, reproductive history and liver cancer risk: findings from a prospective cohort study in chinese women. Cancer Epidemiol Biomarkers Prev. (2022) 31:2046–53. 10.1158/1055-9965.EPI-22-0439 35984984 PMC9633397

[65] del Río-Navarro BE Velázquez-Monroy O Sánchez-Castillo CP Lara-Esqueda A Berber A Fanghänel G. The high prevalence of overweight and obesity in Mexican children. Obes Res. (2004) 12:215–23. 10.1038/oby.2004.28 14981213

[66] Varanasi LC Subasinghe A Jayasinghe YL Callegari ET Garland SM Gorelik A. Polycystic ovarian syndrome: prevalence and impact on the wellbeing of Australian women aged 16–29 years. Aust N Z J Obstet Gynaecol. (2018) 58:222–33. 10.1111/ajo.12730 29052216

[67] Peliganga LB Horta MAP Lewis-Ximenez LL. Enduring challenges despite progress in preventing mother-to-child transmission of hepatitis B virus in angola. Pathogens. (2022) 11:225. 10.3390/pathogens11020225 35215168 PMC8874832

[68] Nyati LH Pettifor JM Norris SA. The prevalence of malnutrition and growth percentiles for urban South African children. BMC Public Health. (2019) 19:492. 10.1186/s12889-019-6794-1 31046727 PMC6498578

[69] Bullock A Sheff K. Incidence trends of type 1 and type 2 diabetes among youths, 2002–2012. N Engl J Med. (2017) 377:301. 10.1056/NEJMc1706291 28727405

[70] Estes C Anstee QM Arias-Loste MT Bantel H Bellentani S Caballeria J. Modeling NAFLD disease burden in China, France, Germany, Italy, Japan, Spain, United Kingdom, and United States for the period 2016–2030. J Hepatol. (2018) 69:896–904. 10.1016/j.jhep.2018.05.036 29886156

[71] Asrani SK Devarbhavi H Eaton J Kamath PS. Burden of liver diseases in the world. J Hepatol. (2019) 70:151–71. 10.1016/j.jhep.2018.09.014 30266282

[72] Hsu YC Huang DQ Nguyen MH. Global burden of hepatitis B virus: current status, missed opportunities and a call for action. Nat Rev Gastroenterol Hepatol. (2023) 20:524–37. 10.1038/s41575-023-00760-9 37024566

[73] Global prevalence cascade cascade of care and and prophylaxis coverage of hepatitis B in 2022: a modelling study. Lancet Gastroenterol Hepatol. (2023) 8:879–907. 10.1016/S2468-1253(23)00197-8 37517414

[74] McNaughton AL Lourenço J Hattingh L Adland E Daniels S Van Zyl A. HBV vaccination and PMTCT as elimination tools in the presence of HIV: insights from a clinical cohort and dynamic model. BMC Med. (2019) 17:43. 10.1186/s12916-019-1269-x 30786896 PMC6383254

[75] Kee KM Lu SN. Hospital- and community-based screenings for hepatocellular carcinoma in Taiwan. Oncology. (2011) 81:36–40. 10.1159/000333257 22212934

[76] Huang K Lin S. Nationwide vaccination: a success story in Taiwan. Vaccine. (2000) 18:S35–38. 10.1016/S0264-410X(99)00460-0 10683542

[77] Bailey H Turkova A Thorne C. Syphilis, hepatitis C and HIV in Eastern Europe. Curr Opin Infect Dis. (2017) 30:93–100. 10.1097/QCO.0000000000000326 27755143

[78] Jaquet A Muula G Ekouevi DK Wandeler G. Elimination of viral hepatitis in low and middle-income countries: epidemiological research gaps. Curr Epidemiol Rep. (2021) 8:89–96. 10.1007/s40471-021-00273-6 34532216 PMC8443244

