# Supplementary material for: Global, regional, and national burden of liver cancer in adolescents and young adults from 1990 to 2021: an analysis of the global burden of disease study 2021 and forecast to 2040
Source: Front Public Health. 2025 Mar 10;13:1547106. doi: 10.3389/fpubh.2025.1547106 (PMC11931027; doi:10.3389/fpubh.2025.1547106)
Supplement: Supplementary file 1 [file Table_1.docx]

**Table S1.** **Incidence of liver cancer among AYA between 1990 and 2021 at national level.**

| Nations | Number of cases, 1990 | Age-standardized rate per 100,000 population, 1990 | Number of cases, 2021 | Age-standardized rate per 100,000 population, 2021 | Estimated annual percentage change, 1990–2021 |
| --- | --- | --- | --- | --- | --- |
| Afghanistan | 21.24(14.88-30.27) | 0.93 (1.53 - 0.53) | 73.83(51.67-107.61) | 0.75 (1.20 - 0.43) | -1.37(-1.67--1.07) |
| Albania | 12.65(9.33-16.47) | 0.94 (1.31 - 0.65) | 5.46(3.98-7.57) | 0.57 (0.88 - 0.36) | -2.12(-2.39--1.84) |
| Algeria | 14.52(10.82-19.39) | 0.17 (0.26 - 0.11) | 37.47(25.76-53.69) | 0.20 (0.31 - 0.12) | 0.27(0.15-0.39) |
| American Samoa | 0.10(0.07-0.15) | 0.57 (0.93 - 0.34) | 0.18(0.12-0.26) | 1.10 (1.72 - 0.66) | 2.65(2.17-3.12) |
| Andorra | 0.22(0.14-0.32) | 0.81 (1.36 - 0.45) | 0.25(0.15-0.38) | 0.85 (1.43 - 0.44) | 0.06(0.00-0.12) |
| Angola | 47.55(6.84-151.86) | 1.39 (4.67 - 0.19) | 90.88(21.23-267.82) | 0.87 (2.56 - 0.19) | -1.62(-1.70--1.53) |
| Antigua and Barbuda | 0.06(0.06-0.07) | 0.26 (0.31 - 0.23) | 0.06(0.05-0.06) | 0.15 (0.18 - 0.13) | -1.39(-1.73--1.05) |
| Argentina | 5.55(4.60-6.77) | 0.05 (0.06 - 0.04) | 18.22(15.65-21.00) | 0.10 (0.13 - 0.08) | 2.94(2.77-3.10) |
| Armenia | 6.98(5.83-8.57) | 0.49 (0.60 - 0.40) | 4.67(3.82-5.59) | 0.38 (0.45 - 0.31) | -0.95(-1.35--0.54) |
| Australia | 14.87(13.20-16.73) | 0.21 (0.26 - 0.17) | 47.96(40.76-56.67) | 0.50 (0.64 - 0.38) | 2.82(2.68-2.95) |
| Austria | 6.93(6.11-7.88) | 0.23 (0.29 - 0.18) | 11.17(9.31-13.06) | 0.36 (0.46 - 0.27) | 1.02(0.84-1.21) |
| Azerbaijan | 17.31(9.80-27.70) | 0.60 (0.98 - 0.32) | 25.50(14.04-43.83) | 0.55 (0.96 - 0.29) | -1.26(-1.56--0.97) |
| Bahamas | 0.43(0.38-0.49) | 0.40 (0.47 - 0.34) | 0.62(0.49-0.78) | 0.40 (0.51 - 0.31) | -0.58(-0.89--0.27) |
| Bahrain | 0.84(0.64-1.19) | 0.31 (0.49 - 0.20) | 1.78(1.23-2.48) | 0.23 (0.37 - 0.14) | -1.82(-2.41--1.22) |
| Bangladesh | 89.70(62.88-131.86) | 0.24 (0.42 - 0.14) | 160.66(97.82-271.48) | 0.24 (0.47 - 0.12) | -0.18(-0.32--0.04) |
| Barbados | 0.24(0.21-0.27) | 0.22 (0.25 - 0.19) | 0.21(0.16-0.27) | 0.20 (0.26 - 0.15) | -0.84(-1.10--0.59) |
| Belarus | 8.73(7.28-10.68) | 0.21 (0.28 - 0.15) | 8.16(6.24-10.33) | 0.23 (0.31 - 0.17) | -0.42(-0.92-0.08) |
| Belgium | 6.88(5.98-7.71) | 0.18 (0.22 - 0.14) | 9.53(8.10-11.31) | 0.25 (0.32 - 0.19) | 0.87(0.68-1.05) |
| Belize | 0.10(0.09-0.12) | 0.17 (0.20 - 0.14) | 0.50(0.44-0.57) | 0.29 (0.33 - 0.24) | 0.96(0.58-1.34) |
| Benin | 45.06(22.93-71.46) | 3.04 (5.60 - 1.35) | 95.77(61.30-136.76) | 2.15 (3.63 - 1.15) | -1.59(-1.86--1.32) |
| Bermuda | 0.06(0.06-0.07) | 0.22 (0.25 - 0.19) | 0.02(0.02-0.03) | 0.12 (0.15 - 0.10) | -1.92(-2.43--1.41) |
| Bhutan | 0.85(0.46-1.35) | 0.39 (0.73 - 0.19) | 1.43(0.83-2.31) | 0.41 (0.73 - 0.21) | -0.22(-0.39--0.05) |
| Bolivia (Plurinational State of) | 7.81(5.21-10.98) | 0.35 (0.55 - 0.21) | 12.77(8.12-20.41) | 0.27 (0.46 - 0.15) | -1.33(-1.61--1.05) |
| Bosnia and Herzegovina | 9.82(8.22-11.70) | 0.51 (0.65 - 0.38) | 2.70(1.89-3.53) | 0.24 (0.34 - 0.16) | -3.16(-3.71--2.61) |
| Botswana | 3.73(1.56-7.90) | 0.88 (1.99 - 0.33) | 11.15(4.68-26.49) | 1.00 (2.52 - 0.37) | -0.83(-1.34--0.31) |
| Brazil | 108.89(104.44-113.82) | 0.19 (0.20 - 0.18) | 143.71(136.08-150.59) | 0.16 (0.17 - 0.15) | -0.06(-0.30-0.18) |
| Brunei Darussalam | 1.16(0.83-1.65) | 0.96 (1.50 - 0.59) | 1.51(1.10-2.08) | 0.66 (1.02 - 0.42) | -1.61(-1.77--1.44) |
| Bulgaria | 18.97(14.48-23.71) | 0.60 (0.76 - 0.45) | 5.52(4.02-7.67) | 0.25 (0.34 - 0.18) | -2.58(-2.95--2.22) |
| Burkina Faso | 100.60(42.07-227.95) | 3.78 (9.08 - 1.45) | 230.79(99.45-448.24) | 3.09 (6.26 - 1.23) | -0.71(-0.92--0.50) |
| Burundi | 11.43(6.97-21.12) | 0.61 (1.20 - 0.30) | 17.45(10.46-28.40) | 0.36 (0.67 - 0.18) | -2.05(-2.18--1.92) |
| Cabo Verde | 2.83(1.98-3.98) | 2.73 (4.57 - 1.56) | 5.68(3.93-8.08) | 2.19 (3.79 - 1.22) | -0.77(-1.11--0.43) |
| Cambodia | 33.83(18.12-60.38) | 1.03 (2.03 - 0.49) | 51.66(24.55-102.29) | 0.71 (1.50 - 0.31) | -1.53(-1.63--1.42) |
| Cameroon | 96.52(66.42-146.62) | 3.01 (5.09 - 1.74) | 255.17(141.83-447.98) | 2.25 (4.43 - 1.10) | -1.28(-1.43--1.12) |
| Canada | 26.23(22.96-29.74) | 0.22 (0.27 - 0.18) | 56.70(47.26-68.30) | 0.44 (0.57 - 0.34) | 2.59(2.38-2.79) |
| Central African Republic | 11.12(4.08-23.60) | 1.26 (2.93 - 0.44) | 14.80(5.70-32.88) | 0.79 (1.82 - 0.28) | -2.13(-2.37--1.88) |
| Chad | 43.24(16.96-94.89) | 2.41 (5.44 - 0.84) | 106.48(56.88-201.07) | 2.10 (4.16 - 0.98) | -0.67(-0.94--0.40) |
| Chile | 4.04(3.21-5.03) | 0.08 (0.10 - 0.06) | 9.60(8.13-11.59) | 0.13 (0.17 - 0.10) | 2.27(1.93-2.61) |
| China | 12175.15(10111.36-14566.97) | 2.41 (2.93 - 1.98) | 11859.96(9320.63-15237.84) | 2.10 (2.72 - 1.62) | -1.13(-1.49--0.76) |
| Colombia | 33.69(30.54-37.06) | 0.26 (0.31 - 0.21) | 35.86(30.09-43.31) | 0.18 (0.23 - 0.14) | -1.33(-1.83--0.83) |
| Comoros | 0.96(0.43-1.47) | 0.66 (1.15 - 0.29) | 1.75(1.19-2.64) | 0.60 (0.99 - 0.33) | -1.05(-1.65--0.45) |
| Congo | 10.98(4.65-21.87) | 1.43 (3.13 - 0.54) | 17.26(7.24-36.22) | 0.83 (1.88 - 0.30) | -2.36(-2.60--2.13) |
| Cook Islands | 0.09(0.06-0.13) | 1.28 (2.05 - 0.77) | 0.08(0.05-0.12) | 1.42 (2.34 - 0.80) | 0.70(0.55-0.86) |
| Costa Rica | 4.69(4.10-5.30) | 0.40 (0.49 - 0.32) | 8.01(6.62-9.73) | 0.40 (0.52 - 0.31) | -0.37(-0.78-0.04) |
| Croatia | 5.01(3.84-6.33) | 0.26 (0.33 - 0.19) | 1.81(1.33-2.39) | 0.13 (0.17 - 0.09) | -2.55(-3.10--2.00) |
| Cuba | 9.73(8.30-11.56) | 0.22 (0.29 - 0.17) | 4.67(3.66-5.74) | 0.12 (0.16 - 0.09) | -2.53(-2.99--2.06) |
| Cyprus | 0.53(0.38-0.78) | 0.17 (0.29 - 0.10) | 0.97(0.68-1.35) | 0.16 (0.25 - 0.10) | -0.99(-1.38--0.60) |
| Czechia | 13.42(11.56-15.69) | 0.33 (0.41 - 0.27) | 3.72(2.83-4.72) | 0.11 (0.15 - 0.08) | -3.87(-4.21--3.54) |
| C么te d'Ivoire | 34.61(20.53-54.39) | 0.85 (1.55 - 0.42) | 54.37(31.61-83.45) | 0.52 (0.90 - 0.26) | -2.02(-2.22--1.82) |
| Democratic People's Republic of Korea | 169.93(81.37-304.66) | 2.23 (4.40 - 0.97) | 168.70(88.68-309.76) | 1.55 (3.01 - 0.76) | -1.61(-1.77--1.45) |
| Democratic Republic of the Congo | 58.22(28.54-125.01) | 0.47 (1.04 - 0.21) | 111.12(51.63-254.82) | 0.35 (0.87 - 0.15) | -1.15(-1.32--0.98) |
| Denmark | 3.12(2.68-3.59) | 0.16 (0.20 - 0.12) | 2.03(1.69-2.45) | 0.11 (0.14 - 0.08) | -1.67(-2.07--1.27) |
| Djibouti | 0.64(0.34-1.15) | 0.45 (0.83 - 0.22) | 2.59(1.36-4.49) | 0.48 (0.87 - 0.23) | 0.14(-0.13-0.42) |
| Dominica | 0.05(0.03-0.08) | 0.19 (0.37 - 0.10) | 0.06(0.04-0.09) | 0.24 (0.40 - 0.13) | -0.01(-0.47-0.46) |
| Dominican Republic | 4.72(3.41-6.35) | 0.18 (0.27 - 0.11) | 11.29(7.75-15.63) | 0.25 (0.39 - 0.15) | 1.08(0.89-1.28) |
| Ecuador | 21.93(19.19-24.87) | 0.59 (0.74 - 0.46) | 22.59(17.40-28.58) | 0.31 (0.42 - 0.22) | -2.53(-3.37--1.67) |
| Egypt | 191.03(135.82-276.69) | 0.98 (1.56 - 0.60) | 405.35(306.37-531.40) | 0.98 (1.38 - 0.66) | 0.25(0.13-0.38) |
| El Salvador | 2.91(2.51-3.42) | 0.16 (0.21 - 0.12) | 3.84(3.02-4.85) | 0.16 (0.21 - 0.11) | 0.40(0.04-0.77) |
| Equatorial Guinea | 0.54(0.26-1.18) | 0.41 (0.94 - 0.18) | 3.67(1.74-6.67) | 0.61 (1.22 - 0.26) | 1.28(0.97-1.60) |
| Eritrea | 5.03(3.01-8.40) | 0.46 (0.87 - 0.25) | 11.66(6.63-21.11) | 0.45 (0.88 - 0.22) | -0.81(-1.07--0.55) |
| Estonia | 2.02(1.68-2.40) | 0.33 (0.43 - 0.25) | 1.02(0.85-1.21) | 0.23 (0.30 - 0.17) | -2.16(-2.45--1.87) |
| Eswatini | 3.19(1.22-6.23) | 1.34 (2.80 - 0.46) | 19.28(5.71-49.08) | 4.14 (11.79 - 1.12) | 3.83(2.28-5.41) |
| Ethiopia | 70.87(45.89-105.55) | 0.44 (0.66 - 0.27) | 114.97(76.99-171.25) | 0.28 (0.44 - 0.19) | -1.97(-2.19--1.75) |
| Fiji | 1.71(1.16-2.50) | 0.58 (0.95 - 0.33) | 2.30(1.46-3.43) | 0.63 (1.06 - 0.35) | 0.64(0.27-1.01) |
| Finland | 6.95(5.79-8.28) | 0.34 (0.43 - 0.27) | 5.96(4.78-7.36) | 0.33 (0.43 - 0.25) | 0.02(-0.19-0.23) |
| France | 69.61(62.25-77.04) | 0.30 (0.36 - 0.25) | 87.21(74.82-105.08) | 0.41 (0.54 - 0.31) | 1.39(1.14-1.65) |
| Gabon | 3.76(1.50-8.66) | 1.13 (2.79 - 0.40) | 6.83(3.56-12.09) | 1.01 (2.00 - 0.44) | -0.73(-0.93--0.54) |
| Gambia | 13.25(8.75-19.05) | 4.25 (6.99 - 2.44) | 45.39(27.70-73.82) | 5.31 (9.95 - 2.72) | 0.15(-0.09-0.39) |
| Georgia | 9.26(7.78-10.85) | 0.43 (0.51 - 0.36) | 2.89(2.46-3.36) | 0.22 (0.26 - 0.19) | -2.51(-2.98--2.04) |
| Germany | 49.71(42.99-57.58) | 0.16 (0.20 - 0.13) | 79.64(67.94-93.52) | 0.28 (0.35 - 0.22) | 1.74(1.48-2.01) |
| Ghana | 148.41(88.00-237.26) | 2.92 (5.19 - 1.53) | 263.90(163.74-431.85) | 1.96 (3.59 - 1.01) | -1.65(-1.98--1.31) |
| Greece | 10.43(9.59-11.34) | 0.27 (0.31 - 0.24) | 15.37(13.62-17.39) | 0.49 (0.56 - 0.42) | -1.14(-1.62--0.66) |
| Greenland | 0.17(0.12-0.23) | 0.62 (0.95 - 0.37) | 0.09(0.05-0.13) | 0.39 (0.63 - 0.22) | 1.49(1.30-1.68) |
| Grenada | 0.08(0.06-0.10) | 0.26 (0.36 - 0.19) | 0.12(0.10-0.15) | 0.31 (0.41 - 0.23) | -0.98(-1.33--0.63) |
| Guam | 0.23(0.19-0.29) | 0.38 (0.52 - 0.28) | 0.61(0.47-0.77) | 1.12 (1.54 - 0.77) | 0.07(-0.37-0.51) |
| Guatemala | 16.86(15.79-17.88) | 0.66 (0.72 - 0.59) | 30.23(26.30-34.47) | 0.49 (0.57 - 0.41) | 4.10(3.90-4.31) |
| Guinea | 68.09(48.39-94.69) | 3.66 (5.69 - 2.17) | 138.03(87.42-206.63) | 3.08 (5.28 - 1.69) | -1.58(-2.33--0.82) |
| Guinea-Bissau | 17.34(7.11-27.79) | 5.53 (10.10 - 2.06) | 27.62(16.29-41.61) | 3.68 (6.53 - 1.87) | -0.40(-0.59--0.20) |
| Guyana | 0.65(0.54-0.80) | 0.22 (0.27 - 0.18) | 0.83(0.62-1.06) | 0.29 (0.38 - 0.21) | -1.66(-1.79--1.53) |
| Haiti | 3.79(2.18-6.69) | 0.17 (0.32 - 0.09) | 6.89(3.33-13.32) | 0.13 (0.26 - 0.06) | 0.24(-0.37-0.86) |
| Honduras | 2.85(2.05-4.00) | 0.19 (0.29 - 0.11) | 5.95(3.34-9.56) | 0.15 (0.27 - 0.08) | -0.92(-1.07--0.77) |
| Hungary | 12.72(10.04-16.27) | 0.31 (0.42 - 0.23) | 3.10(2.35-4.00) | 0.10 (0.14 - 0.07) | -1.34(-1.70--0.97) |
| Iceland | 0.18(0.15-0.20) | 0.17 (0.21 - 0.13) | 0.32(0.26-0.40) | 0.25 (0.33 - 0.19) | -4.29(-4.99--3.58) |
| India | 747.77(662.53-879.28) | 0.24 (0.29 - 0.20) | 1823.98(1541.19-2212.29) | 0.31 (0.38 - 0.25) | 1.04(0.79-1.28) |
| Indonesia | 313.09(203.10-459.21) | 0.45 (0.68 - 0.29) | 543.86(346.32-861.01) | 0.46 (0.74 - 0.29) | 0.61(0.37-0.85) |
| Iran (Islamic Republic of) | 45.21(38.39-55.60) | 0.24 (0.30 - 0.19) | 125.99(109.71-143.28) | 0.31 (0.36 - 0.26) | 0.11(0.03-0.19) |
| Iraq | 23.92(17.18-33.27) | 0.40 (0.62 - 0.24) | 56.99(39.28-87.35) | 0.35 (0.57 - 0.22) | 0.45(-0.19-1.09) |
| Ireland | 1.58(1.39-1.79) | 0.12 (0.15 - 0.10) | 3.01(2.49-3.58) | 0.18 (0.22 - 0.13) | -0.14(-0.34-0.06) |
| Israel | 1.84(1.59-2.12) | 0.10 (0.12 - 0.08) | 4.78(4.03-5.64) | 0.14 (0.19 - 0.11) | 0.83(0.37-1.30) |
| Italy | 74.28(69.01-80.35) | 0.35 (0.38 - 0.32) | 53.92(47.89-60.45) | 0.31 (0.36 - 0.27) | 0.94(0.32-1.55) |
| Jamaica | 0.60(0.49-0.76) | 0.07 (0.09 - 0.05) | 1.61(1.15-2.23) | 0.14 (0.19 - 0.09) | -0.88(-1.18--0.57) |
| Japan | 289.59(276.22-304.56) | 0.61 (0.64 - 0.58) | 119.66(107.55-132.92) | 0.32 (0.36 - 0.29) | 1.28(0.75-1.81) |
| Jordan | 1.81(1.20-2.83) | 0.15 (0.26 - 0.09) | 5.18(3.60-7.37) | 0.10 (0.16 - 0.06) | -2.75(-3.32--2.18) |
| Kazakhstan | 65.49(58.56-73.33) | 0.97 (1.09 - 0.87) | 30.43(25.21-37.26) | 0.40 (0.48 - 0.32) | -1.80(-2.06--1.55) |
| Kenya | 21.74(15.66-32.17) | 0.30 (0.47 - 0.21) | 87.86(62.83-127.50) | 0.46 (0.67 - 0.32) | -3.79(-4.34--3.24) |
| Kiribati | 0.35(0.25-0.49) | 1.28 (2.00 - 0.79) | 0.60(0.38-0.89) | 1.25 (2.06 - 0.71) | 1.39(1.09-1.68) |
| Kuwait | 2.80(2.38-3.33) | 0.31 (0.41 - 0.24) | 1.68(1.36-2.15) | 0.06 (0.09 - 0.05) | -0.43(-0.72--0.14) |
| Kyrgyzstan | 12.28(9.18-16.24) | 0.75 (1.02 - 0.55) | 4.91(3.31-6.75) | 0.18 (0.25 - 0.12) | -4.55(-4.97--4.13) |
| Lao People's Democratic Republic | 17.39(11.10-24.83) | 1.32 (2.20 - 0.71) | 26.57(17.04-40.65) | 0.85 (1.49 - 0.46) | -3.62(-4.14--3.10) |
| Latvia | 2.83(2.34-3.39) | 0.28 (0.37 - 0.21) | 1.54(1.20-1.93) | 0.25 (0.34 - 0.18) | -1.78(-1.92--1.63) |
| Lebanon | 2.93(2.06-4.07) | 0.28 (0.43 - 0.17) | 4.57(3.45-6.09) | 0.18 (0.28 - 0.11) | -1.67(-2.05--1.29) |
| Lesotho | 3.83(1.23-9.94) | 0.81 (2.16 - 0.22) | 22.57(7.42-65.49) | 3.07 (9.87 - 0.87) | -1.54(-1.77--1.32) |
| Liberia | 23.95(11.53-39.22) | 2.91 (5.36 - 1.27) | 59.57(33.57-91.58) | 2.94 (5.29 - 1.48) | 4.50(3.42-5.59) |
| Libya | 8.25(5.65-11.39) | 0.59 (0.97 - 0.35) | 25.36(17.17-36.47) | 0.78 (1.26 - 0.45) | 0.10(-0.12-0.33) |
| Lithuania | 3.53(3.04-4.09) | 0.25 (0.31 - 0.20) | 2.42(1.92-3.14) | 0.28 (0.37 - 0.21) | 1.14(0.96-1.32) |
| Luxembourg | 0.35(0.32-0.37) | 0.22 (0.24 - 0.19) | 0.53(0.46-0.60) | 0.21 (0.24 - 0.18) | -0.03(-0.27-0.20) |
| Madagascar | 21.95(15.13-30.93) | 0.56 (0.88 - 0.32) | 41.04(27.20-63.92) | 0.40 (0.69 - 0.22) | -0.67(-0.96--0.37) |
| Malawi | 14.59(9.51-21.78) | 0.46 (0.79 - 0.25) | 42.49(28.04-63.59) | 0.62 (1.08 - 0.33) | -0.97(-1.10--0.83) |
| Malaysia | 28.52(21.64-38.66) | 0.41 (0.66 - 0.25) | 76.60(56.26-108.22) | 0.53 (0.89 - 0.32) | 0.35(-0.25-0.95) |
| Maldives | 0.34(0.18-0.54) | 0.56 (1.03 - 0.24) | 1.10(0.66-1.69) | 0.32 (0.57 - 0.17) | 0.55(0.33-0.77) |
| Mali | 84.68(64.68-109.01) | 3.26 (4.82 - 2.13) | 249.99(174.38-341.70) | 3.48 (5.75 - 2.00) | -2.75(-3.44--2.07) |
| Malta | 0.14(0.12-0.16) | 0.09 (0.12 - 0.07) | 0.29(0.24-0.35) | 0.19 (0.25 - 0.14) | 0.25(0.14-0.37) |
| Marshall Islands | 0.07(0.05-0.11) | 0.48 (0.89 - 0.25) | 0.16(0.09-0.28) | 0.69 (1.27 - 0.36) | 1.67(1.31-2.03) |
| Mauritania | 38.59(7.92-80.77) | 5.79 (13.63 - 1.07) | 41.99(20.44-70.27) | 2.87 (5.56 - 1.22) | 0.80(0.55-1.05) |
| Mauritius | 1.69(1.56-1.84) | 0.35 (0.39 - 0.31) | 0.29(0.26-0.32) | 0.06 (0.07 - 0.05) | -2.58(-2.70--2.46) |
| Mexico | 46.34(45.39-47.30) | 0.15 (0.16 - 0.15) | 98.96(88.83-109.15) | 0.19 (0.21 - 0.17) | -0.76(-2.62-1.13) |
| Micronesia (Federated States of) | 0.29(0.19-0.45) | 0.83 (1.42 - 0.46) | 0.33(0.19-0.57) | 0.85 (1.59 - 0.43) | 1.12(0.96-1.27) |
| Monaco | 0.03(0.02-0.05) | 0.33 (0.52 - 0.20) | 0.08(0.05-0.13) | 0.80 (1.39 - 0.42) | -0.07(-0.31-0.17) |
| Mongolia | 39.25(25.53-58.24) | 5.26 (8.46 - 2.99) | 63.70(43.74-91.92) | 4.62 (7.02 - 2.99) | 2.78(2.14-3.42) |
| Montenegro | 1.27(1.00-1.59) | 0.50 (0.65 - 0.38) | 0.89(0.67-1.18) | 0.40 (0.54 - 0.30) | -0.67(-1.01--0.34) |
| Morocco | 3.91(2.76-5.37) | 0.04 (0.07 - 0.03) | 7.24(4.85-11.68) | 0.05 (0.09 - 0.03) | -0.69(-0.92--0.46) |
| Mozambique | 38.42(21.03-61.28) | 0.93 (1.67 - 0.45) | 135.27(62.50-283.58) | 1.42 (3.31 - 0.56) | 0.30(0.23-0.37) |
| Myanmar | 73.39(30.71-143.90) | 0.48 (1.02 - 0.18) | 87.67(43.43-175.00) | 0.40 (0.87 - 0.18) | 1.48(1.09-1.86) |
| Namibia | 1.11(0.60-2.05) | 0.24 (0.47 - 0.11) | 3.67(2.15-5.95) | 0.38 (0.71 - 0.19) | -0.78(-0.85--0.71) |
| Nauru | 0.05(0.03-0.07) | 1.31 (2.21 - 0.72) | 0.05(0.03-0.09) | 1.22 (2.18 - 0.56) | 0.93(0.09-1.78) |
| Nepal | 13.35(8.92-19.47) | 0.20 (0.35 - 0.11) | 38.90(25.52-55.87) | 0.32 (0.53 - 0.17) | -0.55(-0.69--0.42) |
| Netherlands | 6.03(5.23-7.15) | 0.10 (0.12 - 0.08) | 7.26(5.94-8.72) | 0.13 (0.16 - 0.10) | 1.67(1.47-1.87) |
| New Zealand | 4.66(3.94-5.58) | 0.34 (0.42 - 0.27) | 13.35(10.99-16.06) | 0.71 (0.91 - 0.56) | 0.86(0.70-1.02) |
| Nicaragua | 3.14(2.55-3.92) | 0.25 (0.36 - 0.17) | 6.76(5.04-8.86) | 0.24 (0.37 - 0.16) | 2.09(1.90-2.28) |
| Niger | 62.72(27.54-124.56) | 2.67 (5.98 - 1.03) | 95.46(56.63-170.08) | 1.38 (2.65 - 0.69) | 0.09(-0.25-0.44) |
| Nigeria | 169.55(71.10-330.71) | 0.58 (1.16 - 0.24) | 344.38(192.04-565.28) | 0.46 (0.75 - 0.26) | -2.42(-2.55--2.29) |
| Niue | 0.00(0.00-0.01) | 0.62 (1.09 - 0.33) | 0.00(0.00-0.01) | 0.66 (1.23 - 0.35) | -1.19(-1.39--0.98) |
| North Macedonia | 5.36(4.55-6.48) | 0.67 (0.89 - 0.51) | 3.26(2.47-4.17) | 0.37 (0.52 - 0.26) | -0.06(-0.30-0.18) |
| Northern Mariana Islands | 0.16(0.10-0.24) | 0.65 (1.10 - 0.36) | 0.06(0.05-0.09) | 0.39 (0.63 - 0.23) | -2.26(-2.63--1.89) |
| Norway | 2.86(2.66-3.08) | 0.17 (0.19 - 0.16) | 8.74(7.69-9.89) | 0.46 (0.53 - 0.40) | -1.54(-2.02--1.07) |
| Oman | 1.71(1.02-2.91) | 0.20 (0.39 - 0.10) | 8.12(5.30-11.58) | 0.29 (0.48 - 0.16) | 2.82(2.36-3.27) |
| Pakistan | 134.60(101.08-181.11) | 0.39 (0.56 - 0.26) | 478.11(354.93-643.27) | 0.52 (0.74 - 0.35) | 1.25(1.03-1.47) |
| Palau | 0.07(0.04-0.13) | 1.14 (2.35 - 0.48) | 0.12(0.07-0.18) | 1.75 (3.14 - 0.85) | 0.71(0.40-1.03) |
| Palestine | 2.80(1.91-3.98) | 0.45 (0.72 - 0.27) | 6.51(5.01-8.30) | 0.33 (0.49 - 0.22) | 1.44(1.09-1.80) |
| Panama | 2.03(1.90-2.15) | 0.22 (0.24 - 0.20) | 3.96(3.25-4.76) | 0.24 (0.30 - 0.20) | -1.28(-1.61--0.94) |
| Papua New Guinea | 7.35(2.88-17.95) | 0.51 (1.33 - 0.18) | 14.59(6.86-32.81) | 0.36 (0.88 - 0.15) | 0.66(0.19-1.14) |
| Paraguay | 2.48(1.97-3.07) | 0.17 (0.24 - 0.12) | 7.76(5.32-10.99) | 0.26 (0.40 - 0.15) | -1.33(-1.47--1.19) |
| Peru | 22.73(16.10-32.92) | 0.28 (0.45 - 0.17) | 42.12(27.88-61.31) | 0.28 (0.46 - 0.16) | 1.65(1.45-1.85) |
| Philippines | 367.00(267.92-430.17) | 1.65 (1.99 - 1.19) | 414.03(341.94-495.66) | 0.93 (1.14 - 0.76) | -0.16(-0.34-0.03) |
| Poland | 7.98(7.28-8.95) | 0.05 (0.06 - 0.04) | 16.13(14.55-17.90) | 0.11 (0.12 - 0.10) | -2.05(-2.17--1.93) |
| Portugal | 8.07(6.91-9.37) | 0.22 (0.27 - 0.17) | 6.93(5.77-8.63) | 0.21 (0.28 - 0.16) | 3.38(2.52-4.24) |
| Puerto Rico | 4.50(3.70-5.45) | 0.33 (0.44 - 0.24) | 3.99(3.17-4.93) | 0.37 (0.51 - 0.27) | -0.91(-1.29--0.53) |
| Qatar | 1.21(0.83-1.88) | 0.45 (0.76 - 0.26) | 10.87(7.54-14.97) | 0.55 (0.90 - 0.32) | -0.78(-1.63-0.09) |
| Republic of Korea | 585.32(398.50-828.58) | 2.98 (4.73 - 1.77) | 270.06(197.66-364.80) | 1.42 (2.06 - 0.95) | -0.45(-0.90--0.00) |
| Republic of Moldova | 5.21(4.87-5.61) | 0.28 (0.31 - 0.26) | 4.00(3.48-4.50) | 0.27 (0.31 - 0.23) | -2.88(-3.04--2.71) |
| Romania | 13.79(10.92-17.00) | 0.16 (0.21 - 0.11) | 9.29(7.33-11.75) | 0.16 (0.21 - 0.11) | -0.27(-0.49--0.05) |
| Russian Federation | 115.68(111.87-119.19) | 0.19 (0.19 - 0.18) | 147.64(137.59-157.35) | 0.26 (0.28 - 0.24) | -0.32(-0.59--0.04) |
| Rwanda | 22.33(14.54-32.13) | 0.91 (1.49 - 0.49) | 27.10(16.96-43.22) | 0.52 (0.89 - 0.28) | 1.59(0.96-2.23) |
| Saint Kitts and Nevis | 0.06(0.06-0.07) | 0.41 (0.49 - 0.35) | 0.04(0.03-0.05) | 0.16 (0.22 - 0.11) | -2.91(-3.29--2.53) |
| Saint Lucia | 0.11(0.09-0.13) | 0.23 (0.28 - 0.19) | 0.14(0.11-0.16) | 0.20 (0.24 - 0.16) | -2.82(-3.86--1.77) |
| Saint Vincent and the Grenadines | 0.14(0.12-0.16) | 0.36 (0.43 - 0.31) | 0.13(0.11-0.15) | 0.32 (0.38 - 0.26) | -1.19(-1.53--0.84) |
| Samoa | 0.26(0.16-0.40) | 0.49 (0.85 - 0.26) | 0.36(0.20-0.59) | 0.50 (0.91 - 0.25) | -1.09(-1.35--0.82) |
| San Marino | 0.01(0.01-0.02) | 0.12 (0.19 - 0.07) | 0.02(0.01-0.03) | 0.21 (0.36 - 0.11) | -0.17(-0.30--0.03) |
| Sao Tome and Principe | 0.21(0.13-0.30) | 0.63 (1.10 - 0.33) | 0.40(0.21-0.83) | 0.48 (1.04 - 0.21) | 2.02(1.91-2.13) |
| Saudi Arabia | 26.20(16.35-41.03) | 0.44 (0.76 - 0.24) | 64.07(40.06-92.06) | 0.30 (0.48 - 0.17) | -0.89(-1.30--0.48) |
| Senegal | 51.89(28.85-78.45) | 2.25 (3.89 - 1.16) | 82.35(52.44-122.09) | 1.47 (2.44 - 0.82) | -2.07(-2.46--1.68) |
| Serbia | 14.09(10.15-19.14) | 0.38 (0.57 - 0.24) | 8.21(5.61-12.03) | 0.25 (0.40 - 0.15) | -1.31(-1.43--1.19) |
| Seychelles | 0.25(0.18-0.34) | 0.95 (1.51 - 0.56) | 0.16(0.11-0.23) | 0.37 (0.62 - 0.20) | -1.57(-1.65--1.48) |
| Sierra Leone | 35.61(11.19-68.14) | 2.51 (5.41 - 0.73) | 52.01(32.81-79.16) | 1.61 (2.83 - 0.85) | -2.56(-2.99--2.12) |
| Singapore | 11.01(9.47-12.83) | 0.72 (0.90 - 0.57) | 7.89(6.31-9.90) | 0.31 (0.41 - 0.23) | -1.47(-1.70--1.24) |
| Slovakia | 9.31(6.59-13.03) | 0.42 (0.60 - 0.29) | 4.90(3.08-7.53) | 0.24 (0.38 - 0.15) | -3.62(-4.06--3.19) |
| Slovenia | 3.40(3.00-3.91) | 0.41 (0.51 - 0.33) | 1.13(0.91-1.39) | 0.17 (0.23 - 0.12) | -1.94(-2.26--1.62) |
| Solomon Islands | 0.91(0.27-1.93) | 0.91 (2.08 - 0.25) | 2.07(1.24-3.25) | 0.84 (1.53 - 0.43) | -3.22(-3.54--2.90) |
| Somalia | 23.51(9.96-43.24) | 0.90 (1.83 - 0.36) | 59.71(26.33-120.11) | 0.86 (1.78 - 0.33) | -0.32(-0.61--0.03) |
| South Africa | 199.91(129.26-298.96) | 1.46 (2.16 - 0.92) | 336.27(277.61-404.25) | 1.29 (1.62 - 1.03) | -0.43(-0.66--0.19) |
| South Sudan | 10.97(6.64-17.47) | 0.58 (1.06 - 0.29) | 22.17(13.81-34.48) | 0.72 (1.24 - 0.37) | -1.27(-2.41--0.12) |
| Spain | 50.17(44.76-55.78) | 0.35 (0.43 - 0.28) | 51.70(42.48-63.65) | 0.37 (0.48 - 0.28) | 0.40(0.12-0.68) |
| Sri Lanka | 14.97(11.01-20.12) | 0.21 (0.34 - 0.13) | 12.42(7.70-18.77) | 0.15 (0.26 - 0.08) | -0.16(-0.44-0.11) |
| Sudan | 24.12(13.63-42.69) | 0.37 (0.69 - 0.18) | 59.98(35.81-93.91) | 0.36 (0.62 - 0.18) | -2.36(-2.95--1.77) |
| Suriname | 0.34(0.21-0.48) | 0.25 (0.40 - 0.14) | 0.61(0.40-0.89) | 0.28 (0.48 - 0.16) | 0.09(-0.07-0.25) |
| Sweden | 6.88(6.23-7.56) | 0.23 (0.26 - 0.19) | 8.88(7.64-10.31) | 0.25 (0.31 - 0.20) | 0.31(0.14-0.47) |
| Switzerland | 10.21(8.52-12.26) | 0.37 (0.47 - 0.28) | 5.75(4.69-7.13) | 0.18 (0.24 - 0.14) | 1.29(0.18-2.42) |
| Syrian Arab Republic | 27.67(21.28-36.39) | 0.71 (0.98 - 0.50) | 18.59(12.74-26.48) | 0.40 (0.59 - 0.26) | -2.58(-2.76--2.40) |
| Taiwan (Province of China) | 170.82(153.40-188.26) | 1.81 (2.13 - 1.52) | 120.22(98.53-146.72) | 1.33 (1.71 - 1.01) | -2.20(-2.74--1.66) |
| Tajikistan | 12.84(7.65-20.31) | 0.71 (1.15 - 0.40) | 18.07(11.08-27.75) | 0.44 (0.71 - 0.25) | -0.50(-1.36-0.36) |
| Thailand | 354.53(244.73-509.25) | 1.49 (2.33 - 0.92) | 437.95(288.93-636.58) | 1.85 (2.99 - 1.09) | -2.41(-2.74--2.07) |
| Timor-Leste | 1.06(0.58-1.81) | 0.37 (0.66 - 0.19) | 1.32(0.72-2.54) | 0.29 (0.56 - 0.14) | -0.41(-0.77--0.05) |
| Togo | 16.84(11.51-25.64) | 1.48 (2.51 - 0.88) | 38.54(23.65-66.89) | 1.26 (2.32 - 0.69) | -1.02(-1.50--0.54) |
| Tokelau | 0.00(0.00-0.01) | 0.57 (1.14 - 0.28) | 0.00(0.00-0.01) | 0.72 (1.31 - 0.39) | -0.84(-1.03--0.64) |
| Tonga | 0.70(0.46-1.10) | 2.55 (4.24 - 1.47) | 0.98(0.58-1.65) | 2.86 (5.22 - 1.53) | 0.71(0.51-0.90) |
| Trinidad and Tobago | 1.03(0.93-1.15) | 0.22 (0.25 - 0.19) | 1.51(1.14-1.89) | 0.26 (0.33 - 0.20) | 0.03(-0.36-0.43) |
| Tunisia | 4.54(3.27-6.18) | 0.15 (0.25 - 0.09) | 9.40(6.15-13.66) | 0.19 (0.31 - 0.11) | 0.18(-0.05-0.41) |
| Turkey | 47.47(35.67-62.50) | 0.22 (0.33 - 0.14) | 63.67(47.54-84.61) | 0.19 (0.29 - 0.12) | 0.63(0.56-0.70) |
| Turkmenistan | 7.90(7.05-8.76) | 0.57 (0.64 - 0.50) | 10.82(8.05-14.27) | 0.52 (0.69 - 0.39) | -0.84(-1.37--0.31) |
| Tuvalu | 0.02(0.02-0.04) | 0.67 (1.12 - 0.39) | 0.03(0.02-0.05) | 0.75 (1.30 - 0.41) | -0.14(-0.25--0.03) |
| Uganda | 41.81(27.84-63.79) | 0.81 (1.33 - 0.48) | 143.75(97.12-205.34) | 1.01 (1.67 - 0.59) | 0.28(0.14-0.42) |
| Ukraine | 44.36(36.60-52.99) | 0.22 (0.29 - 0.17) | 30.52(21.32-40.61) | 0.18 (0.25 - 0.12) | -0.00(-0.48-0.48) |
| United Arab Emirates | 4.65(2.90-7.35) | 0.44 (0.85 - 0.24) | 32.52(20.55-47.34) | 0.64 (1.02 - 0.36) | -2.03(-2.82--1.23) |
| United Kingdom | 39.56(38.46-40.72) | 0.19 (0.19 - 0.18) | 161.22(153.73-169.46) | 0.68 (0.72 - 0.64) | 1.38(1.10-1.66) |
| United Republic of Tanzania | 59.82(40.77-82.51) | 0.74 (1.21 - 0.43) | 121.55(78.07-183.71) | 0.60 (1.00 - 0.33) | 5.15(4.86-5.45) |
| United States of America | 299.14(291.18-306.83) | 0.27 (0.28 - 0.26) | 561.26(533.27-588.53) | 0.48 (0.51 - 0.45) | -1.08(-1.21--0.95) |
| United States Virgin Islands | 0.07(0.05-0.11) | 0.18 (0.31 - 0.10) | 0.05(0.03-0.07) | 0.19 (0.34 - 0.10) | 1.85(1.76-1.94) |
| Uruguay | 0.68(0.57-0.82) | 0.06 (0.08 - 0.05) | 1.78(1.50-2.12) | 0.14 (0.19 - 0.11) | -1.01(-1.49--0.52) |
| Uzbekistan | 31.87(23.49-41.22) | 0.41 (0.54 - 0.30) | 65.72(49.39-84.67) | 0.45 (0.59 - 0.34) | 3.63(3.10-4.16) |
| Vanuatu | 0.33(0.17-0.62) | 0.64 (1.33 - 0.30) | 0.73(0.42-1.21) | 0.65 (1.17 - 0.32) | 0.07(-0.12-0.27) |
| Venezuela (Bolivarian Republic of) | 20.97(19.87-22.10) | 0.28 (0.31 - 0.26) | 34.17(26.44-43.49) | 0.35 (0.45 - 0.27) | -0.04(-0.19-0.10) |
| Viet Nam | 415.82(293.19-581.28) | 1.69 (2.73 - 1.00) | 533.21(347.46-860.76) | 1.23 (2.20 - 0.66) | 0.64(-0.15-1.44) |
| Yemen | 9.39(3.12-21.32) | 0.24 (0.57 - 0.08) | 21.30(9.20-45.54) | 0.17 (0.38 - 0.07) | -1.23(-1.43--1.03) |
| Zambia | 31.77(20.69-50.45) | 1.32 (2.37 - 0.73) | 40.81(14.32-113.64) | 0.58 (1.73 - 0.18) | -1.24(-1.36--1.12) |
| Zimbabwe | 43.39(24.48-68.35) | 1.36 (2.34 - 0.71) | 129.64(76.03-202.98) | 2.29 (3.91 - 1.21) | -3.95(-4.69--3.21) |

**Table S2 incident cases of AYA liver cancer in 2021, and the age-standardized rates by sex and global、GBD region.**

| **regions** | **Incidence** | | | | | |
| --- | --- | --- | --- | --- | --- | --- |
|  | **both sexes** | | **female** | | **male** | |
|  | **cases** | **ASR per 100,000** | **cases** | **ASR per 100,000** | **cases** | **ASR per 100,000** |
| Global | 24348.06(21491.13-28273.11) | 0.79 (0.93 to 0.69) | 5872.29(5288.76-6567.48) | 0.39 (0.45 to 0.34) | 18475.76(15857.68-22363.29) | 1.19 (1.45 to 1.01) |
| East Asia | 12148.88(9606.79-15553.45) | 2.08 (2.68 to 1.62) | 1606.63(1199.14-2103.08) | 0.59 (0.79 to 0.44) | 10542.25(8131.14-13911.03) | 3.47 (4.62 to 2.62) |
| Oceania | 24.36(15.35-43.98) | 0.46 (0.87 to 0.27) | 10.43(6.64-17.81) | 0.39 (0.75 to 0.23) | 13.93(7.93-30.45) | 0.53 (1.20 to 0.29) |
| Southeast Asia | 2189.89(1782.72-2922.32) | 0.77 (1.05 to 0.59) | 510.67(328.78-682.01) | 0.36 (0.50 to 0.23) | 1679.21(1310.83-2342.99) | 1.16 (1.68 to 0.86) |
| Central Asia | 226.71(187.40-268.43) | 0.57 (0.69 to 0.46) | 101.57(84.56-120.72) | 0.51 (0.63 to 0.42) | 125.14(100.88-151.83) | 0.63 (0.77 to 0.50) |
| Eastern Europe | 195.28(180.58-209.91) | 0.24 (0.26 to 0.22) | 68.58(60.79-77.13) | 0.18 (0.20 to 0.16) | 126.70(115.96-138.42) | 0.31 (0.34 to 0.28) |
| Central Europe | 67.10(59.89-75.04) | 0.17 (0.19 to 0.15) | 19.89(17.21-22.92) | 0.10 (0.12 to 0.09) | 47.21(42.00-53.66) | 0.23 (0.26 to 0.20) |
| High-income Asia Pacific | 399.11(326.51-493.50) | 0.67 (0.87 to 0.52) | 110.76(89.41-137.57) | 0.39 (0.53 to 0.29) | 288.36(220.06-382.44) | 0.93 (1.30 to 0.67) |
| High-income North America | 618.05(589.23-648.16) | 0.48 (0.50 to 0.45) | 212.24(198.89-224.11) | 0.33 (0.35 to 0.31) | 405.81(381.88-429.88) | 0.62 (0.67 to 0.58) |
| Australasia | 61.31(53.49-70.68) | 0.53 (0.66 to 0.43) | 19.39(16.50-22.82) | 0.34 (0.44 to 0.26) | 41.92(34.50-50.80) | 0.73 (0.96 to 0.54) |
| Caribbean | 34.51(27.45-42.37) | 0.19 (0.24 to 0.14) | 13.64(10.41-18.89) | 0.15 (0.21 to 0.11) | 20.86(16.95-25.57) | 0.23 (0.30 to 0.17) |
| Western Europe | 526.01(499.53-555.59) | 0.37 (0.40 to 0.34) | 176.80(165.74-189.28) | 0.25 (0.28 to 0.23) | 349.22(326.13-373.79) | 0.49 (0.54 to 0.44) |
| Southern Latin America | 29.60(25.88-33.37) | 0.11 (0.13 to 0.09) | 10.81(9.32-12.63) | 0.08 (0.10 to 0.06) | 18.79(15.47-22.46) | 0.14 (0.18 to 0.11) |
| Andean Latin America | 77.48(59.29-101.22) | 0.29 (0.41 to 0.20) | 30.86(21.94-42.98) | 0.23 (0.33 to 0.15) | 46.62(34.98-60.56) | 0.35 (0.52 to 0.23) |
| Tropical Latin America | 151.47(143.32-159.77) | 0.16 (0.17 to 0.15) | 58.85(55.25-61.87) | 0.12 (0.14 to 0.11) | 92.62(85.99-99.96) | 0.20 (0.22 to 0.18) |
| Central Latin America | 227.74(204.73-251.33) | 0.23 (0.25 to 0.20) | 97.82(84.61-111.67) | 0.19 (0.22 to 0.16) | 129.93(117.55-141.91) | 0.26 (0.29 to 0.24) |
| South Asia | 2503.08(2161.53-2999.50) | 0.33 (0.40 to 0.27) | 928.14(764.37-1150.14) | 0.24 (0.31 to 0.19) | 1574.94(1310.75-1927.05) | 0.41 (0.51 to 0.33) |
| North Africa and Middle East | 1041.47(885.34-1212.00) | 0.39 (0.48 to 0.32) | 479.13(398.88-579.00) | 0.38 (0.48 to 0.30) | 562.34(470.78-670.22) | 0.41 (0.52 to 0.32) |
| Southern Sub-Saharan Africa | 522.57(416.86-649.06) | 1.50 (1.93 to 1.16) | 179.43(137.44-228.53) | 1.03 (1.39 to 0.73) | 343.14(261.13-473.32) | 1.98 (2.79 to 1.43) |
| Western Sub-Saharan Africa | 2187.93(1677.07-2743.96) | 1.34 (1.74 to 1.00) | 703.01(511.30-919.58) | 0.80 (1.08 to 0.57) | 1484.93(1135.38-1900.44) | 1.94 (2.59 to 1.43) |
| Central Sub-Saharan Africa | 244.55(106.48-580.31) | 0.52 (1.24 to 0.21) | 111.28(48.33-255.06) | 0.46 (1.15 to 0.18) | 133.28(51.19-367.06) | 0.58 (1.67 to 0.21) |
| Eastern Sub-Saharan Africa | 870.94(630.92-1218.90) | 0.58 (0.82 to 0.41) | 422.36(318.20-552.53) | 0.52 (0.70 to 0.38) | 448.58(301.53-683.64) | 0.64 (1.01 to 0.41) |
| **SDI regions** |  |  |  |  |  |  |
| High SDI | 1911.47(1805.61-2035.15) | 0.48 (0.52 to 0.45) | 587.93(553.18-624.23) | 0.31 (0.34 to 0.29) | 1323.54(1219.16-1440.62) | 0.64 (0.71 to 0.58) |
| Low SDI | 2719.06(2136.52-3635.80) | 0.69 (0.95 to 0.54) | 1116.63(855.87-1470.98) | 0.54 (0.73 to 0.41) | 1602.43(1245.28-2305.08) | 0.85 (1.23 to 0.64) |
| Middle SDI | 10484.52(8891.99-12866.82) | 1.05 (1.31 to 0.87) | 1909.27(1623.83-2246.81) | 0.39 (0.47 to 0.33) | 8575.25(6992.83-10835.35) | 1.69 (2.19 to 1.35) |
| High-middle SDI | 5386.91(4295.19-6784.88) | 1.02 (1.30 to 0.80) | 827.21(659.56-1041.34) | 0.33 (0.43 to 0.26) | 4559.70(3547.25-5902.74) | 1.66 (2.20 to 1.26) |
| Low-middle SDI | 3837.57(3290.94-4538.98) | 0.50 (0.60 to 0.42) | 1428.33(1240.57-1642.98) | 0.37 (0.44 to 0.31) | 2409.24(2004.17-2942.67) | 0.63 (0.79 to 0.50) |

**Table S3. Mortality of liver cancer among AYA between 1990 and 2021 at national level.**

| Nations | Number of cases, 1990 | Age-standardized rate per 100,000 population, 1990 | Number of cases, 2021 | Age-standardized rate per 100,000 population, 2021 | Estimated annual percentage change, 1990–2021 |
| --- | --- | --- | --- | --- | --- |
| Afghanistan | 19.95(13.88-28.35) | 0.86 (1.41 - 0.49) | 68.95(48.43-98.94) | 0.69 (1.10 - 0.40) | -1.51(-1.83--1.18) |
| Albania | 11.81(8.69-15.35) | 0.87 (1.22 - 0.60) | 4.88(3.54-6.80) | 0.51 (0.79 - 0.32) | -2.36(-2.70--2.02) |
| Algeria | 13.36(9.93-17.76) | 0.16 (0.23 - 0.10) | 32.07(22.12-45.42) | 0.17 (0.27 - 0.11) | 0.12(-0.00-0.23) |
| American Samoa | 0.09(0.07-0.13) | 0.52 (0.84 - 0.31) | 0.16(0.11-0.23) | 0.97 (1.51 - 0.57) | 2.61(2.15-3.07) |
| Andorra | 0.17(0.11-0.24) | 0.62 (1.02 - 0.35) | 0.16(0.10-0.23) | 0.54 (0.90 - 0.29) | -0.49(-0.56--0.42) |
| Angola | 44.15(6.38-139.94) | 1.28 (4.32 - 0.17) | 84.62(19.75-257.09) | 0.80 (2.45 - 0.17) | -1.50(-1.66--1.35) |
| Antigua and Barbuda | 0.06(0.05-0.06) | 0.24 (0.28 - 0.20) | 0.05(0.04-0.06) | 0.13 (0.16 - 0.11) | -1.56(-2.07--1.05) |
| Argentina | 4.95(4.10-6.00) | 0.04 (0.05 - 0.03) | 15.48(13.26-17.84) | 0.09 (0.11 - 0.07) | 2.86(2.61-3.11) |
| Armenia | 6.40(5.32-7.88) | 0.45 (0.55 - 0.37) | 4.18(3.43-5.01) | 0.34 (0.41 - 0.28) | -1.28(-1.82--0.75) |
| Australia | 12.12(10.81-13.60) | 0.17 (0.21 - 0.14) | 31.01(26.93-36.25) | 0.32 (0.41 - 0.25) | 1.82(1.57-2.08) |
| Austria | 5.14(4.60-5.85) | 0.17 (0.21 - 0.14) | 6.20(5.34-7.06) | 0.20 (0.25 - 0.16) | 0.02(-0.19-0.22) |
| Azerbaijan | 16.08(9.17-25.85) | 0.55 (0.91 - 0.30) | 23.25(12.86-40.02) | 0.50 (0.89 - 0.26) | -1.42(-1.80--1.03) |
| Bahamas | 0.39(0.34-0.44) | 0.36 (0.42 - 0.31) | 0.55(0.43-0.69) | 0.35 (0.45 - 0.28) | -0.68(-1.04--0.31) |
| Bahrain | 0.76(0.59-1.07) | 0.28 (0.44 - 0.18) | 1.44(1.00-1.97) | 0.18 (0.30 - 0.11) | -2.08(-2.70--1.47) |
| Bangladesh | 83.82(58.95-121.94) | 0.23 (0.39 - 0.13) | 145.64(88.46-245.58) | 0.22 (0.42 - 0.11) | -0.19(-0.36--0.02) |
| Barbados | 0.21(0.19-0.24) | 0.20 (0.23 - 0.17) | 0.18(0.14-0.23) | 0.17 (0.22 - 0.13) | -1.00(-1.32--0.68) |
| Belarus | 7.93(6.63-9.64) | 0.19 (0.25 - 0.14) | 7.26(5.52-9.30) | 0.21 (0.28 - 0.15) | -0.39(-0.84-0.07) |
| Belgium | 5.48(4.84-6.16) | 0.14 (0.17 - 0.11) | 6.27(5.38-7.29) | 0.16 (0.21 - 0.13) | 0.36(0.14-0.58) |
| Belize | 0.10(0.08-0.11) | 0.15 (0.18 - 0.13) | 0.45(0.39-0.51) | 0.26 (0.30 - 0.22) | 0.84(0.30-1.39) |
| Benin | 41.82(21.16-66.97) | 2.80 (5.17 - 1.25) | 88.38(56.34-125.93) | 1.97 (3.35 - 1.05) | -1.53(-1.82--1.25) |
| Bermuda | 0.05(0.05-0.06) | 0.19 (0.22 - 0.16) | 0.02(0.01-0.02) | 0.09 (0.12 - 0.07) | -2.50(-2.99--2.00) |
| Bhutan | 0.79(0.44-1.27) | 0.36 (0.67 - 0.17) | 1.29(0.75-2.09) | 0.37 (0.66 - 0.19) | -0.17(-0.39-0.06) |
| Bolivia (Plurinational State of) | 7.30(4.86-10.14) | 0.32 (0.52 - 0.19) | 11.70(7.43-18.90) | 0.24 (0.42 - 0.14) | -1.35(-1.62--1.08) |
| Bosnia and Herzegovina | 9.02(7.55-10.78) | 0.47 (0.60 - 0.35) | 2.40(1.67-3.14) | 0.22 (0.30 - 0.14) | -3.49(-4.08--2.90) |
| Botswana | 3.45(1.45-7.24) | 0.81 (1.84 - 0.30) | 10.12(4.26-24.21) | 0.91 (2.34 - 0.34) | -0.77(-1.34--0.20) |
| Brazil | 99.94(95.98-104.20) | 0.17 (0.18 - 0.16) | 127.15(120.37-133.04) | 0.14 (0.15 - 0.13) | -0.07(-0.31-0.17) |
| Brunei Darussalam | 1.03(0.75-1.46) | 0.85 (1.32 - 0.52) | 1.22(0.89-1.68) | 0.54 (0.83 - 0.34) | -1.87(-2.06--1.67) |
| Bulgaria | 17.27(13.19-21.61) | 0.55 (0.70 - 0.41) | 4.93(3.59-6.80) | 0.22 (0.31 - 0.16) | -2.49(-2.84--2.14) |
| Burkina Faso | 93.13(39.18-210.13) | 3.47 (8.34 - 1.33) | 212.88(90.54-411.42) | 2.83 (5.76 - 1.12) | -0.69(-0.93--0.46) |
| Burundi | 10.72(6.57-19.81) | 0.57 (1.12 - 0.29) | 16.36(9.88-26.44) | 0.33 (0.62 - 0.17) | -1.98(-2.12--1.84) |
| Cabo Verde | 2.62(1.83-3.69) | 2.49 (4.18 - 1.42) | 5.06(3.52-7.12) | 1.95 (3.34 - 1.08) | -0.87(-1.21--0.52) |
| Cambodia | 31.47(16.96-56.30) | 0.95 (1.85 - 0.45) | 46.59(21.98-93.15) | 0.64 (1.36 - 0.28) | -1.61(-1.72--1.49) |
| Cameroon | 89.27(61.86-134.94) | 2.76 (4.66 - 1.59) | 233.97(130.89-409.20) | 2.05 (4.02 - 1.00) | -1.29(-1.45--1.13) |
| Canada | 18.72(16.58-21.23) | 0.16 (0.19 - 0.13) | 32.50(27.85-37.61) | 0.25 (0.31 - 0.20) | 1.81(1.51-2.10) |
| Central African Republic | 10.34(3.82-21.75) | 1.16 (2.70 - 0.41) | 13.74(5.34-30.51) | 0.72 (1.68 - 0.26) | -2.24(-2.48--1.99) |
| Chad | 40.16(15.45-87.16) | 2.23 (5.03 - 0.78) | 98.84(53.17-187.59) | 1.93 (3.79 - 0.92) | -0.69(-0.97--0.42) |
| Chile | 3.57(2.84-4.44) | 0.07 (0.09 - 0.05) | 7.44(6.34-8.90) | 0.10 (0.13 - 0.08) | 1.79(1.49-2.08) |
| China | 10930.59(9096.96-13076.34) | 2.16 (2.63 - 1.78) | 8653.20(6797.85-11127.81) | 1.54 (1.99 - 1.19) | -1.76(-2.15--1.38) |
| Colombia | 31.08(28.23-34.12) | 0.24 (0.29 - 0.20) | 30.88(25.62-37.22) | 0.15 (0.20 - 0.12) | -1.68(-2.27--1.09) |
| Comoros | 0.90(0.40-1.39) | 0.61 (1.08 - 0.27) | 1.62(1.09-2.48) | 0.55 (0.92 - 0.30) | -1.11(-1.73--0.48) |
| Congo | 10.19(4.33-20.32) | 1.31 (2.86 - 0.50) | 15.79(6.59-33.14) | 0.75 (1.73 - 0.28) | -2.28(-2.57--1.98) |
| Cook Islands | 0.08(0.06-0.12) | 1.15 (1.85 - 0.68) | 0.06(0.04-0.09) | 1.15 (1.90 - 0.64) | 0.42(0.24-0.59) |
| Costa Rica | 4.18(3.66-4.72) | 0.35 (0.43 - 0.28) | 6.71(5.56-8.09) | 0.34 (0.43 - 0.26) | -0.58(-1.20-0.05) |
| Croatia | 4.31(3.29-5.46) | 0.22 (0.28 - 0.17) | 1.35(1.01-1.78) | 0.10 (0.13 - 0.07) | -3.05(-3.60--2.49) |
| Cuba | 8.66(7.44-10.20) | 0.20 (0.25 - 0.15) | 3.86(3.04-4.75) | 0.10 (0.13 - 0.07) | -2.69(-3.23--2.16) |
| Cyprus | 0.45(0.32-0.66) | 0.14 (0.24 - 0.09) | 0.63(0.45-0.88) | 0.10 (0.17 - 0.06) | -1.92(-2.42--1.42) |
| Czechia | 12.13(10.46-14.11) | 0.30 (0.37 - 0.24) | 3.22(2.44-4.05) | 0.10 (0.13 - 0.07) | -3.94(-4.37--3.51) |
| C么te d'Ivoire | 32.18(19.18-50.78) | 0.78 (1.42 - 0.40) | 49.93(29.03-77.80) | 0.48 (0.83 - 0.24) | -1.98(-2.19--1.77) |
| Democratic People's Republic of Korea | 155.50(75.74-276.36) | 2.03 (3.97 - 0.89) | 147.09(77.10-273.39) | 1.36 (2.67 - 0.66) | -1.74(-1.90--1.58) |
| Democratic Republic of the Congo | 54.43(26.79-117.63) | 0.44 (0.96 - 0.19) | 103.03(47.94-236.38) | 0.33 (0.79 - 0.14) | -1.20(-1.38--1.02) |
| Denmark | 1.96(1.72-2.21) | 0.10 (0.12 - 0.08) | 1.16(1.00-1.36) | 0.06 (0.08 - 0.05) | -2.05(-2.53--1.57) |
| Djibouti | 0.60(0.32-1.05) | 0.41 (0.76 - 0.20) | 2.37(1.26-4.08) | 0.43 (0.79 - 0.21) | 0.41(0.00-0.81) |
| Dominica | 0.04(0.03-0.07) | 0.17 (0.33 - 0.09) | 0.06(0.04-0.08) | 0.21 (0.35 - 0.12) | -0.04(-0.59-0.52) |
| Dominican Republic | 4.36(3.17-5.82) | 0.16 (0.25 - 0.10) | 10.17(6.96-14.13) | 0.23 (0.36 - 0.13) | 1.10(0.86-1.34) |
| Ecuador | 20.44(17.86-23.09) | 0.54 (0.68 - 0.43) | 20.29(15.68-25.79) | 0.28 (0.38 - 0.20) | -2.87(-3.70--2.02) |
| Egypt | 175.45(125.25-252.76) | 0.89 (1.43 - 0.55) | 359.08(272.73-469.08) | 0.86 (1.23 - 0.58) | 0.13(0.00-0.26) |
| El Salvador | 2.71(2.34-3.18) | 0.15 (0.19 - 0.11) | 3.39(2.66-4.25) | 0.14 (0.19 - 0.10) | 0.01(-0.38-0.39) |
| Equatorial Guinea | 0.50(0.25-1.08) | 0.38 (0.87 - 0.17) | 3.34(1.58-6.07) | 0.56 (1.09 - 0.24) | 1.25(0.92-1.59) |
| Eritrea | 4.69(2.81-7.92) | 0.43 (0.81 - 0.23) | 10.84(6.15-19.64) | 0.42 (0.81 - 0.20) | -0.73(-1.02--0.44) |
| Estonia | 1.83(1.53-2.17) | 0.30 (0.39 - 0.23) | 0.91(0.75-1.08) | 0.20 (0.27 - 0.15) | -2.07(-2.38--1.76) |
| Eswatini | 2.96(1.13-5.75) | 1.23 (2.59 - 0.42) | 17.62(5.20-45.20) | 3.77 (10.72 - 1.01) | 3.87(2.26-5.49) |
| Ethiopia | 66.73(42.95-99.86) | 0.41 (0.62 - 0.25) | 107.05(71.76-159.57) | 0.26 (0.41 - 0.17) | -1.99(-2.21--1.76) |
| Fiji | 1.57(1.07-2.28) | 0.53 (0.87 - 0.30) | 2.06(1.31-3.05) | 0.57 (0.94 - 0.32) | 0.66(0.21-1.11) |
| Finland | 4.66(3.95-5.54) | 0.23 (0.29 - 0.18) | 2.94(2.47-3.53) | 0.16 (0.20 - 0.13) | -1.08(-1.36--0.79) |
| France | 56.50(50.94-62.22) | 0.25 (0.30 - 0.20) | 55.12(47.86-63.82) | 0.26 (0.34 - 0.20) | 0.26(0.06-0.47) |
| Gabon | 3.47(1.37-7.96) | 1.04 (2.57 - 0.37) | 6.20(3.22-11.07) | 0.92 (1.83 - 0.39) | -0.69(-0.95--0.44) |
| Gambia | 12.27(8.14-17.58) | 3.90 (6.44 - 2.22) | 41.59(25.62-67.73) | 4.83 (8.95 - 2.47) | 0.24(-0.06-0.55) |
| Georgia | 8.43(7.06-9.86) | 0.39 (0.46 - 0.33) | 2.61(2.22-3.03) | 0.20 (0.24 - 0.17) | -2.68(-3.24--2.13) |
| Germany | 39.50(34.39-45.28) | 0.13 (0.16 - 0.10) | 49.27(42.38-56.70) | 0.17 (0.22 - 0.14) | 0.93(0.80-1.06) |
| Ghana | 138.37(81.91-222.49) | 2.71 (4.82 - 1.41) | 243.12(150.42-396.99) | 1.80 (3.31 - 0.92) | -1.69(-2.02--1.36) |
| Greece | 8.08(7.52-8.66) | 0.21 (0.24 - 0.19) | 10.53(9.62-11.56) | 0.34 (0.38 - 0.30) | -1.45(-1.98--0.92) |
| Greenland | 0.15(0.11-0.21) | 0.56 (0.86 - 0.34) | 0.07(0.04-0.11) | 0.33 (0.53 - 0.19) | 1.23(1.04-1.43) |
| Grenada | 0.07(0.06-0.09) | 0.24 (0.33 - 0.17) | 0.10(0.09-0.13) | 0.27 (0.37 - 0.20) | -1.25(-1.66--0.84) |
| Guam | 0.21(0.17-0.26) | 0.34 (0.46 - 0.25) | 0.52(0.40-0.65) | 0.96 (1.32 - 0.66) | 0.01(-0.42-0.44) |
| Guatemala | 15.66(14.70-16.60) | 0.60 (0.66 - 0.55) | 27.56(23.75-31.26) | 0.44 (0.52 - 0.37) | 3.97(3.74-4.20) |
| Guinea | 63.07(44.55-87.54) | 3.37 (5.25 - 1.99) | 127.41(81.51-190.65) | 2.82 (4.80 - 1.56) | -2.01(-3.00--1.00) |
| Guinea-Bissau | 16.12(6.65-25.91) | 5.10 (9.22 - 1.90) | 25.55(15.09-38.31) | 3.38 (5.97 - 1.70) | -0.42(-0.61--0.23) |
| Guyana | 0.61(0.50-0.75) | 0.20 (0.25 - 0.16) | 0.76(0.56-0.96) | 0.26 (0.35 - 0.19) | -1.60(-1.74--1.46) |
| Haiti | 3.54(2.03-6.27) | 0.16 (0.30 - 0.08) | 6.37(3.11-12.20) | 0.12 (0.24 - 0.05) | 0.45(-0.06-0.96) |
| Honduras | 2.66(1.92-3.71) | 0.18 (0.27 - 0.10) | 5.43(3.03-8.58) | 0.14 (0.24 - 0.07) | -0.85(-0.97--0.73) |
| Hungary | 11.47(9.08-14.64) | 0.28 (0.38 - 0.21) | 2.69(2.03-3.45) | 0.09 (0.12 - 0.06) | -1.36(-1.78--0.95) |
| Iceland | 0.13(0.12-0.15) | 0.13 (0.15 - 0.10) | 0.19(0.16-0.23) | 0.15 (0.19 - 0.11) | -4.29(-5.01--3.57) |
| India | 691.80(613.55-812.58) | 0.22 (0.27 - 0.19) | 1640.57(1387.29-1999.98) | 0.27 (0.34 - 0.23) | 0.09(-0.18-0.37) |
| Indonesia | 288.68(186.62-422.65) | 0.41 (0.63 - 0.27) | 486.29(309.26-773.33) | 0.41 (0.66 - 0.26) | 0.58(0.34-0.81) |
| Iran (Islamic Republic of) | 41.76(35.52-51.25) | 0.22 (0.27 - 0.18) | 105.41(92.50-118.85) | 0.26 (0.31 - 0.22) | 0.02(-0.06-0.11) |
| Iraq | 22.02(15.80-30.58) | 0.36 (0.57 - 0.22) | 49.51(34.27-75.13) | 0.30 (0.48 - 0.19) | 0.26(-0.40-0.92) |
| Ireland | 1.28(1.15-1.44) | 0.10 (0.12 - 0.08) | 1.86(1.60-2.19) | 0.11 (0.14 - 0.08) | -0.33(-0.57--0.09) |
| Israel | 1.55(1.36-1.76) | 0.08 (0.10 - 0.06) | 3.34(2.86-3.90) | 0.10 (0.13 - 0.08) | -0.02(-0.58-0.54) |
| Italy | 51.83(49.44-54.14) | 0.24 (0.26 - 0.23) | 25.90(24.31-27.57) | 0.15 (0.16 - 0.14) | 0.40(-0.07-0.86) |
| Jamaica | 0.55(0.44-0.68) | 0.06 (0.09 - 0.05) | 1.42(1.01-1.96) | 0.12 (0.17 - 0.08) | -2.04(-2.41--1.66) |
| Japan | 192.25(187.47-197.02) | 0.40 (0.42 - 0.39) | 59.75(58.08-61.75) | 0.16 (0.17 - 0.16) | 1.08(0.29-1.87) |
| Jordan | 1.66(1.11-2.58) | 0.14 (0.23 - 0.08) | 4.33(2.98-6.06) | 0.08 (0.14 - 0.05) | -3.57(-4.07--3.08) |
| Kazakhstan | 60.29(53.91-67.29) | 0.90 (1.01 - 0.80) | 27.46(22.67-33.58) | 0.36 (0.44 - 0.29) | -2.09(-2.37--1.80) |
| Kenya | 20.30(14.59-29.99) | 0.28 (0.43 - 0.19) | 80.48(57.46-117.43) | 0.42 (0.61 - 0.29) | -3.95(-4.52--3.37) |
| Kiribati | 0.32(0.24-0.45) | 1.18 (1.82 - 0.72) | 0.55(0.35-0.82) | 1.14 (1.88 - 0.64) | 1.34(1.04-1.64) |
| Kuwait | 2.42(2.07-2.88) | 0.27 (0.35 - 0.21) | 1.27(1.03-1.59) | 0.05 (0.06 - 0.04) | -0.42(-0.71--0.14) |
| Kyrgyzstan | 11.29(8.44-14.94) | 0.68 (0.93 - 0.50) | 4.47(3.03-6.16) | 0.16 (0.22 - 0.11) | -5.07(-6.09--4.04) |
| Lao People's Democratic Republic | 16.09(10.28-22.87) | 1.21 (2.02 - 0.65) | 24.17(15.28-37.00) | 0.78 (1.36 - 0.42) | -4.01(-4.68--3.34) |
| Latvia | 2.54(2.09-3.02) | 0.25 (0.33 - 0.19) | 1.33(1.05-1.68) | 0.22 (0.30 - 0.16) | -1.82(-1.98--1.67) |
| Lebanon | 2.65(1.88-3.66) | 0.25 (0.39 - 0.16) | 3.67(2.78-4.90) | 0.15 (0.22 - 0.09) | -1.45(-1.82--1.07) |
| Lesotho | 3.55(1.14-9.24) | 0.74 (2.01 - 0.20) | 20.66(6.76-60.90) | 2.80 (9.01 - 0.79) | -1.88(-2.12--1.63) |
| Liberia | 22.11(10.58-36.05) | 2.67 (4.94 - 1.16) | 54.49(30.68-84.43) | 2.68 (4.81 - 1.37) | 4.55(3.49-5.62) |
| Libya | 7.51(5.15-10.33) | 0.54 (0.87 - 0.31) | 21.90(14.88-31.65) | 0.68 (1.09 - 0.39) | -0.21(-0.52-0.10) |
| Lithuania | 2.97(2.56-3.41) | 0.21 (0.26 - 0.17) | 1.97(1.58-2.52) | 0.23 (0.30 - 0.17) | 0.99(0.75-1.23) |
| Luxembourg | 0.28(0.27-0.30) | 0.18 (0.20 - 0.16) | 0.34(0.31-0.38) | 0.14 (0.15 - 0.12) | -0.06(-0.40-0.29) |
| Madagascar | 20.55(14.19-29.38) | 0.52 (0.82 - 0.30) | 38.33(25.29-60.04) | 0.37 (0.65 - 0.20) | -1.41(-1.71--1.11) |
| Malawi | 13.69(9.11-20.49) | 0.43 (0.73 - 0.24) | 39.37(25.98-58.42) | 0.57 (0.99 - 0.31) | -1.12(-1.30--0.94) |
| Malaysia | 26.19(19.90-35.37) | 0.38 (0.60 - 0.22) | 65.93(48.50-91.84) | 0.46 (0.76 - 0.27) | 0.35(-0.26-0.96) |
| Maldives | 0.31(0.17-0.49) | 0.51 (0.94 - 0.22) | 0.93(0.56-1.43) | 0.28 (0.49 - 0.14) | 0.33(0.06-0.61) |
| Mali | 78.38(60.28-99.65) | 3.00 (4.42 - 1.97) | 230.74(160.73-314.77) | 3.18 (5.20 - 1.83) | -2.79(-3.44--2.13) |
| Malta | 0.11(0.10-0.13) | 0.08 (0.10 - 0.06) | 0.19(0.16-0.23) | 0.13 (0.17 - 0.10) | 0.29(0.18-0.40) |
| Marshall Islands | 0.07(0.04-0.10) | 0.45 (0.83 - 0.23) | 0.14(0.09-0.25) | 0.62 (1.16 - 0.32) | 1.31(0.98-1.64) |
| Mauritania | 35.73(7.32-74.52) | 5.33 (12.57 - 0.99) | 38.25(18.60-64.49) | 2.60 (5.00 - 1.10) | 0.79(0.53-1.06) |
| Mauritius | 1.52(1.40-1.66) | 0.31 (0.35 - 0.28) | 0.25(0.22-0.27) | 0.05 (0.06 - 0.05) | -2.66(-2.78--2.53) |
| Mexico | 42.75(41.89-43.66) | 0.14 (0.14 - 0.13) | 87.86(78.97-96.91) | 0.17 (0.19 - 0.15) | 0.40(-1.54-2.38) |
| Micronesia (Federated States of) | 0.27(0.18-0.41) | 0.76 (1.29 - 0.42) | 0.29(0.17-0.52) | 0.77 (1.45 - 0.39) | 1.12(0.96-1.28) |
| Monaco | 0.02(0.02-0.04) | 0.25 (0.39 - 0.15) | 0.05(0.03-0.08) | 0.52 (0.88 - 0.27) | -0.10(-0.35-0.14) |
| Mongolia | 36.82(23.95-54.17) | 4.87 (7.80 - 2.79) | 58.29(40.30-83.31) | 4.24 (6.43 - 2.75) | 2.25(1.64-2.86) |
| Montenegro | 1.13(0.90-1.42) | 0.45 (0.58 - 0.34) | 0.77(0.58-1.03) | 0.35 (0.47 - 0.26) | -0.73(-1.07--0.39) |
| Morocco | 3.61(2.57-4.95) | 0.04 (0.06 - 0.02) | 6.44(4.30-10.30) | 0.04 (0.08 - 0.02) | -0.86(-1.07--0.64) |
| Mozambique | 35.53(19.40-56.33) | 0.85 (1.52 - 0.41) | 124.37(56.95-259.34) | 1.29 (2.97 - 0.51) | 0.18(0.11-0.25) |
| Myanmar | 68.07(28.70-132.30) | 0.45 (0.95 - 0.17) | 78.99(38.75-157.08) | 0.36 (0.79 - 0.16) | 1.51(1.12-1.89) |
| Namibia | 1.04(0.57-1.87) | 0.22 (0.43 - 0.10) | 3.32(1.97-5.33) | 0.34 (0.64 - 0.17) | -0.86(-0.93--0.78) |
| Nauru | 0.04(0.03-0.06) | 1.19 (2.00 - 0.66) | 0.05(0.02-0.08) | 1.09 (1.98 - 0.50) | 0.92(0.08-1.77) |
| Nepal | 12.45(8.32-18.19) | 0.19 (0.32 - 0.10) | 35.54(23.19-51.43) | 0.29 (0.48 - 0.16) | -0.58(-0.73--0.42) |
| Netherlands | 5.34(4.62-6.29) | 0.09 (0.11 - 0.07) | 6.03(5.00-7.21) | 0.11 (0.14 - 0.09) | 1.63(1.42-1.84) |
| New Zealand | 2.98(2.53-3.52) | 0.21 (0.26 - 0.17) | 6.63(5.54-7.99) | 0.35 (0.45 - 0.28) | 0.60(0.33-0.88) |
| Nicaragua | 2.90(2.36-3.60) | 0.23 (0.33 - 0.16) | 5.99(4.49-7.76) | 0.22 (0.33 - 0.14) | 1.41(1.14-1.67) |
| Niger | 58.34(25.67-116.14) | 2.47 (5.52 - 0.96) | 88.64(52.78-156.91) | 1.26 (2.44 - 0.64) | -0.01(-0.42-0.39) |
| Nigeria | 157.25(65.82-307.10) | 0.54 (1.07 - 0.23) | 315.86(178.62-520.24) | 0.42 (0.68 - 0.24) | -2.35(-2.48--2.22) |
| Niue | 0.00(0.00-0.01) | 0.56 (1.01 - 0.29) | 0.00(0.00-0.01) | 0.58 (1.06 - 0.30) | -1.20(-1.41--0.98) |
| North Macedonia | 4.92(4.17-5.95) | 0.62 (0.82 - 0.47) | 2.89(2.18-3.66) | 0.33 (0.47 - 0.23) | -0.19(-0.44-0.06) |
| Northern Mariana Islands | 0.14(0.09-0.22) | 0.58 (0.99 - 0.32) | 0.06(0.04-0.08) | 0.34 (0.54 - 0.20) | -2.47(-2.82--2.12) |
| Norway | 2.19(2.07-2.32) | 0.13 (0.14 - 0.12) | 5.19(4.78-5.70) | 0.27 (0.31 - 0.25) | -1.61(-2.08--1.14) |
| Oman | 1.55(0.93-2.65) | 0.18 (0.35 - 0.09) | 6.66(4.42-9.52) | 0.24 (0.40 - 0.14) | 2.08(1.55-2.61) |
| Pakistan | 125.65(94.09-167.12) | 0.36 (0.51 - 0.24) | 442.31(328.75-596.44) | 0.48 (0.68 - 0.32) | 1.02(0.73-1.31) |
| Palau | 0.07(0.04-0.12) | 1.04 (2.11 - 0.44) | 0.10(0.06-0.16) | 1.54 (2.82 - 0.77) | 0.72(0.39-1.04) |
| Palestine | 2.59(1.77-3.70) | 0.41 (0.66 - 0.25) | 5.68(4.40-7.24) | 0.29 (0.42 - 0.19) | 1.35(1.07-1.63) |
| Panama | 1.86(1.74-1.96) | 0.20 (0.22 - 0.18) | 3.43(2.85-4.09) | 0.21 (0.26 - 0.17) | -1.41(-1.74--1.08) |
| Papua New Guinea | 6.79(2.66-16.65) | 0.47 (1.23 - 0.16) | 13.33(6.24-29.77) | 0.33 (0.80 - 0.14) | 0.55(0.07-1.04) |
| Paraguay | 2.28(1.81-2.80) | 0.16 (0.22 - 0.11) | 6.91(4.75-9.65) | 0.23 (0.35 - 0.13) | -1.44(-1.60--1.28) |
| Peru | 21.22(15.09-30.53) | 0.26 (0.42 - 0.16) | 36.71(24.43-53.32) | 0.25 (0.40 - 0.14) | 1.63(1.42-1.84) |
| Philippines | 336.74(245.39-394.95) | 1.51 (1.81 - 1.09) | 371.89(307.31-445.71) | 0.84 (1.02 - 0.69) | -0.34(-0.56--0.12) |
| Poland | 7.31(6.67-8.18) | 0.05 (0.05 - 0.04) | 14.26(12.92-15.78) | 0.10 (0.11 - 0.09) | -2.18(-2.32--2.05) |
| Portugal | 7.12(6.11-8.27) | 0.19 (0.24 - 0.15) | 5.16(4.35-6.34) | 0.16 (0.21 - 0.12) | 3.39(2.46-4.34) |
| Puerto Rico | 3.97(3.29-4.82) | 0.29 (0.39 - 0.21) | 3.18(2.54-3.91) | 0.30 (0.40 - 0.21) | -1.33(-1.72--0.93) |
| Qatar | 1.08(0.75-1.68) | 0.40 (0.69 - 0.24) | 8.34(5.76-11.41) | 0.43 (0.71 - 0.25) | -1.14(-1.97--0.30) |
| Republic of Korea | 502.16(340.76-706.16) | 2.55 (4.04 - 1.50) | 140.54(105.59-193.39) | 0.74 (1.08 - 0.50) | -0.63(-1.06--0.21) |
| Republic of Moldova | 4.81(4.50-5.18) | 0.26 (0.29 - 0.24) | 3.62(3.15-4.08) | 0.25 (0.29 - 0.21) | -4.66(-4.89--4.43) |
| Romania | 12.71(10.12-15.60) | 0.14 (0.19 - 0.10) | 8.25(6.53-10.40) | 0.14 (0.19 - 0.10) | -0.33(-0.68-0.02) |
| Russian Federation | 105.48(102.07-108.66) | 0.17 (0.18 - 0.16) | 128.55(119.72-136.96) | 0.23 (0.25 - 0.21) | -0.27(-0.58-0.05) |
| Rwanda | 20.89(13.59-30.05) | 0.85 (1.38 - 0.46) | 25.06(15.51-40.16) | 0.48 (0.85 - 0.25) | 1.46(0.76-2.17) |
| Saint Kitts and Nevis | 0.06(0.05-0.07) | 0.37 (0.44 - 0.32) | 0.03(0.02-0.05) | 0.14 (0.20 - 0.10) | -2.68(-3.18--2.19) |
| Saint Lucia | 0.10(0.08-0.12) | 0.21 (0.25 - 0.18) | 0.12(0.10-0.15) | 0.17 (0.21 - 0.14) | -2.78(-3.87--1.68) |
| Saint Vincent and the Grenadines | 0.13(0.11-0.15) | 0.33 (0.39 - 0.28) | 0.12(0.10-0.14) | 0.28 (0.33 - 0.24) | -1.20(-1.55--0.85) |
| Samoa | 0.24(0.15-0.37) | 0.44 (0.77 - 0.24) | 0.33(0.18-0.53) | 0.45 (0.81 - 0.22) | -1.02(-1.33--0.70) |
| San Marino | 0.01(0.01-0.01) | 0.09 (0.14 - 0.06) | 0.01(0.01-0.02) | 0.13 (0.23 - 0.07) | -0.25(-0.42--0.08) |
| Sao Tome and Principe | 0.19(0.12-0.28) | 0.58 (1.01 - 0.30) | 0.37(0.19-0.74) | 0.44 (0.94 - 0.19) | 1.57(1.44-1.70) |
| Saudi Arabia | 24.02(15.03-37.62) | 0.40 (0.69 - 0.22) | 53.54(33.94-77.67) | 0.25 (0.40 - 0.14) | -1.11(-1.56--0.66) |
| Senegal | 48.22(27.04-73.38) | 2.08 (3.61 - 1.06) | 75.84(47.96-112.32) | 1.35 (2.23 - 0.74) | -2.25(-2.64--1.86) |
| Serbia | 12.72(9.25-17.18) | 0.34 (0.52 - 0.21) | 6.98(4.80-10.15) | 0.21 (0.34 - 0.12) | -1.42(-1.58--1.25) |
| Seychelles | 0.23(0.16-0.30) | 0.86 (1.36 - 0.51) | 0.14(0.10-0.20) | 0.32 (0.54 - 0.17) | -1.80(-1.91--1.69) |
| Sierra Leone | 32.93(10.37-63.12) | 2.31 (5.01 - 0.66) | 48.02(30.18-74.51) | 1.48 (2.65 - 0.77) | -2.79(-3.24--2.34) |
| Singapore | 9.00(7.74-10.46) | 0.33 (0.49 - 0.19) | 4.25(3.47-5.20) | 0.05 (0.07 - 0.03) | -1.41(-1.66--1.16) |
| Slovakia | 8.29(5.86-11.57) | 0.38 (0.54 - 0.26) | 4.13(2.56-6.34) | 0.21 (0.32 - 0.13) | -3.32(-5.75--0.84) |
| Slovenia | 2.98(2.64-3.41) | 0.36 (0.45 - 0.29) | 0.88(0.72-1.08) | 0.13 (0.18 - 0.10) | -2.23(-2.55--1.90) |
| Solomon Islands | 0.84(0.25-1.81) | 0.83 (1.92 - 0.22) | 1.88(1.13-2.95) | 0.76 (1.37 - 0.39) | -3.81(-4.17--3.45) |
| Somalia | 21.78(9.24-39.77) | 0.83 (1.73 - 0.33) | 55.63(24.88-107.67) | 0.79 (1.65 - 0.31) | -0.35(-0.63--0.08) |
| South Africa | 182.15(117.85-273.81) | 1.33 (1.97 - 0.84) | 300.28(247.80-360.43) | 1.15 (1.44 - 0.93) | -0.39(-0.65--0.14) |
| South Sudan | 10.26(6.22-16.29) | 0.54 (0.98 - 0.27) | 20.60(12.97-32.20) | 0.66 (1.16 - 0.34) | -1.47(-2.68--0.24) |
| Spain | 40.40(36.20-44.87) | 0.28 (0.34 - 0.23) | 28.25(24.16-33.38) | 0.20 (0.26 - 0.16) | 0.61(0.32-0.89) |
| Sri Lanka | 13.68(10.15-18.35) | 0.19 (0.31 - 0.12) | 10.35(6.34-15.60) | 0.13 (0.21 - 0.07) | -1.42(-1.62--1.22) |
| Sudan | 22.46(12.56-39.58) | 0.34 (0.64 - 0.17) | 54.65(32.79-86.06) | 0.32 (0.56 - 0.17) | -2.43(-3.00--1.86) |
| Suriname | 0.31(0.20-0.44) | 0.22 (0.37 - 0.13) | 0.55(0.37-0.80) | 0.25 (0.43 - 0.14) | 0.02(-0.14-0.19) |
| Sweden | 5.95(5.43-6.52) | 0.20 (0.23 - 0.17) | 6.57(5.72-7.71) | 0.19 (0.23 - 0.15) | 0.04(-0.26-0.34) |
| Switzerland | 7.45(6.34-8.78) | 0.27 (0.34 - 0.21) | 3.33(2.80-4.01) | 0.11 (0.14 - 0.08) | 1.02(-0.04-2.09) |
| Syrian Arab Republic | 25.46(19.60-33.51) | 0.64 (0.89 - 0.45) | 15.91(11.15-22.52) | 0.34 (0.51 - 0.22) | -3.29(-3.51--3.08) |
| Taiwan (Province of China) | 149.32(134.06-164.00) | 1.58 (1.86 - 1.33) | 83.57(69.32-100.75) | 0.93 (1.19 - 0.71) | -2.53(-3.15--1.89) |
| Tajikistan | 11.98(7.15-18.82) | 0.65 (1.06 - 0.37) | 16.68(10.20-25.45) | 0.41 (0.65 - 0.23) | -1.31(-2.18--0.43) |
| Thailand | 322.70(224.37-465.39) | 1.35 (2.14 - 0.83) | 361.60(238.10-521.13) | 1.53 (2.48 - 0.89) | -2.45(-2.82--2.08) |
| Timor-Leste | 0.98(0.55-1.66) | 0.34 (0.61 - 0.17) | 1.21(0.65-2.29) | 0.26 (0.50 - 0.13) | -0.52(-0.97--0.07) |
| Togo | 15.63(10.73-23.88) | 1.36 (2.32 - 0.81) | 35.31(21.69-61.03) | 1.15 (2.11 - 0.64) | -1.00(-1.51--0.49) |
| Tokelau | 0.00(0.00-0.01) | 0.52 (1.03 - 0.25) | 0.00(0.00-0.00) | 0.64 (1.15 - 0.34) | -0.82(-1.03--0.61) |
| Tonga | 0.64(0.42-0.99) | 2.30 (3.83 - 1.32) | 0.86(0.51-1.44) | 2.52 (4.64 - 1.35) | 0.62(0.39-0.84) |
| Trinidad and Tobago | 0.94(0.84-1.05) | 0.20 (0.22 - 0.17) | 1.32(1.00-1.68) | 0.23 (0.30 - 0.17) | 0.02(-0.37-0.42) |
| Tunisia | 4.12(2.97-5.59) | 0.14 (0.22 - 0.08) | 7.81(5.08-11.28) | 0.16 (0.27 - 0.09) | 0.06(-0.26-0.39) |
| Turkey | 43.71(33.04-57.43) | 0.20 (0.31 - 0.13) | 52.91(39.83-69.96) | 0.16 (0.24 - 0.10) | 0.40(0.33-0.46) |
| Turkmenistan | 7.39(6.62-8.18) | 0.53 (0.59 - 0.46) | 9.93(7.41-13.07) | 0.48 (0.63 - 0.36) | -1.13(-1.69--0.56) |
| Tuvalu | 0.02(0.01-0.03) | 0.62 (1.03 - 0.36) | 0.03(0.02-0.05) | 0.67 (1.20 - 0.36) | -0.02(-0.25-0.21) |
| Uganda | 39.02(25.71-59.70) | 0.75 (1.23 - 0.44) | 133.35(88.76-190.80) | 0.93 (1.53 - 0.54) | 0.25(0.11-0.39) |
| Ukraine | 40.19(33.36-47.77) | 0.20 (0.26 - 0.16) | 26.52(18.36-35.07) | 0.16 (0.22 - 0.10) | 0.01(-0.48-0.49) |
| United Arab Emirates | 4.23(2.66-6.72) | 0.40 (0.77 - 0.21) | 27.76(17.57-40.01) | 0.57 (0.91 - 0.31) | -1.82(-2.70--0.93) |
| United Kingdom | 27.85(27.35-28.44) | 0.13 (0.14 - 0.13) | 87.92(84.91-91.31) | 0.37 (0.39 - 0.36) | 1.17(0.84-1.50) |
| United Republic of Tanzania | 55.79(38.24-77.37) | 0.68 (1.12 - 0.40) | 112.48(71.93-171.53) | 0.55 (0.92 - 0.30) | 4.12(3.85-4.39) |
| United States of America | 188.77(185.14-192.51) | 0.17 (0.18 - 0.17) | 289.82(277.53-302.44) | 0.25 (0.26 - 0.24) | -1.10(-1.23--0.96) |
| United States Virgin Islands | 0.06(0.04-0.10) | 0.16 (0.28 - 0.09) | 0.04(0.03-0.06) | 0.16 (0.29 - 0.08) | 1.19(1.01-1.38) |
| Uruguay | 0.60(0.50-0.71) | 0.05 (0.07 - 0.04) | 1.46(1.24-1.73) | 0.12 (0.15 - 0.09) | -1.21(-1.80--0.63) |
| Uzbekistan | 29.60(21.75-38.45) | 0.38 (0.50 - 0.27) | 60.23(44.89-78.30) | 0.41 (0.55 - 0.31) | 3.37(2.89-3.85) |
| Vanuatu | 0.30(0.16-0.58) | 0.58 (1.24 - 0.27) | 0.66(0.38-1.11) | 0.59 (1.07 - 0.29) | 0.19(-0.03-0.41) |
| Venezuela (Bolivarian Republic of) | 19.22(18.25-20.25) | 0.26 (0.29 - 0.23) | 30.46(23.46-38.63) | 0.32 (0.40 - 0.24) | -0.08(-0.23-0.07) |
| Viet Nam | 380.82(269.92-530.60) | 1.54 (2.48 - 0.91) | 454.03(295.12-748.02) | 1.05 (1.93 - 0.56) | 0.63(-0.18-1.45) |
| Yemen | 8.63(2.87-19.58) | 0.22 (0.53 - 0.07) | 19.37(8.56-41.17) | 0.15 (0.34 - 0.06) | -1.38(-1.58--1.18) |
| Zambia | 29.80(19.45-47.22) | 1.22 (2.20 - 0.67) | 37.86(13.13-106.62) | 0.54 (1.60 - 0.17) | -1.32(-1.48--1.15) |
| Zimbabwe | 40.08(22.69-63.27) | 1.24 (2.15 - 0.65) | 119.31(70.82-184.47) | 2.09 (3.53 - 1.10) | -3.99(-4.75--3.22) |

**Table S4. Mortality cases of AYA liver cancer in 2021, and the age-standardized rates by sex and global、GBD region.**

| **regions** | **Mortality** | | | | | | | | | | |
| --- | --- | --- | --- | --- | --- | --- | --- | --- | --- | --- | --- |
|  | **both sexes** | | | **female** | | | | | **male** | | |
|  | **cases** | **ASR per 100,000** | | **cases** | | **ASR per 100,000** | | | **cases** | | **ASR per 100,000** |
| Global | 19270.22(17036.81-22393.10) | | 0.63 (0.74 to 0.55) | | 4810.98(4313.35-5380.01) | | 0.32 (0.37 to 0.28) | 14459.24(12391.99-17496.62) | | 0.93 (1.13 to 0.79) | |
| Eastern Europe | 170.15(157.29-182.78) | | 0.21 (0.23 to 0.20) | | 59.89(53.16-67.24) | | 0.16 (0.18 to 0.14) | 110.27(101.10-120.26) | | 0.27 (0.30 to 0.24) | |
| Western Europe | 306.86(292.48-320.86) | | 0.22 (0.23 to 0.20) | | 108.73(103.64-114.35) | | 0.16 (0.17 to 0.14) | 198.12(185.23-210.51) | | 0.28 (0.31 to 0.25) | |
| North Africa and Middle East | 907.53(769.55-1055.12) | | 0.34 (0.42 to 0.28) | | 416.42(345.96-500.51) | | 0.33 (0.42 to 0.26) | 491.11(411.48-587.50) | | 0.36 (0.46 to 0.28) | |
| Caribbean | 30.36(23.83-37.50) | | 0.16 (0.22 to 0.13) | | 12.08(9.18-16.82) | | 0.13 (0.19 to 0.09) | 18.29(14.77-22.37) | | 0.20 (0.27 to 0.15) | |
| East Asia | 8883.85(7029.84-11422.96) | | 1.53 (1.96 to 1.18) | | 1140.16(852.81-1501.47) | | 0.42 (0.57 to 0.31) | 7743.69(5980.50-10228.30) | | 2.56 (3.41 to 1.92) | |
| Central Latin America | 201.70(180.72-222.92) | | 0.20 (0.22 to 0.18) | | 86.03(74.34-97.95) | | 0.17 (0.19 to 0.14) | 115.66(104.56-126.36) | | 0.24 (0.26 to 0.21) | |
| Central Europe | 58.49(51.96-65.28) | | 0.15 (0.17 to 0.13) | | 17.41(15.06-20.05) | | 0.09 (0.11 to 0.08) | 41.07(36.44-46.39) | | 0.20 (0.23 to 0.17) | |
| Western Sub-Saharan Africa | 2014.27(1543.64-2534.55) | | 1.23 (1.59 to 0.92) | | 647.75(470.31-846.58) | | 0.73 (0.99 to 0.52) | 1366.52(1045.66-1757.58) | | 1.78 (2.37 to 1.31) | |
| Central Asia | 207.10(171.19-245.73) | | 0.52 (0.63 to 0.42) | | 92.87(77.22-110.54) | | 0.47 (0.58 to 0.38) | 114.23(91.74-138.38) | | 0.57 (0.71 to 0.45) | |
| Southeast Asia | 1905.02(1547.15-2545.24) | | 0.67 (0.92 to 0.52) | | 435.93(279.64-588.91) | | 0.31 (0.43 to 0.19) | 1469.09(1145.56-2041.97) | | 1.02 (1.47 to 0.75) | |
| High-income Asia Pacific | 205.76(169.91-258.74) | | 0.35 (0.45 to 0.27) | | 50.79(42.75-62.09) | | 0.18 (0.24 to 0.14) | 154.98(120.38-207.09) | | 0.50 (0.70 to 0.37) | |
| Southern Sub-Saharan Africa | 471.32(377.29-587.72) | | 1.35 (1.75 to 1.04) | | 162.21(124.09-205.00) | | 0.93 (1.26 to 0.66) | 309.11(235.12-427.26) | | 1.79 (2.53 to 1.29) | |
| Eastern Sub-Saharan Africa | 806.47(581.95-1141.00) | | 0.53 (0.76 to 0.37) | | 395.11(296.94-520.95) | | 0.48 (0.65 to 0.35) | 411.35(276.17-627.28) | | 0.58 (0.92 to 0.37) | |
| South Asia | 2265.34(1957.59-2712.57) | | 0.29 (0.36 to 0.25) | | 852.38(700.56-1057.60) | | 0.22 (0.28 to 0.18) | 1412.97(1175.02-1728.01) | | 0.36 (0.46 to 0.30) | |
| Tropical Latin America | 134.06(126.97-141.40) | | 0.14 (0.15 to 0.13) | | 51.83(48.76-54.48) | | 0.11 (0.12 to 0.10) | 82.23(76.56-88.59) | | 0.18 (0.20 to 0.16) | |
| Oceania | 22.09(13.82-40.01) | | 0.42 (0.79 to 0.24) | | 9.44(6.01-15.99) | | 0.35 (0.67 to 0.20) | 12.64(7.14-27.10) | | 0.48 (1.05 to 0.26) | |
| High-income North America | 322.40(309.51-336.12) | | 0.25 (0.26 to 0.24) | | 119.18(113.89-124.00) | | 0.19 (0.20 to 0.17) | 203.22(192.26-214.74) | | 0.31 (0.33 to 0.29) | |
| Central Sub-Saharan Africa | 226.73(97.83-560.60) | | 0.48 (1.16 to 0.20) | | 103.56(44.81-243.31) | | 0.43 (1.07 to 0.17) | 123.17(47.16-357.64) | | 0.53 (1.56 to 0.19) | |
| Australasia | 37.64(33.25-43.05) | | 0.33 (0.40 to 0.27) | | 13.24(11.36-15.30) | | 0.23 (0.30 to 0.18) | 24.40(20.33-29.41) | | 0.42 (0.56 to 0.32) | |
| Andean Latin America | 68.70(52.65-89.05) | | 0.25 (0.36 to 0.18) | | 27.07(18.99-37.03) | | 0.20 (0.29 to 0.13) | 41.62(31.33-54.68) | | 0.31 (0.46 to 0.21) | |
| Southern Latin America | 24.38(21.32-27.51) | | 0.09 (0.11 to 0.08) | | 8.89(7.75-10.46) | | 0.07 (0.08 to 0.05) | 15.50(12.83-18.60) | | 0.12 (0.15 to 0.09) | |
| **SDI regions** |  | |  | |  | |  |  | |  | |
| High SDI | 1122.97(1060.27-1191.16) | | 0.28 (0.31 to 0.26) | | 344.54(327.87-362.31) | | 0.18 (0.20 to 0.17) | 778.43(717.19-842.88) | | 0.38 (0.42 to 0.34) | |
| Low SDI | 2512.13(1963.58-3374.09) | | 0.64 (0.87 to 0.49) | | 1039.03(792.69-1371.50) | | 0.50 (0.67 to 0.38) | 1473.09(1139.14-2121.79) | | 0.78 (1.12 to 0.58) | |
| Middle SDI | 8184.62(6945.89-9954.80) | | 0.82 (1.01 to 0.68) | | 1513.36(1297.87-1763.76) | | 0.31 (0.37 to 0.26) | 6671.26(5520.01-8461.43) | | 1.32 (1.69 to 1.06) | |
| High-middle SDI | 3960.24(3172.53-4987.75) | | 0.75 (0.96 to 0.59) | | 611.03(490.82-769.36) | | 0.25 (0.32 to 0.20) | 3349.21(2599.86-4360.96) | | 1.23 (1.61 to 0.93) | |
| Low-middle SDI | 3482.89(2979.80-4135.12) | | 0.45 (0.54 to 0.38) | | 1300.49(1127.33-1505.63) | | 0.34 (0.40 to 0.28) | 2182.41(1813.44-2663.89) | | 0.57 (0.72 to 0.45) | |

**Table S5. DALYs of liver cancer among AYA between 1990 and 2021 at national level.**

| Nations | Number of cases, 1990 | Age-standardized rate per 100,000 population, 1990 | Number of cases, 2021 | Age-standardized rate per 100,000 population, 2021 | Estimated annual percentage change, 1990–2021 |
| --- | --- | --- | --- | --- | --- |
| Afghanistan | 1213.86(842.60-1712.96) | 50.55 (82.99 - 28.82) | 4207.68(2954.01-5983.75) | 40.69 (65.17 - 23.58) | -1.45(-1.77--1.14) |
| Albania | 715.85(523.25-933.91) | 52.27 (73.06 - 35.90) | 288.55(207.72-402.55) | 29.95 (47.01 - 18.82) | -2.44(-2.78--2.10) |
| Algeria | 799.97(599.12-1058.46) | 9.15 (13.66 - 5.92) | 1849.52(1284.10-2611.10) | 10.02 (15.47 - 6.22) | 0.10(-0.02-0.22) |
| American Samoa | 5.51(3.85-7.74) | 30.10 (49.18 - 18.02) | 9.48(6.18-13.34) | 56.54 (88.63 - 33.47) | 2.58(2.16-3.01) |
| Andorra | 9.70(6.60-14.06) | 36.43 (60.15 - 20.59) | 9.15(5.71-13.41) | 31.84 (52.96 - 16.89) | -0.49(-0.56--0.42) |
| Angola | 2605.54(379.13-8180.12) | 74.47 (251.43 - 10.04) | 5016.27(1179.82-15246.40) | 46.82 (142.76 - 9.94) | -1.49(-1.65--1.34) |
| Antigua and Barbuda | 3.34(2.96-3.76) | 13.69 (16.05 - 11.76) | 2.84(2.44-3.26) | 7.98 (9.40 - 6.62) | -1.51(-2.01--1.01) |
| Argentina | 287.77(240.14-346.17) | 2.40 (3.13 - 1.85) | 906.74(778.62-1044.05) | 5.06 (6.41 - 3.95) | 2.89(2.65-3.14) |
| Armenia | 374.41(311.99-461.74) | 26.05 (32.14 - 21.52) | 241.35(197.77-288.56) | 19.94 (23.81 - 16.33) | -1.28(-1.82--0.74) |
| Australia | 708.24(636.15-789.35) | 10.16 (12.52 - 8.26) | 1789.63(1563.39-2077.93) | 18.86 (23.89 - 14.89) | 1.80(1.54-2.06) |
| Austria | 303.76(272.91-343.85) | 10.08 (12.48 - 8.04) | 362.34(314.63-409.65) | 11.85 (14.97 - 9.18) | 0.01(-0.19-0.22) |
| Azerbaijan | 955.15(549.25-1526.55) | 32.17 (53.14 - 17.37) | 1353.54(760.83-2319.46) | 29.53 (52.07 - 15.64) | -1.43(-1.84--1.02) |
| Bahamas | 22.86(20.16-26.03) | 20.90 (24.44 - 18.00) | 31.82(24.96-39.74) | 20.45 (25.91 - 16.01) | -0.65(-1.01--0.29) |
| Bahrain | 45.20(35.19-61.91) | 16.87 (26.13 - 10.72) | 82.22(57.38-110.57) | 10.63 (17.14 - 6.28) | -2.09(-2.68--1.51) |
| Bangladesh | 5043.05(3589.31-7223.89) | 13.36 (22.95 - 7.71) | 8635.38(5276.09-14459.17) | 5.26 (10.21 - 2.64) | -0.57(-3.38-2.31) |
| Barbados | 12.60(11.38-14.31) | 11.54 (13.41 - 9.98) | 10.43(8.16-13.48) | 10.05 (13.13 - 7.71) | -1.03(-1.35--0.71) |
| Belarus | 455.44(382.18-548.63) | 10.86 (14.38 - 8.10) | 415.63(316.44-530.50) | 12.09 (16.51 - 8.67) | -0.33(-0.77-0.12) |
| Belgium | 321.21(284.42-360.10) | 8.27 (10.19 - 6.61) | 360.59(311.07-416.28) | 9.51 (12.24 - 7.35) | 0.30(0.08-0.53) |
| Belize | 5.78(5.04-6.78) | 8.94 (10.74 - 7.60) | 26.40(23.13-29.79) | 14.90 (17.25 - 12.63) | 0.79(0.26-1.32) |
| Benin | 2475.94(1259.12-3964.14) | 163.00 (301.26 - 72.77) | 5238.65(3357.75-7386.50) | 114.97 (194.69 - 61.49) | -1.53(-1.81--1.25) |
| Bermuda | 3.12(2.81-3.45) | 11.17 (12.92 - 9.67) | 1.06(0.85-1.31) | 5.32 (6.76 - 4.19) | -2.51(-3.00--2.01) |
| Bhutan | 47.37(26.32-75.22) | 21.12 (38.61 - 9.93) | 74.94(43.83-119.88) | 21.36 (38.31 - 11.03) | -0.17(-0.40-0.06) |
| Bolivia (Plurinational State of) | 440.10(296.37-609.91) | 19.18 (30.57 - 11.31) | 700.82(447.96-1129.03) | 14.47 (25.25 - 8.24) | -1.32(-1.58--1.06) |
| Bosnia and Herzegovina | 531.31(447.00-633.88) | 27.62 (35.62 - 20.74) | 140.08(98.20-182.40) | 12.94 (18.05 - 8.25) | -3.47(-4.06--2.88) |
| Botswana | 203.88(85.82-431.85) | 46.99 (106.83 - 17.57) | 580.32(247.01-1374.17) | 52.13 (134.66 - 19.48) | -0.78(-1.34--0.21) |
| Brazil | 5915.65(5686.71-6159.25) | 10.06 (10.81 - 9.37) | 7458.99(7079.87-7791.63) | 8.34 (8.94 - 7.74) | -0.03(-0.27-0.21) |
| Brunei Darussalam | 59.92(43.56-83.79) | 49.32 (76.06 - 30.20) | 68.89(50.60-94.26) | 30.54 (46.95 - 19.39) | -1.93(-2.13--1.74) |
| Bulgaria | 1000.09(767.27-1248.40) | 32.04 (40.61 - 24.06) | 283.46(206.56-390.71) | 13.10 (18.22 - 9.28) | -2.46(-2.82--2.09) |
| Burkina Faso | 5505.19(2364.79-12323.31) | 201.68 (483.42 - 77.15) | 12531.88(5445.73-24071.26) | 164.65 (334.58 - 65.50) | -0.69(-0.92--0.46) |
| Burundi | 648.40(401.33-1177.95) | 33.74 (66.15 - 17.01) | 992.86(609.74-1599.19) | 19.94 (37.16 - 10.11) | -1.95(-2.09--1.81) |
| Cabo Verde | 156.36(109.19-223.53) | 145.25 (243.99 - 82.70) | 295.47(206.50-410.02) | 113.97 (195.41 - 63.01) | -0.87(-1.21--0.52) |
| Cambodia | 1860.42(1000.16-3307.05) | 55.14 (107.50 - 26.32) | 2691.05(1279.14-5422.50) | 36.99 (78.53 - 16.08) | -1.63(-1.74--1.51) |
| Cameroon | 5278.70(3669.39-7908.37) | 160.38 (269.89 - 92.49) | 13750.14(7693.30-23884.26) | 119.05 (232.90 - 58.22) | -1.28(-1.44--1.12) |
| Canada | 1084.26(961.63-1225.52) | 9.09 (11.05 - 7.49) | 1895.17(1621.63-2183.55) | 14.80 (18.50 - 11.71) | 1.85(1.57-2.14) |
| Central African Republic | 613.46(227.20-1291.24) | 67.66 (156.79 - 23.83) | 810.73(318.20-1807.81) | 42.06 (98.02 - 15.28) | -2.24(-2.48--1.99) |
| Chad | 2374.32(920.25-5127.62) | 129.54 (292.27 - 45.75) | 5895.01(3166.63-11084.93) | 112.78 (220.73 - 53.68) | -0.68(-0.95--0.40) |
| Chile | 206.35(164.86-254.64) | 3.85 (5.12 - 2.84) | 430.09(370.06-513.13) | 5.76 (7.41 - 4.49) | 1.83(1.54-2.12) |
| China | 627737.35(522228.04-750609.77) | 123.48 (150.34 - 101.75) | 488460.79(384828.12-628201.35) | 87.71 (113.54 - 67.62) | -1.79(-2.19--1.39) |
| Colombia | 1869.87(1702.01-2043.90) | 14.17 (17.09 - 11.60) | 1840.05(1531.95-2199.75) | 9.12 (11.68 - 6.94) | -1.68(-2.26--1.09) |
| Comoros | 54.63(23.77-83.93) | 36.30 (63.57 - 15.41) | 96.71(65.14-148.31) | 32.55 (54.83 - 17.55) | -1.13(-1.77--0.47) |
| Congo | 601.59(258.00-1215.09) | 75.94 (166.05 - 28.71) | 921.20(386.22-1928.68) | 43.74 (100.29 - 16.01) | -2.26(-2.55--1.97) |
| Cook Islands | 4.71(3.25-6.69) | 66.61 (107.19 - 39.49) | 3.71(2.43-5.37) | 65.58 (108.53 - 36.55) | 0.35(0.18-0.52) |
| Costa Rica | 245.85(215.66-276.59) | 20.50 (25.12 - 16.52) | 393.70(327.59-473.74) | 19.87 (25.40 - 15.36) | -0.54(-1.15-0.08) |
| Croatia | 250.49(193.41-315.51) | 12.96 (16.70 - 9.79) | 77.89(58.65-102.80) | 5.66 (7.52 - 4.16) | -3.07(-3.62--2.51) |
| Cuba | 514.34(442.97-599.97) | 11.45 (14.81 - 8.97) | 223.20(178.28-274.66) | 5.82 (7.54 - 4.30) | -2.73(-3.26--2.19) |
| Cyprus | 26.19(18.56-37.95) | 8.42 (14.10 - 5.07) | 36.26(25.66-49.63) | 6.09 (9.69 - 3.76) | -1.91(-2.41--1.41) |
| Czechia | 695.69(601.64-809.13) | 17.54 (21.64 - 14.08) | 186.08(141.59-234.26) | 5.61 (7.62 - 4.04) | -3.89(-4.31--3.46) |
| C么te d'Ivoire | 1925.33(1157.16-3014.68) | 45.94 (83.13 - 23.21) | 2960.04(1728.98-4553.75) | 27.94 (48.55 - 14.36) | -1.96(-2.17--1.75) |
| Democratic People's Republic of Korea | 9025.23(4410.46-15830.54) | 116.63 (227.39 - 51.53) | 8386.84(4474.99-15400.65) | 77.68 (151.84 - 38.17) | -1.75(-1.91--1.59) |
| Democratic Republic of the Congo | 3250.85(1627.90-6990.51) | 25.75 (56.00 - 11.20) | 6119.44(2868.39-14018.42) | 19.06 (46.09 - 8.02) | -1.21(-1.39--1.03) |
| Denmark | 113.39(100.20-127.21) | 5.75 (7.09 - 4.62) | 67.59(58.50-78.41) | 3.63 (4.67 - 2.78) | -2.03(-2.51--1.55) |
| Djibouti | 35.51(18.97-62.76) | 23.84 (44.37 - 11.80) | 137.43(73.25-236.36) | 25.20 (45.99 - 12.26) | 0.41(-0.00-0.83) |
| Dominica | 2.57(1.68-4.29) | 9.95 (19.36 - 5.28) | 3.25(2.26-4.65) | 12.50 (20.72 - 7.01) | 0.00(-0.54-0.55) |
| Dominican Republic | 259.54(191.46-344.19) | 9.56 (14.31 - 6.00) | 594.90(403.06-821.40) | 13.17 (20.74 - 7.84) | 1.11(0.86-1.35) |
| Ecuador | 1239.68(1083.63-1397.11) | 32.31 (40.58 - 25.52) | 1228.06(961.20-1558.02) | 16.95 (22.94 - 12.22) | -2.83(-3.66--1.99) |
| Egypt | 10324.50(7371.34-14750.83) | 51.79 (83.28 - 31.90) | 21081.93(16064.63-27389.45) | 50.68 (72.17 - 33.96) | 0.18(0.05-0.31) |
| El Salvador | 164.38(142.35-192.44) | 8.79 (11.41 - 6.77) | 201.08(158.81-252.07) | 8.18 (10.98 - 5.78) | -0.01(-0.40-0.37) |
| Equatorial Guinea | 29.76(14.56-62.51) | 22.30 (50.49 - 9.66) | 198.14(94.92-358.54) | 32.34 (63.32 - 13.85) | 1.25(0.92-1.58) |
| Eritrea | 279.32(169.55-467.00) | 24.97 (47.08 - 13.34) | 646.31(366.46-1168.19) | 24.62 (47.69 - 12.09) | -0.69(-0.98--0.40) |
| Estonia | 105.04(87.98-123.25) | 17.34 (22.57 - 13.27) | 52.96(44.30-62.72) | 12.24 (15.85 - 9.29) | -1.95(-2.25--1.64) |
| Eswatini | 174.61(67.75-342.69) | 71.03 (149.42 - 24.43) | 1014.84(300.33-2572.25) | 215.46 (612.56 - 58.09) | 3.83(2.23-5.45) |
| Ethiopia | 4047.50(2595.29-6035.73) | 24.28 (36.66 - 14.97) | 6498.51(4344.38-9660.50) | 15.48 (24.32 - 10.07) | -1.99(-2.21--1.77) |
| Fiji | 92.55(63.44-134.46) | 30.69 (51.05 - 17.68) | 119.60(76.76-174.35) | 33.09 (54.89 - 18.45) | 0.65(0.21-1.11) |
| Finland | 268.39(228.43-317.22) | 13.45 (16.95 - 10.71) | 172.21(145.14-205.58) | 9.63 (12.11 - 7.53) | -1.00(-1.28--0.72) |
| France | 3292.24(2976.14-3598.99) | 14.41 (17.40 - 11.90) | 3202.73(2804.16-3668.55) | 15.42 (19.67 - 11.81) | 0.26(0.05-0.46) |
| Gabon | 203.61(80.63-460.62) | 59.90 (148.84 - 21.27) | 362.30(186.18-641.57) | 52.92 (105.81 - 22.84) | -0.69(-0.95--0.44) |
| Gambia | 726.21(480.31-1030.33) | 225.81 (371.86 - 128.93) | 2460.87(1521.30-4002.62) | 280.72 (518.74 - 143.83) | 0.25(-0.05-0.57) |
| Georgia | 488.46(410.44-570.82) | 22.67 (26.66 - 19.02) | 150.04(128.11-174.01) | 11.90 (13.94 - 10.06) | -2.67(-3.24--2.11) |
| Germany | 2326.53(2028.84-2657.93) | 7.54 (9.38 - 5.95) | 2848.74(2465.88-3246.46) | 10.18 (12.76 - 8.01) | 0.88(0.75-1.01) |
| Ghana | 8268.21(4896.90-13272.96) | 159.38 (283.68 - 83.11) | 14473.65(9033.25-23764.63) | 46.37 (83.28 - 24.40) | -2.02(-2.61--1.42) |
| Greece | 481.43(448.61-514.57) | 12.69 (14.13 - 11.43) | 607.55(557.13-664.26) | 19.72 (22.10 - 17.58) | -1.49(-2.02--0.95) |
| Greenland | 9.08(6.57-12.50) | 33.36 (51.58 - 20.23) | 4.17(2.58-6.14) | 19.15 (30.88 - 10.79) | 1.19(0.99-1.38) |
| Grenada | 4.20(3.30-5.39) | 13.87 (19.03 - 9.97) | 6.11(5.02-7.54) | 15.82 (21.08 - 11.77) | -1.31(-1.75--0.86) |
| Guam | 11.97(9.97-14.90) | 19.60 (26.89 - 14.35) | 29.69(22.90-36.85) | 54.67 (74.98 - 37.48) | 0.00(-0.42-0.42) |
| Guatemala | 934.48(881.03-989.42) | 35.46 (38.96 - 32.06) | 1644.53(1415.02-1867.66) | 26.15 (30.39 - 21.97) | 3.90(3.68-4.13) |
| Guinea | 3721.06(2605.79-5166.64) | 196.51 (306.60 - 115.63) | 7557.72(4814.93-11247.08) | 164.53 (279.80 - 90.66) | -1.94(-2.92--0.96) |
| Guinea-Bissau | 955.04(402.93-1533.57) | 296.83 (536.45 - 110.80) | 1506.01(887.89-2260.38) | 197.11 (346.75 - 99.40) | -0.42(-0.61--0.23) |
| Guyana | 35.97(29.52-44.33) | 11.76 (14.38 - 9.56) | 44.31(32.95-56.47) | 15.28 (20.00 - 11.14) | -1.59(-1.73--1.45) |
| Haiti | 209.98(122.20-374.48) | 9.28 (17.48 - 4.90) | 375.34(185.93-722.16) | 6.96 (13.93 - 3.19) | 0.43(-0.09-0.94) |
| Honduras | 159.76(116.60-221.85) | 10.32 (15.85 - 6.20) | 322.82(181.19-500.57) | 7.95 (14.27 - 3.96) | -0.84(-0.97--0.72) |
| Hungary | 655.62(519.24-841.77) | 16.14 (21.85 - 11.97) | 155.70(117.82-199.70) | 5.18 (7.01 - 3.73) | -1.38(-1.79--0.96) |
| Iceland | 7.74(6.85-8.80) | 7.38 (9.05 - 5.89) | 10.98(9.21-13.23) | 8.73 (11.22 - 6.66) | -4.20(-4.92--3.48) |
| India | 40797.26(36243.07-47673.93) | 12.81 (15.56 - 11.01) | 95164.24(80343.91-116056.50) | 15.87 (19.88 - 13.12) | 0.12(-0.16-0.39) |
| Indonesia | 16863.28(10844.85-24607.68) | 23.90 (36.05 - 15.33) | 27956.12(17703.83-44446.34) | 23.88 (38.06 - 14.73) | 0.53(0.29-0.78) |
| Iran (Islamic Republic of) | 2550.10(2168.93-3141.95) | 12.89 (16.37 - 10.55) | 6106.96(5359.99-6872.48) | 15.57 (18.18 - 13.26) | 0.02(-0.07-0.11) |
| Iraq | 1315.33(947.67-1815.76) | 21.20 (33.21 - 12.94) | 2941.30(2046.42-4455.21) | 17.79 (28.45 - 10.95) | 0.26(-0.40-0.92) |
| Ireland | 75.95(68.28-84.44) | 5.66 (6.94 - 4.59) | 107.47(92.79-126.01) | 6.38 (8.01 - 4.97) | -0.31(-0.55--0.06) |
| Israel | 91.99(80.85-104.43) | 4.88 (6.16 - 3.82) | 195.49(168.46-225.19) | 5.85 (7.55 - 4.52) | -0.04(-0.60-0.52) |
| Italy | 3053.91(2921.37-3189.18) | 14.34 (15.38 - 13.37) | 1510.79(1419.60-1605.44) | 8.84 (9.68 - 8.09) | 0.36(-0.09-0.82) |
| Jamaica | 32.48(26.46-40.68) | 3.74 (4.96 - 2.82) | 82.10(58.70-113.07) | 6.87 (9.74 - 4.73) | -2.04(-2.41--1.66) |
| Japan | 10842.48(10576.95-11101.28) | 22.98 (23.79 - 22.16) | 3418.20(3320.12-3529.16) | 9.42 (9.78 - 9.06) | 1.01(0.23-1.81) |
| Jordan | 100.59(66.99-157.20) | 8.03 (13.72 - 4.58) | 254.96(177.50-352.42) | 4.97 (7.88 - 3.03) | -3.51(-4.01--3.01) |
| Kazakhstan | 3540.64(3172.81-3946.59) | 52.48 (59.12 - 46.74) | 1588.84(1312.35-1940.95) | 20.95 (25.56 - 17.15) | -2.08(-2.36--1.80) |
| Kenya | 1232.46(886.14-1813.33) | 16.43 (25.45 - 11.32) | 4763.28(3390.16-6981.08) | 24.43 (35.99 - 16.86) | -3.96(-4.53--3.38) |
| Kiribati | 19.29(14.20-26.79) | 69.21 (107.07 - 42.50) | 32.28(20.90-48.78) | 66.88 (110.11 - 37.61) | 1.32(1.03-1.62) |
| Kuwait | 141.81(121.31-167.43) | 15.97 (20.75 - 12.20) | 72.96(59.69-91.23) | 2.91 (3.85 - 2.17) | -0.42(-0.70--0.14) |
| Kyrgyzstan | 657.72(492.49-865.97) | 39.45 (53.58 - 28.98) | 259.39(176.50-356.84) | 9.47 (13.00 - 6.40) | -5.04(-6.05--4.01) |
| Lao People's Democratic Republic | 938.43(602.14-1319.69) | 69.57 (115.73 - 37.73) | 1401.08(894.89-2124.31) | 44.79 (78.22 - 24.11) | -4.02(-4.68--3.35) |
| Latvia | 146.16(121.15-172.87) | 14.65 (19.08 - 10.85) | 77.62(61.16-97.13) | 12.98 (17.77 - 9.49) | -1.81(-1.96--1.65) |
| Lebanon | 156.69(112.12-213.94) | 14.65 (22.56 - 9.15) | 212.49(162.03-280.92) | 8.52 (13.01 - 5.39) | -1.34(-1.71--0.97) |
| Lesotho | 209.33(67.23-538.50) | 43.29 (117.20 - 11.76) | 1186.80(394.29-3493.83) | 159.33 (511.69 - 44.90) | -1.86(-2.11--1.61) |
| Liberia | 1291.98(621.89-2067.93) | 154.35 (284.22 - 67.35) | 3203.52(1814.83-4912.37) | 156.40 (279.76 - 80.09) | 4.49(3.43-5.55) |
| Libya | 447.30(308.08-613.49) | 31.25 (50.79 - 18.16) | 1261.35(855.69-1829.58) | 39.32 (63.65 - 22.43) | -0.20(-0.51-0.11) |
| Lithuania | 170.71(147.69-194.99) | 12.11 (15.09 - 9.67) | 114.14(92.00-145.01) | 13.23 (17.49 - 9.96) | 1.00(0.76-1.24) |
| Luxembourg | 16.58(15.54-17.59) | 10.56 (11.70 - 9.52) | 19.72(17.95-21.86) | 8.00 (9.09 - 6.99) | -0.01(-0.35-0.33) |
| Madagascar | 1241.57(863.07-1772.67) | 30.52 (48.42 - 17.63) | 2318.67(1518.09-3628.93) | 21.75 (38.20 - 11.92) | -1.42(-1.72--1.12) |
| Malawi | 829.42(558.01-1228.85) | 25.21 (42.81 - 14.01) | 2363.17(1543.48-3488.00) | 33.27 (57.56 - 18.20) | -1.11(-1.29--0.93) |
| Malaysia | 1534.27(1184.96-2065.26) | 21.92 (34.79 - 13.00) | 3782.90(2812.36-5170.85) | 26.40 (43.79 - 15.64) | 0.34(-0.26-0.94) |
| Maldives | 18.03(10.03-28.28) | 29.04 (53.37 - 12.92) | 52.76(32.20-81.32) | 15.92 (28.19 - 8.38) | 0.28(0.02-0.55) |
| Mali | 4624.45(3559.01-5876.04) | 174.18 (256.47 - 114.22) | 13762.83(9645.53-18580.43) | 184.99 (302.07 - 106.84) | -2.71(-3.35--2.08) |
| Malta | 6.71(5.83-7.65) | 4.62 (5.76 - 3.68) | 11.23(9.54-13.65) | 7.57 (9.83 - 5.81) | 0.29(0.18-0.40) |
| Marshall Islands | 4.04(2.55-6.27) | 26.04 (48.51 - 13.36) | 8.36(4.98-14.37) | 35.86 (66.56 - 18.66) | 1.31(0.99-1.63) |
| Mauritania | 2105.42(435.48-4435.07) | 309.50 (732.46 - 57.58) | 2262.92(1100.87-3770.73) | 151.41 (290.61 - 64.28) | 0.76(0.51-1.02) |
| Mauritius | 86.34(79.88-94.06) | 17.74 (19.94 - 15.96) | 14.02(12.64-15.36) | 2.97 (3.36 - 2.61) | -2.64(-2.77--2.52) |
| Mexico | 2563.80(2513.48-2618.51) | 8.07 (8.35 - 7.80) | 5218.11(4698.65-5752.40) | 10.11 (11.19 - 9.04) | 0.38(-1.56-2.36) |
| Micronesia (Federated States of) | 15.89(10.39-23.73) | 43.80 (74.33 - 24.29) | 17.09(9.83-30.10) | 44.19 (83.36 - 22.21) | 1.16(1.00-1.32) |
| Monaco | 1.45(1.00-2.11) | 14.58 (23.27 - 8.86) | 3.01(1.85-4.71) | 30.41 (51.61 - 16.27) | -0.12(-0.36-0.13) |
| Mongolia | 2217.37(1459.79-3244.63) | 286.14 (457.86 - 164.01) | 3369.11(2351.13-4778.60) | 247.34 (374.07 - 160.45) | 2.21(1.61-2.82) |
| Montenegro | 66.67(53.08-82.85) | 26.31 (33.93 - 20.08) | 44.54(33.36-59.63) | 20.13 (27.67 - 14.85) | -0.76(-1.10--0.42) |
| Morocco | 214.59(153.32-293.52) | 2.28 (3.65 - 1.35) | 374.45(251.55-590.39) | 2.51 (4.63 - 1.40) | -0.88(-1.11--0.66) |
| Mozambique | 2077.84(1139.85-3279.63) | 49.31 (87.69 - 23.72) | 7278.39(3371.98-15064.94) | 74.15 (169.61 - 29.72) | 0.16(0.09-0.24) |
| Myanmar | 4000.96(1703.69-7678.00) | 25.85 (54.91 - 9.78) | 4583.96(2256.89-9071.59) | 20.98 (45.80 - 9.32) | 1.48(1.10-1.87) |
| Namibia | 62.39(34.55-111.33) | 12.90 (25.01 - 6.04) | 194.17(116.26-314.17) | 19.80 (36.86 - 9.85) | -0.87(-0.94--0.79) |
| Nauru | 2.58(1.70-3.67) | 68.38 (114.56 - 38.09) | 2.72(1.43-4.43) | 62.90 (113.17 - 29.02) | 0.89(0.06-1.72) |
| Nepal | 741.77(498.81-1070.19) | 11.10 (18.81 - 6.08) | 2105.26(1377.27-3038.39) | 16.90 (28.34 - 9.24) | -0.57(-0.72--0.42) |
| Netherlands | 308.87(269.84-361.58) | 4.96 (6.14 - 4.01) | 349.06(290.33-413.91) | 6.35 (7.97 - 5.03) | 1.62(1.41-1.82) |
| New Zealand | 176.03(150.55-206.10) | 12.72 (15.58 - 10.31) | 387.89(328.55-461.79) | 20.78 (26.18 - 16.62) | 0.58(0.31-0.86) |
| Nicaragua | 175.04(143.69-217.30) | 13.49 (19.26 - 9.28) | 351.34(265.64-453.74) | 12.59 (19.01 - 8.00) | 1.40(1.14-1.66) |
| Niger | 3465.38(1530.16-6854.64) | 143.60 (320.69 - 55.68) | 5296.21(3142.56-9439.05) | 73.62 (141.96 - 37.12) | -0.04(-0.44-0.36) |
| Nigeria | 9318.61(3905.89-18192.64) | 31.23 (62.49 - 13.15) | 18747.44(10548.22-31056.32) | 24.33 (39.98 - 13.72) | -2.34(-2.47--2.21) |
| Niue | 0.24(0.14-0.40) | 32.41 (58.15 - 16.96) | 0.19(0.12-0.31) | 33.58 (60.78 - 17.75) | -1.20(-1.41--0.98) |
| North Macedonia | 288.49(244.82-349.51) | 36.28 (47.89 - 27.72) | 166.88(126.26-210.76) | 19.59 (27.48 - 13.68) | -0.18(-0.43-0.07) |
| Northern Mariana Islands | 8.03(5.01-12.17) | 33.32 (56.55 - 18.43) | 3.32(2.38-4.49) | 20.12 (32.43 - 12.07) | -2.48(-2.82--2.13) |
| Norway | 127.02(120.72-134.25) | 7.73 (8.40 - 7.13) | 304.48(280.78-333.28) | 16.27 (18.16 - 14.72) | -1.56(-2.02--1.10) |
| Oman | 90.87(55.46-153.81) | 10.79 (20.25 - 5.57) | 382.90(255.85-546.61) | 14.07 (23.28 - 8.02) | 2.11(1.58-2.65) |
| Pakistan | 7551.53(5689.62-9972.28) | 21.01 (30.11 - 14.28) | 26512.84(19775.85-35752.61) | 28.17 (40.06 - 19.17) | 1.05(0.75-1.34) |
| Palau | 3.88(2.10-6.91) | 58.95 (120.28 - 24.91) | 5.73(3.64-8.93) | 87.01 (159.16 - 43.57) | 0.74(0.42-1.06) |
| Palestine | 157.20(107.77-227.57) | 24.31 (39.11 - 14.56) | 339.51(265.30-435.47) | 16.97 (24.82 - 11.07) | 1.31(1.03-1.59) |
| Panama | 111.94(104.69-117.91) | 11.86 (13.12 - 10.63) | 204.64(170.16-243.51) | 12.52 (15.24 - 10.04) | -1.41(-1.74--1.07) |
| Papua New Guinea | 403.27(157.19-984.74) | 27.30 (71.62 - 9.33) | 788.65(377.14-1747.65) | 19.38 (46.69 - 7.99) | 0.57(0.09-1.05) |
| Paraguay | 135.87(108.03-166.70) | 9.30 (13.11 - 6.40) | 409.08(281.93-569.35) | 13.49 (20.93 - 7.99) | -1.42(-1.59--1.26) |
| Peru | 1291.56(925.56-1832.12) | 15.68 (24.96 - 9.48) | 2201.83(1470.54-3173.25) | 14.73 (23.79 - 8.37) | 1.63(1.42-1.85) |
| Philippines | 19526.46(14242.18-22888.06) | 86.47 (103.94 - 62.25) | 21429.94(17742.80-25689.31) | 47.87 (58.31 - 39.27) | -0.32(-0.54--0.10) |
| Poland | 424.09(387.78-473.47) | 2.71 (3.07 - 2.45) | 814.17(739.73-899.46) | 5.73 (6.35 - 5.15) | -2.17(-2.30--2.04) |
| Portugal | 422.50(364.40-487.51) | 11.27 (14.19 - 8.95) | 297.12(251.97-363.16) | 9.27 (11.99 - 7.14) | 3.39(2.46-4.33) |
| Puerto Rico | 231.39(193.17-279.22) | 16.86 (22.60 - 12.51) | 184.89(148.61-225.70) | 17.34 (23.44 - 12.41) | -1.37(-1.75--0.99) |
| Qatar | 62.92(44.03-96.97) | 24.09 (40.86 - 14.22) | 483.82(339.86-659.93) | 26.35 (43.04 - 15.38) | -1.14(-1.96--0.32) |
| Republic of Korea | 28625.58(19500.06-40024.35) | 144.34 (228.34 - 85.07) | 7824.59(5908.06-10700.96) | 41.51 (60.58 - 28.28) | -0.56(-0.97--0.14) |
| Republic of Moldova | 284.47(266.60-306.26) | 15.76 (17.42 - 14.29) | 208.57(181.52-235.15) | 14.77 (17.11 - 12.66) | -4.67(-4.89--4.45) |
| Romania | 750.72(597.29-913.29) | 8.50 (11.33 - 6.19) | 481.94(383.31-606.01) | 8.34 (11.18 - 5.94) | -0.33(-0.68-0.02) |
| Russian Federation | 6226.34(6029.99-6413.40) | 10.24 (10.65 - 9.83) | 7368.44(6866.24-7847.60) | 13.54 (14.59 - 12.54) | -0.24(-0.56-0.08) |
| Rwanda | 1255.93(814.58-1785.84) | 50.03 (81.41 - 27.08) | 1490.22(926.47-2360.45) | 28.02 (50.08 - 14.95) | 1.33(0.66-2.01) |
| Saint Kitts and Nevis | 3.34(2.96-3.84) | 21.42 (25.25 - 18.16) | 1.99(1.39-2.77) | 8.34 (11.64 - 5.70) | -2.68(-3.18--2.19) |
| Saint Lucia | 5.82(5.01-6.81) | 12.15 (14.61 - 10.13) | 7.09(5.87-8.47) | 10.17 (12.29 - 8.21) | -2.62(-3.69--1.54) |
| Saint Vincent and the Grenadines | 7.49(6.59-8.65) | 19.13 (22.62 - 16.22) | 6.88(5.88-7.90) | 16.51 (19.38 - 13.71) | -1.16(-1.52--0.81) |
| Samoa | 14.33(9.14-21.56) | 25.75 (44.79 - 13.92) | 19.14(10.43-30.96) | 26.06 (47.04 - 12.92) | -1.02(-1.33--0.70) |
| San Marino | 0.50(0.38-0.65) | 5.35 (8.37 - 3.27) | 0.73(0.44-1.13) | 7.92 (13.41 - 4.19) | -0.26(-0.42--0.09) |
| Sao Tome and Principe | 11.21(7.05-16.29) | 33.17 (58.50 - 17.33) | 22.03(11.42-43.94) | 26.01 (55.77 - 11.24) | 1.58(1.45-1.70) |
| Saudi Arabia | 1415.90(888.62-2224.02) | 23.40 (40.36 - 12.79) | 3110.56(2000.65-4477.63) | 14.90 (23.83 - 8.51) | -1.06(-1.52--0.59) |
| Senegal | 2873.53(1613.61-4336.15) | 121.21 (210.42 - 61.82) | 4502.24(2830.07-6656.45) | 78.78 (130.44 - 43.48) | -2.14(-2.51--1.78) |
| Serbia | 739.70(541.42-992.64) | 19.89 (30.62 - 12.37) | 400.06(276.08-576.60) | 12.27 (19.80 - 7.22) | -1.41(-1.57--1.24) |
| Seychelles | 12.97(9.36-17.31) | 48.40 (76.59 - 28.68) | 7.76(5.45-11.04) | 18.32 (30.52 - 9.68) | -1.83(-1.95--1.72) |
| Sierra Leone | 1935.87(609.72-3734.73) | 133.96 (290.89 - 38.42) | 2844.07(1795.13-4424.81) | 86.07 (154.89 - 44.92) | -2.74(-3.20--2.27) |
| Singapore | 515.73(444.95-596.84) | 33.56 (42.07 - 26.65) | 238.90(196.37-289.90) | 9.66 (12.69 - 7.26) | -1.39(-1.64--1.14) |
| Slovakia | 474.59(337.85-659.44) | 21.69 (30.90 - 14.99) | 237.12(148.45-360.80) | 12.03 (18.68 - 7.43) | -4.80(-5.30--4.29) |
| Slovenia | 171.80(152.55-195.75) | 20.91 (25.94 - 16.81) | 50.72(41.44-61.61) | 7.76 (10.39 - 5.78) | -2.17(-2.49--1.85) |
| Solomon Islands | 49.28(14.76-107.06) | 47.59 (110.08 - 12.98) | 109.21(66.71-169.69) | 43.77 (78.11 - 22.60) | -3.78(-4.14--3.42) |
| Somalia | 1277.82(550.89-2322.64) | 48.08 (100.38 - 19.51) | 3330.25(1509.00-6406.80) | 46.35 (96.37 - 18.18) | -0.35(-0.62--0.08) |
| South Africa | 10558.95(6870.52-15880.54) | 76.14 (113.03 - 48.28) | 17125.41(14136.63-20552.98) | 65.98 (82.34 - 53.08) | -0.39(-0.65--0.13) |
| South Sudan | 616.90(374.05-979.08) | 31.35 (57.15 - 16.00) | 1238.27(781.75-1924.16) | 38.84 (68.04 - 20.21) | -1.48(-2.70--0.25) |
| Spain | 2411.51(2166.06-2672.47) | 16.61 (20.27 - 13.59) | 1635.67(1402.48-1917.32) | 11.93 (15.31 - 9.29) | 0.62(0.33-0.92) |
| Sri Lanka | 796.50(597.92-1048.32) | 11.26 (17.87 - 6.84) | 593.24(368.47-883.46) | 7.21 (12.32 - 3.86) | -1.43(-1.62--1.24) |
| Sudan | 1347.64(749.49-2402.78) | 19.94 (37.83 - 9.96) | 3277.29(1983.51-5198.48) | 19.16 (33.36 - 9.84) | -2.44(-3.00--1.88) |
| Suriname | 18.67(11.69-26.21) | 13.06 (21.26 - 7.32) | 31.87(21.40-46.45) | 14.70 (24.68 - 8.38) | 0.04(-0.13-0.20) |
| Sweden | 352.07(322.00-385.07) | 11.63 (13.63 - 9.86) | 390.07(339.77-457.19) | 11.33 (13.82 - 9.16) | 0.03(-0.27-0.33) |
| Switzerland | 440.50(375.63-519.08) | 15.93 (20.26 - 12.44) | 193.66(163.86-231.40) | 6.28 (8.03 - 4.79) | 1.04(-0.02-2.11) |
| Syrian Arab Republic | 1529.12(1178.76-2024.47) | 37.69 (52.16 - 26.50) | 945.59(665.05-1321.24) | 20.20 (29.81 - 13.21) | -3.30(-3.51--3.09) |
| Taiwan (Province of China) | 8604.42(7761.57-9412.55) | 91.18 (107.52 - 76.47) | 4678.40(3905.30-5622.86) | 52.57 (67.04 - 40.63) | -2.48(-3.12--1.84) |
| Tajikistan | 717.00(429.80-1123.64) | 38.23 (62.21 - 21.55) | 979.48(591.69-1503.70) | 23.69 (38.10 - 13.63) | -1.36(-2.22--0.50) |
| Thailand | 18698.33(13019.52-26875.16) | 77.72 (122.76 - 47.89) | 20427.93(13556.93-29018.37) | 87.24 (141.34 - 51.01) | -2.48(-2.86--2.11) |
| Timor-Leste | 57.46(32.25-96.41) | 19.82 (35.02 - 10.00) | 72.25(38.71-133.86) | 15.21 (28.77 - 7.51) | -0.56(-1.01--0.10) |
| Togo | 930.20(635.90-1418.74) | 79.14 (135.49 - 47.08) | 2071.27(1278.16-3567.37) | 66.92 (123.19 - 36.87) | -0.99(-1.52--0.47) |
| Tokelau | 0.16(0.09-0.30) | 30.01 (59.90 - 14.71) | 0.17(0.12-0.27) | 36.67 (65.66 - 19.88) | -0.83(-1.04--0.62) |
| Tonga | 37.42(24.73-57.29) | 130.77 (218.25 - 75.04) | 49.63(29.89-81.20) | 143.57 (263.32 - 76.81) | 0.59(0.35-0.82) |
| Trinidad and Tobago | 55.08(49.63-61.62) | 11.43 (13.10 - 10.01) | 75.27(57.00-95.56) | 13.35 (17.14 - 10.05) | 0.04(-0.35-0.43) |
| Tunisia | 244.65(177.11-331.59) | 7.97 (12.81 - 4.84) | 447.97(292.87-642.50) | 9.34 (15.34 - 5.22) | 0.06(-0.25-0.38) |
| Turkey | 2613.34(1992.83-3453.59) | 11.80 (18.08 - 7.39) | 3089.45(2347.44-4050.78) | 9.34 (14.03 - 5.93) | 0.39(0.32-0.45) |
| Turkmenistan | 445.83(400.30-493.45) | 31.18 (35.07 - 27.59) | 583.42(435.49-766.32) | 28.04 (36.97 - 20.98) | -1.12(-1.69--0.55) |
| Tuvalu | 1.25(0.86-1.92) | 35.76 (59.98 - 20.67) | 1.80(1.13-2.76) | 38.62 (69.29 - 21.11) | -0.08(-0.31-0.15) |
| Uganda | 2342.72(1543.98-3613.84) | 43.71 (71.64 - 25.69) | 8048.05(5281.31-11457.86) | 54.63 (89.62 - 31.63) | 0.23(0.09-0.37) |
| Ukraine | 2396.45(1998.60-2843.56) | 12.23 (15.90 - 9.37) | 1510.78(1046.82-1986.48) | 9.16 (12.61 - 6.14) | 0.02(-0.47-0.51) |
| United Arab Emirates | 247.63(157.03-393.85) | 23.89 (46.20 - 12.84) | 1564.17(1006.75-2243.72) | 34.70 (55.44 - 19.34) | -1.93(-2.81--1.05) |
| United Kingdom | 1649.22(1619.42-1683.09) | 7.80 (8.06 - 7.58) | 5114.76(4946.04-5303.74) | 21.83 (22.78 - 20.96) | 1.28(0.93-1.64) |
| United Republic of Tanzania | 3349.68(2327.01-4648.53) | 40.00 (65.15 - 23.57) | 6737.27(4381.57-10319.38) | 32.19 (54.19 - 17.80) | 4.09(3.82-4.35) |
| United States of America | 10951.33(10760.87-11161.60) | 10.12 (10.40 - 9.87) | 17016.49(16305.60-17747.73) | 14.68 (15.41 - 13.96) | -1.08(-1.22--0.95) |
| United States Virgin Islands | 3.78(2.51-5.58) | 9.56 (16.35 - 5.25) | 2.33(1.47-3.57) | 9.34 (17.00 - 4.85) | 1.23(1.05-1.40) |
| Uruguay | 34.53(29.26-40.97) | 3.09 (4.07 - 2.30) | 84.81(72.45-99.44) | 6.92 (9.00 - 5.22) | -1.28(-1.89--0.67) |
| Uzbekistan | 1762.26(1296.60-2292.81) | 22.27 (29.40 - 16.12) | 3510.18(2610.70-4572.98) | 24.32 (32.10 - 18.02) | 3.40(2.93-3.88) |
| Vanuatu | 17.60(9.55-33.54) | 33.61 (71.35 - 15.74) | 38.67(22.59-63.93) | 33.95 (61.77 - 16.80) | 0.17(-0.05-0.39) |
| Venezuela (Bolivarian Republic of) | 1140.62(1084.75-1197.17) | 15.24 (16.82 - 13.75) | 1823.02(1409.79-2312.27) | 19.09 (24.45 - 14.48) | -0.09(-0.24-0.06) |
| Viet Nam | 22140.16(15725.73-30518.07) | 88.26 (142.22 - 52.41) | 25909.80(17055.93-42178.86) | 60.77 (110.73 - 32.31) | 0.74(-0.05-1.54) |
| Yemen | 502.71(164.25-1153.21) | 12.50 (30.77 - 3.84) | 1131.55(500.74-2416.18) | 8.83 (20.08 - 3.50) | -1.35(-1.55--1.15) |
| Zambia | 1807.91(1175.46-2888.38) | 71.64 (129.90 - 39.24) | 2252.27(783.09-6387.21) | 31.33 (93.48 - 9.70) | -1.28(-1.46--1.10) |
| Zimbabwe | 2378.51(1353.04-3725.17) | 72.19 (124.42 - 37.89) | 7040.64(4179.24-10874.20) | 122.12 (205.34 - 64.33) | -4.00(-4.75--3.24) |

**Table S6. DALYs cases of AYA liver cancer in 2021, and the age-standardized rates by sex and global、GBD region.**

| **regions** | **DALYs** | | | | | |
| --- | --- | --- | --- | --- | --- | --- |
|  | **both sexes** | | **female** | | **male** | |
|  | **cases** | **ASR per 100,000** | **cases** | **ASR per 100,000** | **cases** | **ASR per 100,000** |
| Global | 1108669.92(980707.98-1285863.91) | 36.28 (42.45 to 31.71) | 283954.63(254321.55-317365.19) | 18.99 (21.74 to 16.69) | 824715.29(707716.86-996500.36) | 53.21 (64.53 to 45.10) |
| Tropical Latin America | 7868.07(7467.28-8280.99) | 8.51 (9.16 to 7.90) | 3048.18(2872.60-3204.11) | 6.54 (7.14 to 5.98) | 4819.89(4506.19-5172.15) | 10.52 (11.68 to 9.49) |
| East Asia | 501526.04(397414.92-644874.01) | 87.00 (111.96 to 67.39) | 65440.20(48971.97-85977.34) | 24.67 (32.96 to 18.26) | 436085.84(337437.48-575981.26) | 145.12 (193.61 to 109.22) |
| High-income Asia Pacific | 11550.58(9596.36-14421.28) | 19.64 (25.69 to 15.41) | 2909.60(2453.92-3539.10) | 10.54 (13.89 to 8.15) | 8640.99(6769.93-11446.64) | 28.19 (39.64 to 20.63) |
| Oceania | 1297.71(812.81-2340.59) | 24.24 (45.85 to 14.22) | 569.94(363.76-970.03) | 21.24 (40.17 to 12.09) | 727.77(413.15-1574.68) | 27.43 (60.32 to 14.64) |
| Southern Latin America | 1421.72(1243.90-1608.38) | 5.35 (6.42 to 4.44) | 515.64(451.46-603.00) | 3.85 (4.86 to 3.07) | 906.08(751.01-1082.12) | 6.85 (8.89 to 5.19) |
| Southeast Asia | 109074.95(88261.20-145364.07) | 38.29 (52.66 to 29.60) | 25365.91(16271.92-34163.99) | 18.13 (24.95 to 11.18) | 83709.04(65367.60-115863.98) | 57.99 (84.02 to 42.95) |
| Australasia | 2177.52(1928.78-2474.93) | 19.18 (23.34 to 15.81) | 777.93(672.97-894.41) | 13.97 (18.05 to 10.80) | 1399.59(1168.92-1668.70) | 24.53 (32.30 to 18.48) |
| Western Europe | 17827.13(17008.88-18635.19) | 12.73 (13.66 to 11.90) | 6316.14(6024.80-6625.78) | 9.14 (9.91 to 8.46) | 11510.99(10813.17-12199.86) | 16.24 (17.97 to 14.79) |
| Andean Latin America | 4130.71(3179.67-5320.29) | 15.28 (21.57 to 10.78) | 1608.88(1139.65-2181.70) | 11.93 (17.27 to 7.88) | 2521.83(1901.30-3303.37) | 18.59 (27.71 to 12.44) |
| Central Latin America | 11999.28(10770.79-13268.98) | 11.88 (13.23 to 10.56) | 5094.86(4412.02-5809.42) | 9.83 (11.30 to 8.37) | 6904.42(6245.92-7548.07) | 14.03 (15.60 to 12.46) |
| High-income North America | 18916.12(18164.72-19723.57) | 14.69 (15.45 to 13.95) | 7029.80(6716.23-7314.51) | 11.04 (11.70 to 10.40) | 11886.33(11243.36-12547.67) | 18.34 (19.57 to 17.14) |
| North Africa and Middle East | 53268.30(45089.77-61982.60) | 20.33 (24.91 to 16.55) | 24418.10(20243.16-29403.52) | 19.54 (24.89 to 15.39) | 28850.20(24228.15-34143.75) | 21.06 (27.00 to 16.44) |
| South Asia | 132492.66(114372.92-158786.19) | 17.14 (21.07 to 14.37) | 51569.56(42250.85-64494.28) | 13.42 (17.26 to 10.63) | 80923.10(67395.51-98891.55) | 20.76 (26.16 to 16.88) |
| Central Europe | 3376.34(3003.02-3757.54) | 8.61 (9.84 to 7.53) | 1004.91(870.16-1154.76) | 5.26 (6.20 to 4.46) | 2371.43(2109.26-2664.24) | 11.79 (13.70 to 10.19) |
| Central Asia | 12035.35(9962.15-14294.99) | 30.50 (37.07 to 24.78) | 5400.98(4490.10-6425.21) | 27.57 (33.84 to 22.35) | 6634.37(5332.47-8038.11) | 33.40 (41.43 to 26.51) |
| Eastern Sub-Saharan Africa | 48233.65(34939.46-67793.32) | 31.04 (44.30 to 21.93) | 24223.04(18180.38-32038.25) | 29.01 (39.27 to 21.21) | 24010.61(16188.14-36561.44) | 33.32 (52.85 to 21.45) |
| Central Sub-Saharan Africa | 13428.07(5825.85-33126.97) | 27.94 (67.55 to 11.46) | 6208.96(2709.42-14499.06) | 25.25 (62.72 to 9.75) | 7219.11(2792.67-21302.68) | 30.68 (90.75 to 10.84) |
| Western Sub-Saharan Africa | 119383.36(91557.87-150479.50) | 71.51 (92.94 to 53.41) | 38867.77(28299.49-50769.38) | 43.26 (58.16 to 30.88) | 80515.58(61653.93-103729.90) | 103.21 (137.99 to 75.74) |
| Eastern Europe | 9748.14(9038.55-10461.60) | 12.57 (13.64 to 11.57) | 3471.76(3089.87-3890.39) | 9.37 (10.51 to 8.26) | 6276.39(5760.33-6835.54) | 15.75 (17.47 to 14.27) |
| Southern Sub-Saharan Africa | 27142.18(21720.77-33787.56) | 78.06 (100.72 to 60.20) | 9405.91(7192.39-11988.78) | 53.92 (74.01 to 37.94) | 17736.27(13547.55-24427.17) | 102.59 (145.05 to 74.35) |
| Caribbean | 1772.06(1396.08-2182.12) | 9.63 (12.60 to 7.37) | 706.57(539.11-975.86) | 7.63 (10.95 to 5.42) | 1065.49(863.73-1293.10) | 11.66 (15.53 to 8.66) |
| **SDI regions** |  |  |  |  |  |  |
| High SDI | 64530.06(61004.47-68416.54) | 16.45 (17.78 to 15.30) | 20030.17(19109.22-20997.59) | 10.77 (11.60 to 10.10) | 44499.89(41111.08-48044.73) | 21.75 (24.14 to 19.70) |
| Low SDI | 149570.64(116850.33-201321.11) | 37.34 (50.97 to 28.92) | 63181.62(48307.73-83225.71) | 29.97 (40.10 to 22.63) | 86389.02(66555.82-124191.21) | 45.12 (64.96 to 33.79) |
| Middle SDI | 466234.38(396473.58-565462.25) | 46.94 (57.86 to 39.13) | 87911.37(75523.40-102245.69) | 18.23 (21.55 to 15.47) | 378323.01(314043.13-478536.55) | 75.09 (95.87 to 60.35) |
| High-middle SDI | 223568.44(179417.14-280346.11) | 42.94 (54.44 to 33.65) | 35085.80(28249.73-43963.08) | 14.52 (18.64 to 11.47) | 188482.63(146084.41-245500.83) | 69.64 (91.32 to 52.90) |
| Low-middle SDI | 204337.76(174406.30-243046.21) | 26.35 (31.82 to 22.00) | 77596.15(67124.45-89912.70) | 19.90 (23.91 to 16.71) | 126741.62(105665.29-154600.67) | 32.79 (41.61 to 25.88) |

**Table S7. Forecast of incidence all-age cases, ASIR, DALYs all-age cases and ASDR for AYA HCC based on disease burden from 1990 to 2021, grouped by sex, projected to 2040**

| Sex | Year | incidence all ages number (95% CI) | ASIR (95% CI) | DALYs all ages number (95% CI) | ASDR (95% CI) |
| --- | --- | --- | --- | --- | --- |
| both | 1990 | 21352.69(20944.51-21760.87) | 0.97(0.96-0.99) | 1092611.19(1089665.55-1095556.83) | 49.85(49.75-49.95) |
| both | 1991 | 22152.53(21741.58-22563.48) | 1(0.98-1.01) | 1106317.38(1103358.39-1109276.36) | 49.77(49.68-49.87) |
| both | 1992 | 22907.53(22490.43-23324.64) | 1.02(1-1.03) | 1142632.69(1139630.63-1145634.74) | 50.73(50.64-50.83) |
| both | 1993 | 23563.33(23140.77-23985.9) | 1.03(1.02-1.05) | 1168155.72(1165124.77-1171186.68) | 51.24(51.14-51.33) |
| both | 1994 | 24077.95(23651.08-24504.83) | 1.04(1.03-1.06) | 1203680.04(1200607.18-1206752.89) | 52.2(52.1-52.29) |
| both | 1995 | 24474(24043.74-24904.26) | 1.05(1.04-1.06) | 1250760.56(1247631.85-1253889.27) | 53.64(53.54-53.73) |
| both | 1996 | 24917.12(24483.19-25351.05) | 1.06(1.04-1.07) | 1269090.7(1265942.91-1272238.48) | 53.79(53.69-53.88) |
| both | 1997 | 25523.99(25085.33-25962.66) | 1.07(1.06-1.08) | 1270817.27(1267671.73-1273962.81) | 53.2(53.11-53.29) |
| both | 1998 | 26267.82(25823.54-26712.1) | 1.08(1.07-1.1) | 1304888.72(1301705.65-1308071.79) | 53.9(53.8-53.99) |
| both | 1999 | 27000.45(26550.65-27450.26) | 1.1(1.09-1.11) | 1350639.71(1347405.07-1353874.35) | 55(54.9-55.09) |
| both | 2000 | 27544.87(27091-27998.75) | 1.11(1.09-1.12) | 1380262.04(1376995.26-1383528.81) | 55.39(55.29-55.48) |
| both | 2001 | 27420.96(26968.54-27873.39) | 1.08(1.07-1.1) | 1384407.22(1381137.66-1387676.78) | 54.75(54.66-54.84) |
| both | 2002 | 26501.39(26057.08-26945.7) | 1.03(1.02-1.05) | 1304692.13(1301519.15-1307865.1) | 50.88(50.79-50.97) |
| both | 2003 | 25197.2(24764.11-25630.29) | 0.97(0.96-0.98) | 1216271.29(1213207.47-1219335.1) | 46.83(46.74-46.91) |
| both | 2004 | 24011.34(23588.38-24434.31) | 0.91(0.9-0.92) | 1170362.36(1167355.76-1173368.96) | 44.52(44.44-44.6) |
| both | 2005 | 23401.49(22983.9-23819.09) | 0.88(0.87-0.89) | 1138681.03(1135714.15-1141647.92) | 42.83(42.75-42.91) |
| both | 2006 | 23329.03(22912.33-23745.72) | 0.87(0.86-0.88) | 1114357.87(1111422.28-1117293.47) | 41.47(41.4-41.55) |
| both | 2007 | 23404.44(22987.16-23821.71) | 0.86(0.85-0.87) | 1112230.85(1109297.63-1115164.08) | 41(40.92-41.08) |
| both | 2008 | 23531.31(23112.82-23949.8) | 0.86(0.85-0.87) | 1132392.41(1129432.29-1135352.53) | 41.36(41.28-41.44) |
| both | 2009 | 23603.81(23184.5-24023.11) | 0.85(0.84-0.87) | 1134442.69(1131479.54-1137405.83) | 41.08(41-41.15) |
| both | 2010 | 23500.84(23082.43-23919.25) | 0.84(0.83-0.86) | 1118796.16(1115853.96-1121738.37) | 40.21(40.14-40.29) |
| both | 2011 | 23202.81(22787.24-23618.37) | 0.83(0.82-0.84) | 1071150.92(1068272.75-1074029.1) | 38.26(38.19-38.34) |
| both | 2012 | 22825.34(22413.45-23237.22) | 0.81(0.8-0.82) | 1037173.7(1034342.45-1040004.95) | 36.83(36.76-36.9) |
| both | 2013 | 22470.37(22062.05-22878.69) | 0.79(0.78-0.8) | 1039664.03(1036831.01-1042497.06) | 36.7(36.63-36.77) |
| both | 2014 | 22227.77(21822.06-22633.47) | 0.78(0.77-0.79) | 1046887.78(1044047.66-1049727.91) | 36.75(36.67-36.82) |
| both | 2015 | 22151.68(21747.2-22556.15) | 0.77(0.76-0.78) | 1042089.94(1039260.03-1044919.84) | 36.36(36.29-36.43) |
| both | 2016 | 22242.83(21838.16-22647.5) | 0.77(0.76-0.78) | 1038527.64(1035706.55-1041348.73) | 36.01(35.94-36.08) |
| both | 2017 | 22436.87(22031.07-22842.67) | 0.77(0.76-0.78) | 1049520.49(1046688.4-1052352.57) | 36.17(36.1-36.23) |
| both | 2018 | 22690.74(22283.22-23098.25) | 0.78(0.77-0.79) | 1054079.35(1051245.07-1056913.64) | 36.1(36.03-36.16) |
| both | 2019 | 22968.73(22559.23-23378.23) | 0.78(0.77-0.79) | 1058033.74(1055197.79-1060869.7) | 36.01(35.94-36.08) |
| both | 2020 | 23282.17(22870.21-23694.13) | 0.79(0.78-0.8) | 1067766.31(1064920.52-1070612.09) | 36.12(36.05-36.19) |
| both | 2021 | 23527.16(23110.12-23944.2) | 0.79(0.78-0.8) | 1076451.6(1073596.46-1079306.74) | 36.19(36.12-36.25) |
| both | 2022 | 24132.43(22625.8-25639.05) | 0.79(0.75-0.84) | 1099687.82(1022531.88-1176843.76) | 36.17(33.63-38.7) |
| both | 2023 | 24239.16(22296.43-26181.88) | 0.79(0.73-0.86) | 1101289.45(1003170.98-1199407.93) | 36.04(32.83-39.26) |
| both | 2024 | 24264.98(21954.8-26575.15) | 0.79(0.72-0.87) | 1099605.63(983748.82-1215462.44) | 35.84(32.06-39.62) |
| both | 2025 | 24189.53(21558.96-26820.11) | 0.79(0.7-0.87) | 1094370.27(962939.84-1225800.7) | 35.54(31.27-39.8) |
| both | 2026 | 24012.84(21095.95-26929.72) | 0.78(0.68-0.87) | 1085659.64(940195.33-1231123.95) | 35.13(30.42-39.83) |
| both | 2027 | 23760.05(20579.31-26940.8) | 0.77(0.66-0.87) | 1074491(915969.67-1233012.33) | 34.64(29.53-39.75) |
| both | 2028 | 23465.84(20043.4-26888.27) | 0.75(0.64-0.86) | 1062127.95(891427.05-1232828.84) | 34.13(28.64-39.61) |
| both | 2029 | 23171.69(19521.99-26821.39) | 0.74(0.63-0.86) | 1049953.51(867600.33-1232306.7) | 33.63(27.79-39.47) |
| both | 2030 | 22917.45(19043.35-26791.54) | 0.73(0.61-0.86) | 1039790.97(845726.02-1233855.92) | 33.21(27.01-39.41) |
| both | 2031 | 22720.72(18614.36-26827.08) | 0.72(0.59-0.85) | 1032344.84(825985.13-1238704.55) | 32.87(26.3-39.44) |
| both | 2032 | 22586.67(18230.83-26942.51) | 0.72(0.58-0.85) | 1027472.21(807831.85-1247112.57) | 32.59(25.63-39.56) |
| both | 2033 | 22498.49(17877.42-27119.56) | 0.71(0.57-0.86) | 1023786.22(790067.76-1257504.68) | 32.36(24.97-39.75) |
| both | 2034 | 22453.17(17546.17-27360.17) | 0.71(0.55-0.86) | 1021125.11(772318.85-1269931.36) | 32.16(24.32-40) |
| both | 2035 | 22433.04(17214.38-27651.7) | 0.7(0.54-0.87) | 1019052.76(753853.95-1284251.58) | 31.98(23.66-40.3) |
| both | 2036 | 22419.25(16856.1-27982.4) | 0.7(0.53-0.87) | 1017063.18(733764.92-1300361.44) | 31.8(22.94-40.66) |
| both | 2037 | 22397.64(16448.06-28347.23) | 0.7(0.51-0.88) | 1014712.39(711152.46-1318272.32) | 31.62(22.16-41.08) |
| both | 2038 | 22365.51(15980.54-28750.48) | 0.69(0.5-0.89) | 1011894.8(685697.14-1338092.46) | 31.44(21.3-41.57) |
| both | 2039 | 22323.8(15442.33-29205.28) | 0.69(0.48-0.9) | 1008673.33(657025.01-1360321.66) | 31.25(20.36-42.15) |
| both | 2040 | 22274.45(14819.36-29729.54) | 0.69(0.46-0.92) | 1005173.39(624627.91-1385718.87) | 31.07(19.31-42.83) |
| female | 1990 | 5311.85(5117.77-5505.93) | 0.49(0.48-0.5) | 277878.98(276400.21-279357.74) | 25.65(25.55-25.75) |
| female | 1991 | 5422.16(5232.34-5611.99) | 0.49(0.48-0.5) | 278367.64(276890.43-279844.85) | 25.34(25.25-25.44) |
| female | 1992 | 5537.25(5346.4-5728.11) | 0.5(0.49-0.51) | 285513.43(284019.56-287007.3) | 25.66(25.56-25.75) |
| female | 1993 | 5642.16(5449.68-5834.64) | 0.5(0.49-0.51) | 290112.15(288608.21-291616.09) | 25.75(25.66-25.85) |
| female | 1994 | 5731.03(5537.04-5925.03) | 0.5(0.49-0.51) | 298727.8(297203.08-300252.52) | 26.21(26.11-26.3) |
| female | 1995 | 5807.15(5611.76-6002.54) | 0.5(0.49-0.51) | 304787.4(303248.68-306326.12) | 26.43(26.33-26.52) |
| female | 1996 | 5887.44(5690.56-6084.33) | 0.5(0.49-0.52) | 307967.18(306421.91-309512.44) | 26.38(26.29-26.47) |
| female | 1997 | 5977.3(5778.85-6175.76) | 0.51(0.49-0.52) | 310126.6(308577.55-311675.66) | 26.22(26.13-26.32) |
| female | 1998 | 6075.27(5875.25-6275.29) | 0.51(0.5-0.52) | 313878.06(312321.18-315434.94) | 26.18(26.08-26.27) |
| female | 1999 | 6163.59(5962.17-6365.01) | 0.51(0.5-0.52) | 318919.94(317352.02-320487.86) | 26.21(26.12-26.3) |
| female | 2000 | 6221.25(6018.86-6423.64) | 0.5(0.49-0.51) | 322275.23(320700.12-323850.34) | 26.09(26-26.18) |
| female | 2001 | 6189.97(5988.34-6391.6) | 0.49(0.48-0.5) | 322398.19(320823.4-323972.98) | 25.72(25.63-25.81) |
| female | 2002 | 6054.12(5855.23-6253.02) | 0.48(0.47-0.49) | 309422.92(307880.57-310965.28) | 24.34(24.25-24.43) |
| female | 2003 | 5871.93(5676.28-6067.58) | 0.46(0.45-0.47) | 294047.92(292544.4-295551.45) | 22.84(22.75-22.92) |
| female | 2004 | 5710.34(5517.35-5903.32) | 0.44(0.43-0.45) | 287213.86(285727.58-288700.15) | 22.04(21.96-22.12) |
| female | 2005 | 5627.23(5435.79-5818.66) | 0.43(0.42-0.44) | 282285.35(280811.52-283759.18) | 21.42(21.34-21.5) |
| female | 2006 | 5606.97(5416.17-5797.78) | 0.42(0.41-0.43) | 278082.82(276619.85-279545.79) | 20.88(20.81-20.96) |
| female | 2007 | 5596.28(5405.81-5786.75) | 0.42(0.41-0.43) | 275322.58(273866.83-276778.34) | 20.48(20.41-20.56) |
| female | 2008 | 5591.19(5400.87-5781.51) | 0.41(0.4-0.42) | 277764.42(276302.22-279226.63) | 20.48(20.41-20.56) |
| female | 2009 | 5587.92(5397.68-5778.16) | 0.41(0.4-0.42) | 279150.9(277685.22-280616.58) | 20.41(20.34-20.49) |
| female | 2010 | 5574.81(5384.82-5764.79) | 0.4(0.4-0.41) | 276922.59(275463.17-278382) | 20.1(20.03-20.18) |
| female | 2011 | 5540.77(5351.47-5730.06) | 0.4(0.39-0.41) | 269081.94(267643.75-270520.14) | 19.41(19.34-19.49) |
| female | 2012 | 5491.34(5303.06-5679.61) | 0.39(0.39-0.4) | 264727.47(263301.38-266153.56) | 18.99(18.91-19.06) |
| female | 2013 | 5442.91(5255.64-5630.17) | 0.39(0.38-0.4) | 266064.98(264635.86-267494.09) | 18.98(18.91-19.05) |
| female | 2014 | 5413.1(5226.51-5599.69) | 0.38(0.38-0.39) | 266267.48(264838.74-267696.22) | 18.89(18.82-18.96) |
| female | 2015 | 5411.06(5224.65-5597.48) | 0.38(0.37-0.39) | 265947.06(264520.45-267373.68) | 18.77(18.69-18.84) |
| female | 2016 | 5438.01(5251.26-5624.77) | 0.38(0.37-0.39) | 268742.24(267309.51-270174.97) | 18.86(18.79-18.93) |
| female | 2017 | 5479.07(5291.7-5666.43) | 0.38(0.37-0.39) | 270982.26(269544.87-272419.65) | 18.91(18.84-18.98) |
| female | 2018 | 5525.85(5337.74-5713.95) | 0.38(0.38-0.39) | 270753.91(269318.4-272189.42) | 18.79(18.72-18.86) |
| female | 2019 | 5577.13(5388.1-5766.17) | 0.38(0.38-0.39) | 271577.92(270141.32-273014.51) | 18.74(18.67-18.81) |
| female | 2020 | 5628.17(5437.58-5818.77) | 0.39(0.38-0.39) | 273813.42(272371.77-275255.07) | 18.8(18.73-18.87) |
| female | 2021 | 5692.68(5495.86-5889.5) | 0.39(0.38-0.4) | 277790.44(276338.28-279242.61) | 18.96(18.89-19.03) |
| female | 2022 | 5786.01(5453.95-6118.07) | 0.39(0.37-0.41) | 280767.78(264007.33-297528.23) | 18.75(17.64-19.87) |
| female | 2023 | 5802.46(5401.35-6203.57) | 0.39(0.36-0.41) | 281886.14(261233.54-302538.74) | 18.75(17.38-20.13) |
| female | 2024 | 5813.4(5349.79-6277) | 0.39(0.36-0.41) | 282786.87(258635.05-306938.69) | 18.75(17.15-20.35) |
| female | 2025 | 5819.78(5297.41-6342.15) | 0.38(0.35-0.42) | 283511.6(256069.93-310953.26) | 18.75(16.93-20.56) |
| female | 2026 | 5823.42(5243.75-6403.09) | 0.38(0.35-0.42) | 284137.86(253480.05-314795.68) | 18.74(16.72-20.76) |
| female | 2027 | 5826.42(5189.12-6463.72) | 0.38(0.34-0.42) | 284789.9(250877.06-318702.74) | 18.73(16.5-20.96) |
| female | 2028 | 5829.3(5133.7-6524.9) | 0.38(0.34-0.43) | 285511.25(248271.67-322750.82) | 18.73(16.29-21.18) |
| female | 2029 | 5833.96(5078.27-6589.64) | 0.38(0.33-0.43) | 286365.09(245664.72-327065.46) | 18.75(16.09-21.42) |
| female | 2030 | 5844.85(5025.36-6664.34) | 0.38(0.33-0.43) | 287546.31(243147.78-331944.83) | 18.8(15.9-21.7) |
| female | 2031 | 5865.12(4975.94-6754.31) | 0.38(0.33-0.44) | 289165.7(240719.64-337611.75) | 18.86(15.71-22.02) |
| female | 2032 | 5897.84(4930.77-6864.92) | 0.38(0.32-0.45) | 291332.49(238371.05-344293.93) | 18.95(15.51-22.4) |
| female | 2033 | 5937.33(4883.88-6990.78) | 0.39(0.32-0.45) | 293744.28(235800.77-351687.78) | 19.06(15.3-22.82) |
| female | 2034 | 5984.36(4834.24-7134.49) | 0.39(0.31-0.46) | 296437.46(232964.98-359909.93) | 19.18(15.07-23.29) |
| female | 2035 | 6037.71(4778.63-7296.78) | 0.39(0.31-0.47) | 299371.47(229734.13-369008.82) | 19.31(14.82-23.81) |
| female | 2036 | 6095.64(4713.16-7478.12) | 0.39(0.3-0.48) | 302478.67(225941.34-379016) | 19.46(14.53-24.38) |
| female | 2037 | 6156.16(4633.78-7678.54) | 0.39(0.3-0.49) | 305660.5(221396.37-389924.64) | 19.61(14.2-25.01) |
| female | 2038 | 6217.29(4537.3-7897.28) | 0.4(0.29-0.5) | 308798.49(215944.1-401652.88) | 19.76(13.82-25.7) |
| female | 2039 | 6277.85(4420.93-8134.76) | 0.4(0.28-0.52) | 311848.92(209472.86-414224.97) | 19.9(13.37-26.44) |
| female | 2040 | 6337.17(4282.27-8392.07) | 0.4(0.27-0.53) | 314805.14(201888.5-427721.78) | 20.05(12.86-27.24) |
| male | 1990 | 16057.08(15703.29-16410.88) | 1.45(1.43-1.47) | 813807.31(811262.81-816351.81) | 73.42(73.25-73.58) |
| male | 1991 | 16712.85(16356.49-17069.22) | 1.49(1.46-1.51) | 826958.57(824398.33-829518.81) | 73.55(73.39-73.71) |
| male | 1992 | 17337.52(16975.27-17699.77) | 1.52(1.5-1.54) | 856033.82(853433.67-858633.97) | 75.13(74.97-75.3) |
| male | 1993 | 17882.51(17515.02-18250) | 1.55(1.53-1.57) | 876917.52(874289.89-879545.16) | 76.04(75.88-76.2) |
| male | 1994 | 18308.44(17936.83-18680.04) | 1.57(1.55-1.59) | 903824.48(901160.41-906488.55) | 77.51(77.35-77.68) |
| male | 1995 | 18635.89(18261.06-19010.72) | 1.58(1.56-1.6) | 944927.43(942206.8-947648.05) | 80.17(80.01-80.34) |
| male | 1996 | 19007.03(18628.64-19385.42) | 1.59(1.57-1.62) | 960337.22(957597.77-963076.66) | 80.57(80.4-80.73) |
| male | 1997 | 19526.87(19143.76-19909.98) | 1.62(1.6-1.64) | 960211.89(957476.47-962947.3) | 79.61(79.45-79.77) |
| male | 1998 | 20173.38(19784.56-20562.19) | 1.65(1.63-1.67) | 990798.75(988024.04-993573.46) | 81.09(80.92-81.25) |
| male | 1999 | 20814.02(20419.56-21208.48) | 1.68(1.66-1.7) | 1031710.18(1028882.08-1034538.28) | 83.27(83.11-83.43) |
| male | 2000 | 21292.13(20893.51-21690.76) | 1.69(1.67-1.72) | 1058205.12(1055343.75-1061066.48) | 84.19(84.03-84.35) |
| male | 2001 | 21203.81(20806.36-21601.26) | 1.66(1.64-1.68) | 1062541.28(1059675.87-1065406.68) | 83.32(83.17-83.48) |
| male | 2002 | 20449.03(20059.12-20838.93) | 1.58(1.56-1.6) | 996113.55(993339.95-998887.15) | 77.04(76.89-77.19) |
| male | 2003 | 19361.65(18982.41-19740.88) | 1.48(1.46-1.5) | 923268.48(920597.78-925939.18) | 70.49(70.35-70.64) |
| male | 2004 | 18362.61(17993.17-18732.05) | 1.39(1.37-1.4) | 884303.4(881688.4-886918.4) | 66.71(66.57-66.85) |
| male | 2005 | 17833.2(17469.11-18197.29) | 1.33(1.31-1.35) | 857583.16(855006.67-860159.65) | 63.96(63.82-64.1) |
| male | 2006 | 17763.16(17400.03-18126.29) | 1.31(1.29-1.33) | 837367.72(834821.28-839914.16) | 61.78(61.65-61.92) |
| male | 2007 | 17837.44(17473.61-18201.27) | 1.3(1.28-1.32) | 837996.96(835449.11-840544.81) | 61.22(61.09-61.36) |
| male | 2008 | 17966.24(17600.93-18331.54) | 1.3(1.28-1.32) | 855895.9(853320.39-858471.42) | 61.94(61.81-62.07) |
| male | 2009 | 18042.94(17676.6-18409.28) | 1.29(1.28-1.31) | 856868.07(854290.34-859445.79) | 61.46(61.32-61.59) |
| male | 2010 | 17955.18(17589.66-18320.7) | 1.28(1.26-1.3) | 843683.97(841126.23-846241.71) | 60.07(59.94-60.2) |
| male | 2011 | 17695.82(17333.12-18058.51) | 1.25(1.23-1.27) | 803999.6(801503.2-806496.01) | 56.89(56.76-57.01) |
| male | 2012 | 17375.71(17016.57-17734.84) | 1.22(1.2-1.24) | 774446.02(771996.65-776895.38) | 54.47(54.34-54.59) |
| male | 2013 | 17077.54(16721.79-17433.28) | 1.19(1.18-1.21) | 775693.97(773244.08-778143.85) | 54.22(54.1-54.34) |
| male | 2014 | 16870.5(16517.23-17223.77) | 1.17(1.15-1.19) | 782846.69(780388.08-785305.3) | 54.38(54.26-54.5) |
| male | 2015 | 16796.53(16444.47-17148.58) | 1.16(1.14-1.18) | 778463.66(776015.42-780911.89) | 53.72(53.6-53.84) |
| male | 2016 | 16860.95(16508.81-17213.09) | 1.16(1.14-1.17) | 772149.22(769714.69-774583.75) | 52.93(52.81-53.05) |
| male | 2017 | 17014.4(16661.26-17367.54) | 1.16(1.14-1.17) | 780973.41(778528.74-783418.09) | 53.16(53.05-53.28) |
| male | 2018 | 17219.67(16864.94-17574.4) | 1.16(1.15-1.18) | 785804.76(783356.36-788253.15) | 53.12(53.01-53.24) |
| male | 2019 | 17447.58(17090.97-17804.2) | 1.17(1.16-1.19) | 788927.47(786477.82-791377.12) | 52.97(52.86-53.09) |
| male | 2020 | 17709.02(17350.05-18067.99) | 1.18(1.16-1.2) | 796401.78(793943.74-798859.81) | 53.11(53-53.23) |
| male | 2021 | 17878.73(17515.27-18242.18) | 1.18(1.17-1.2) | 801073.47(798610.45-803536.49) | 53.06(52.95-53.18) |
| male | 2022 | 18455.52(17184.44-19726.61) | 1.2(1.12-1.28) | 827717.3(764031.48-891403.13) | 53.62(49.5-57.75) |
| male | 2023 | 18537.97(16898.8-20177.13) | 1.19(1.09-1.3) | 827383.75(746694.38-908073.11) | 53.3(48.1-58.5) |
| male | 2024 | 18532.95(16587.17-20478.73) | 1.19(1.06-1.31) | 823412.99(728578.43-918247.56) | 52.78(46.7-58.86) |
| male | 2025 | 18420.02(16212.41-20627.62) | 1.18(1.04-1.32) | 815515.34(708601.33-922429.35) | 52.04(45.21-58.86) |
| male | 2026 | 18199.44(15764.67-20634.22) | 1.16(1-1.31) | 803669.56(686298.17-921040.96) | 51.05(43.6-58.51) |
| male | 2027 | 17895.21(15258.3-20532.13) | 1.13(0.97-1.3) | 788843.49(662197.77-915489.22) | 49.88(41.87-57.89) |
| male | 2028 | 17540.28(14727.07-20353.49) | 1.1(0.93-1.28) | 772301.39(637520.21-907082.58) | 48.62(40.14-57.11) |
| male | 2029 | 17173.82(14203.36-20144.28) | 1.08(0.89-1.26) | 755519.07(613425.45-897612.7) | 47.37(38.46-56.28) |
| male | 2030 | 16830.76(13712.17-19949.35) | 1.05(0.86-1.25) | 740278.47(591171.69-889385.26) | 46.23(36.92-55.55) |
| male | 2031 | 16525.84(13259.62-19792.07) | 1.03(0.83-1.23) | 727258.49(571010.06-883506.92) | 45.23(35.52-54.95) |
| male | 2032 | 16263.73(12843.23-19684.23) | 1.01(0.8-1.22) | 716208.94(552428.72-879989.15) | 44.34(34.2-54.47) |
| male | 2033 | 16040.98(12459.79-19622.17) | 0.99(0.77-1.21) | 706188.81(534677.33-877700.28) | 43.53(32.95-54.1) |
| male | 2034 | 15854.62(12103-19606.25) | 0.97(0.74-1.2) | 696903.84(517389.15-876418.53) | 42.76(31.75-53.78) |
| male | 2035 | 15684.37(11750.79-19617.95) | 0.96(0.72-1.2) | 687773.62(499873.52-875673.71) | 42.02(30.54-53.5) |
| male | 2036 | 15510.46(11380.15-19640.77) | 0.94(0.69-1.19) | 678264.59(481383.37-875145.82) | 41.27(29.29-53.25) |
| male | 2037 | 15323.23(10975.12-19671.35) | 0.93(0.67-1.19) | 668181.93(461386.23-874977.63) | 40.5(27.97-53.03) |
| male | 2038 | 15129.36(10535.03-19723.69) | 0.91(0.64-1.19) | 657991.29(440077.44-875905.14) | 39.74(26.58-52.91) |
| male | 2039 | 14932.88(10053.82-19811.94) | 0.9(0.61-1.19) | 647861.4(417270.75-878452.04) | 39.02(25.13-52.9) |
| male | 2040 | 14735.26(9521.03-19949.48) | 0.89(0.57-1.2) | 637881.17(392561.07-883201.28) | 38.32(23.58-53.05) |
